# Asymmetric Alkylation of Cyclic Ketones with Dehydroalanine via H‐Bond‐Directing Enamine Catalysis: Straightforward Access to Enantiopure Unnatural α‐Amino Acids

**DOI:** 10.1002/chem.202201994

**Published:** 2022-08-18

**Authors:** Michele Retini, Silvia Bartolucci, Francesca Bartoccini, Giovanni Piersanti

**Affiliations:** ^1^ Department of Biomolecular Sciences University of Urbino Carlo Bo Piazza Rinascimento 6 61029 Urbino PU Italy

**Keywords:** conjugate addition, ketones, noncovalent Interactions, organocatalysis, unnatural α-amino acids

## Abstract

The growing importance of structurally diverse and functionalized enantiomerically pure unnatural amino acids in the design of drugs, including peptides, has stimulated the development of new synthetic methods. This study reports the challenging direct asymmetric alkylation of cyclic ketones with dehydroalanine derivatives via a conjugate addition reaction for the synthesis of enantiopure ketone‐based α‐unnatural amino acids. The key to success was the design of a bifunctional primary amine‐thiourea catalyst that combines H‐bond‐directing activation and enamine catalysis. The simultaneous dual activation of the two relatively unreactive partners, confirmed by mass spectrometry studies, results in high reactivity while securing high levels of stereocontrol. A broad substrate scope is accompanied by versatile downstream chemical modifications. The mild reaction conditions and consistently excellent enantioselectivities (>95 % ee in most cases) render this protocol highly practical for the rapid construction of valuable noncanonical enantiopure α‐amino‐acid building blocks.

## Graphical abstract

**A direct, highly enantioselective** organocatalytic alkylation of cyclic ketones with dehydroalanine via a conjugate addition reaction is reported. The use of a chiral bifunctional primary amine/thiourea catalyst was key to obtain the enantiopure, functionalized (substituted ketone) unnatural α‐amino acids in good yields and remarkable selectivity. The mechanism of the reactions was explored by mass spectrometry studies.

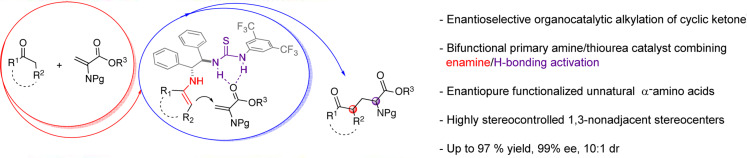

## Introduction

Enantiopure unnatural α‐amino acids (also called nonproteinogenic, noncoded, or noncanonical amino acids) continue to attract interest due to their wide applicability in engineering peptide drugs and as essential building blocks in biologically active natural products.^[1]^ The incorporation of unnatural α‐amino acids generates modifications in the secondary structure of these peptides; as a result, improvements in selectivity, bioavailability, and stability can often be achieved.^[1b]^ The versatility of these unnatural amino acids stems from their utility as chiral frameworks and ability to provide bioconjugation sites orthogonal to native functional groups, thereby allowing the controlled production of homogeneous, site‐specific adducts.^[1c]^ They are also extensively used as a chiral pool for ligand preparation and chiral catalyst design owing to their wide structural diversity.[^1d^, ^1e^, ^1f^, ^1g^] Furthermore, they are versatile intermediates in organic synthesis for drug discovery and even marketed drugs, providing broad utility as one of the most important classes of organic molecules.^[2]^ Therefore, increasing the availability of such amino acid derivatives and the development of straightforward methodologies for their synthesis have become important goals.^[3]^

Catalytic asymmetric routes to unnatural α‐amino acids are especially attractive,^[4]^ and a variety of catalytic technologies and elegant asymmetric catalyst designs have been described, such as hydrogenations of olefins and imines,^[5]^ electrophilic aminations of enolates,^[6]^ electrophilic alkylations of glycine derivatives,^[7]^ nucleophilic additions to α‐imino esters,^[8]^ and, obviously, hydrocyanation of an imine or imine equivalent (the venerable Strecker synthesis).^[9]^ Nevertheless, numerous classes of unnatural α‐amino acids remain unexploited, and their enantioselective syntheses in a straightforward manner from readily available starting materials and effective catalytic systems are urgently needed.^[3f]^ In this context, the direct enantioselective catalytic transformation of prochiral dehydroalanine represents one of the most convergent and efficient routes for the synthesis of enantioenriched unnatural α‐amino acids.^[10]^ To date, however, this class of Michael acceptors with α,β‐unsaturated esters substituted with free or protected amino groups at the α‐position has remained a persistent challenge in enantioselective/asymmetric catalysis due to their low inherent electrophilicity,^[11]^ low propensity for catalyst activation,^[12]^ and high tendency to tautomerize to the ketoimine form under acidic conditions.^[13]^ To the best of our knowledge, organocatalytic enantioselective Michael additions to dehydroalanine have been limited to N‐heterocyclic carbene catalysis of aldehydes by the Stetter reaction,^[14]^ Brønsted base catalysis of 3‐substituted oxindoles,^[15]^ and thiols.^[16]^ Examples of asymmetric aminocatalytic activation modes have yet to be reported despite remarkable reports of highly reactive Michael acceptors, such as α,β‐unsaturated aldehydes, ketones, maleimides, nitroolefins, and vinyl sulfones.^[17]^ Therefore, we postulated that the poor reactivity of dehydroalanine esters in organocatalyzed addition reactions of pronucleophiles toward nucleophilic addition may be overcome by using a chiral bifunctional catalyst that enables the simultaneous activation of donors and acceptors.^[18]^

As the pronucleophile, we chose relatively low‐reactive cyclic ketones for the following reasons: a) they are fundamental building blocks in organic synthesis and can be utilized in a diverse array of powerful bond‐forming reactions (e. g., to form C−C, C=C, C=N, and ROC=O bonds); b) they are a common structural element found in a wide range of bioactive natural products and pharmaceuticals such as tropinone, muscone, santonin, progesterone, cortisone, etc. and are considered as a biocompatible functional group; c) they are versatile electrophilic handles for the chemoselective modification of engineered peptides/proteins with a wide variety of exogenous groups for potential biocompatible/bioorthogonal reactions; and d) they are challenging substrates in asymmetric aminocatalytic reactions due to slow enamine formation, at least when a secondary amine is used as the catalyst.

**Scheme 1:**
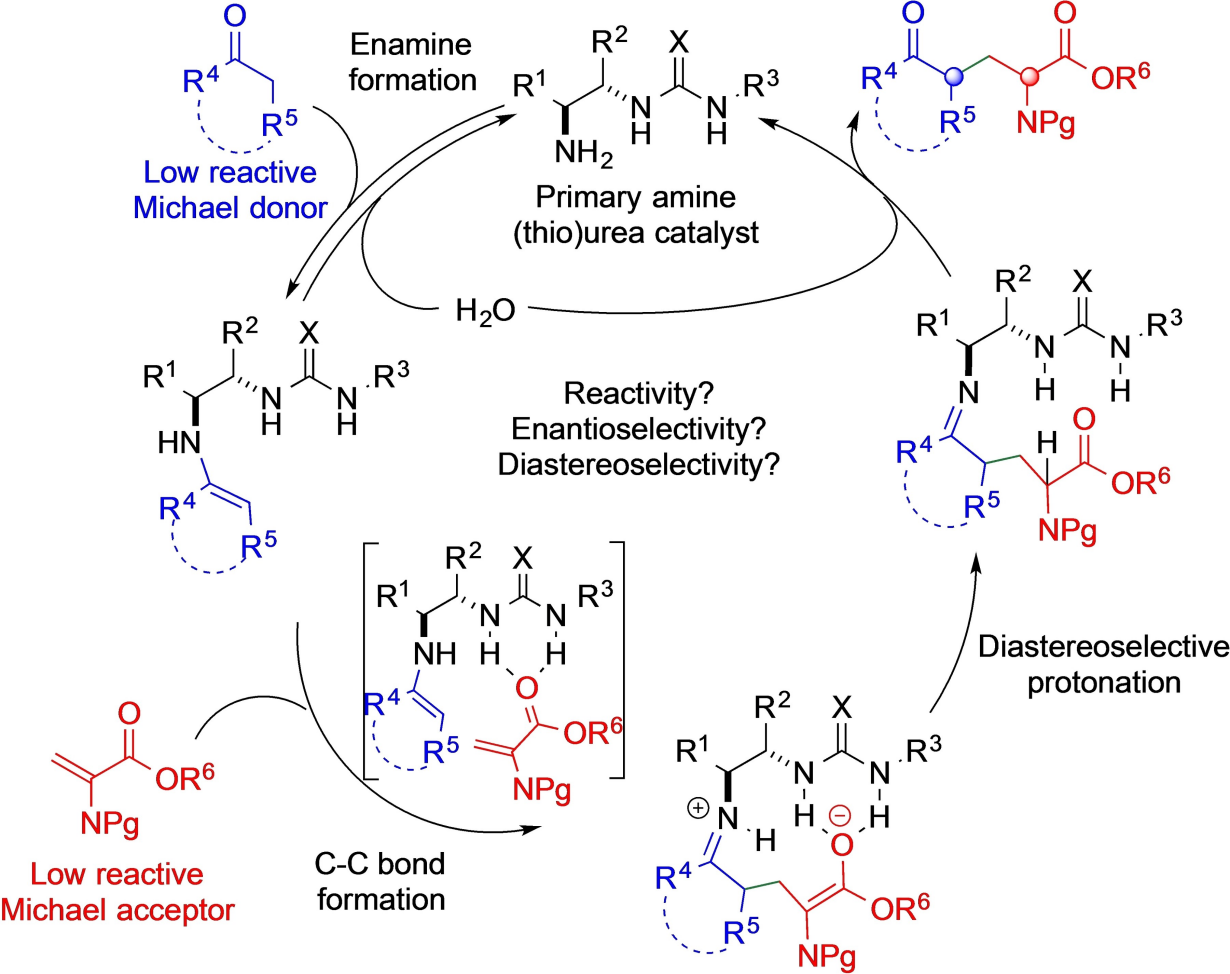
Proposed primary amine bifunctional catalyst that catalyzes the diastereoselective and enantioselective Michael addition of ketones to dehydroalanine derivatives.

Despite the potential broad‐spectrum utility of ketone‐containing α‐amino acid compounds, it is notable to consider that only one (bio)catalytic, engineered β‐subunit of tryptophan synthase by the directed evolution method exists so far for the asymmetric installation of an amino acid side chain onto prostereogenic ketones with an L‐serine‐derived dehydroalanine species.^[19]^ Based on this background, we envisioned that primary amine (thio)urea catalysts, which can be easily prepared from inexpensive and commercially available chiral diamines, can activate cyclic ketones via an enamine intermediate and dehydroalanine via an H‐bonding pathway as a double and synergetic catalyst. Following C−C bond formation/alkylation,^[20]^ selective diastereo‐facial protonation of the transient enolate intermediate would deliver the enantioenriched Michael adduct, water, and the catalyst back into the cycle (Scheme 1). Herein, we describe the development of a direct diastereoselective and enantioselective conjugate addition of cyclic ketones with protected dehydroalanine. The key to the success of this reaction is the use of a bifunctional primary amine‐thiourea catalyst that combines the H‐bond‐directing activation of the reluctant electrophilic dehydroalanine with enamine activation of the ketone donor.

## Results and Discussion

We selected the combination of commercially available cyclohexanone (**1 a**) and the straightforwardly prepared *tert*‐butyl‐2‐phthalimidoacrylate (**2 a**) as the Michael acceptor^[21]^ as the model reaction for the conjugate addition reaction (Table 1). The potential of two different families of bifunctional primary amine‐(thio)urea catalysts, simply derived from cyclohexanediamine (**A1**–**4**) or from (*R,R*)‐1,2‐diphenylethylenediamine (**B1**–**4**), was explored in the abovementioned model reaction. While the two simple thiourea derivatives belonging to the first class of catalysts (**A1**–**2**, entries 2–3) were able to promote the reaction in a low yield and total absence of stereocontrol, (*R,R*)‐1,2‐diphenylethylenediamine‐derived thiourea **B1** clearly induced a great acceleration of the reaction rate, even at ambient temperature, allowing the formation of the product **3 a** with good diastereoselectivity and excellent enantioselectivity (entry 6). In order to gain insight into the mechanism of stereoinduction and to identify the role of each functional group of the catalyst, we initiated a short catalyst structure/reactivity and stereoselectivity correlation study using modified amine‐(thio)urea derivatives.

**Table 1:** Optimization of reaction condition of asymmetric alkylation of cyclic ketones with dehydroalanine.^[a]^

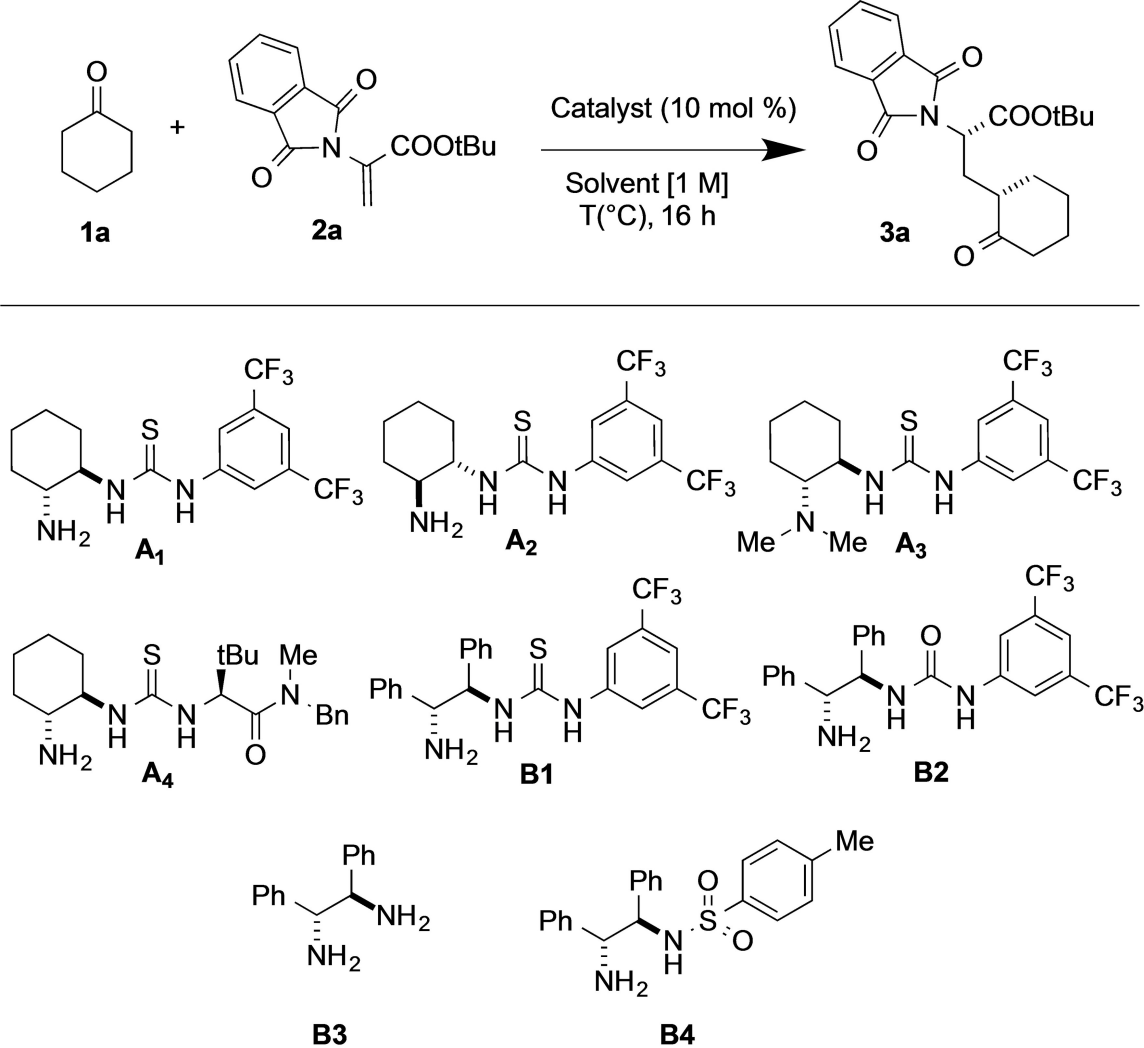						
Entry	Catalyst	Solvent	T [°C]	Yield^[b]^ [%]	dr^[c]^ syn/anti	ee^[d]^ [%]
1	–	toluene	60	NR	–	–
2	**A1**	toluene	30	18	2 : 1	<5
3	**A2**	toluene	30	18	1.6 : 1	<5
4	**A3**	toluene	30	NR	–	–
5	**A4**	toluene	30	NR	–	–
6	**B1**	toluene	30	97	9 : 1	99
7	**B2**	toluene	30	81	8 : 1	87
8	**B3**	toluene	30	NR	–	–
9	**B4**	toluene	30	NR	–	–
10	**B1**	toluene	90	97	1.5 : 1	99
11	**B1**	toluene	60	97	2.5 : 1	99
12	**B1**	toluene	15	38	10 : 1	99
13	**B1**	DCM	30	62	3 : 1	97
14	**B1**	CH_3_CN	30	69	4 : 1	96
15	**B1**	CHCl_3_	30	60	4 : 1	98
16	**B1**	MTBE	30	83	8 : 1	99
17	**B1**	*m*‐xylene	30	70	5 : 1	99
18^[e]^	**B1**	toluene	30	60	9 : 1	99
19^[f]^	**B1**	toluene	30	65	3 : 1	99
20^[g]^	**B1**	toluene	30	79	7 : 1	99

**Scheme 2:**
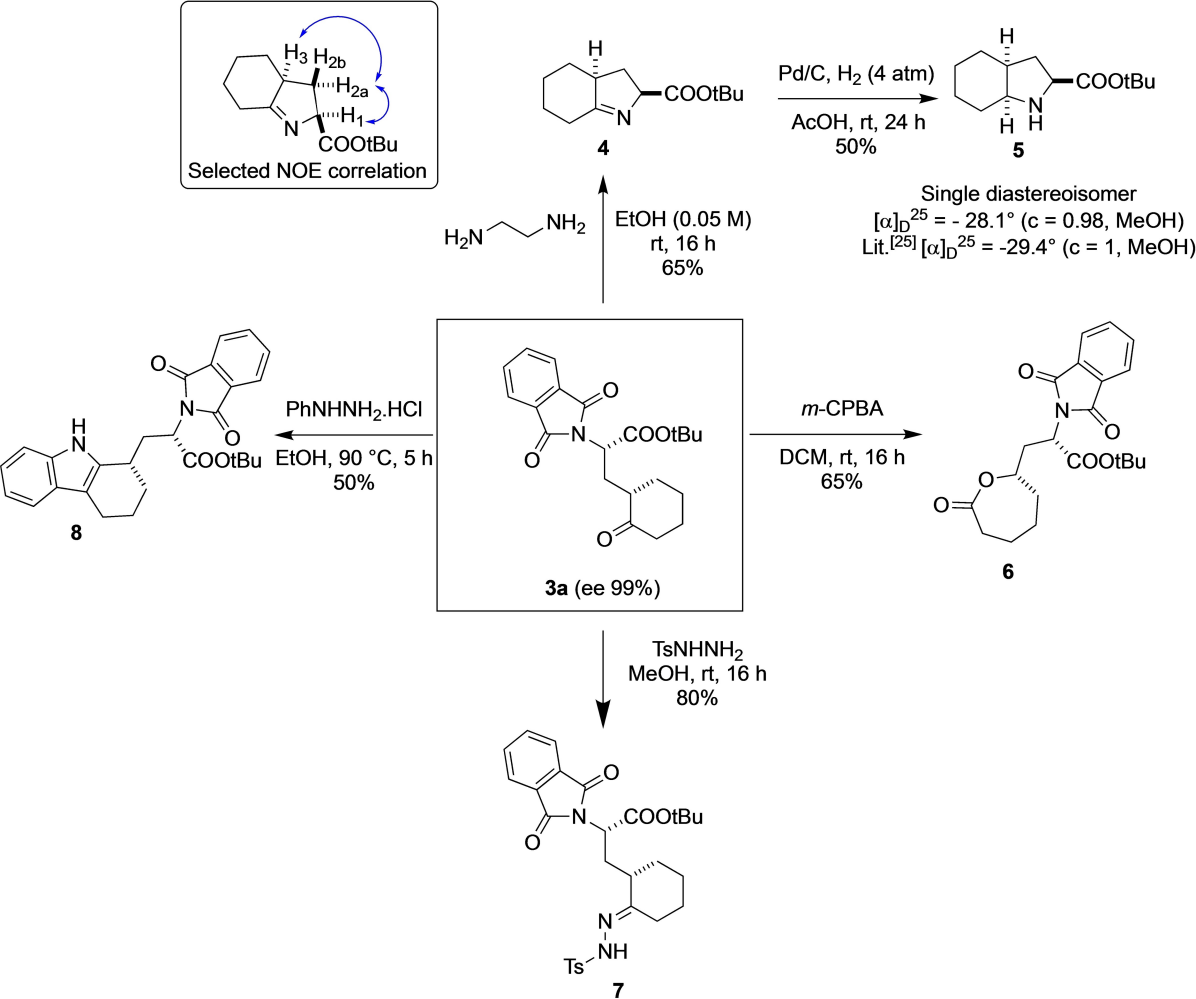
Derivatizations of product **3 a**.

[a] Unless otherwise noted, all reactions were carried out with cyclohexanone (**1 a**, 15 mg, 0.15 mmol, 1 equiv.), dehydroalanine (**2 a**, 62.6 mg, 0.23 mmol, 1.5 equiv.), and 10 mol % of catalyst in solvent (0.15 mL, 1 M) for 16 h. [b] Isolated yield. [c] Determined by ^1^H NMR analysis of the crude reaction mixture. [d] Determined by HPLC analysis using a chiral stationary phase. The absolute configuration of the major diastereomer was assigned as (*S,S*) based on the specific rotation value correlation with *tert*‐butyl (*2S,3aS,7aS*)‐octahydro‐1*H*‐indole‐2‐carboxylate (**8**) derived from the Michael addition product **3 a** (Scheme 2, see below) [e] 0.5 M was used. [f] 5 mol % of **B1** was used. [g] Performed with 2 equiv. of **1 a**.

Regarding the cyclohexanediamine‐derived catalysts, replacement of the primary amine moiety of the catalyst **A1**–**2** with the *N,N*‐dimethyl tertiary amine (Takemoto catalyst, **A3**, entry 4) completely suppressed the reactivity. This agrees with a mechanistic scenario in which the productive pathway of the reaction requires the formation of the enamine‐reactive species instead of the formation of the enolate intermediate. The same result was obtained with the substitution of the electron‐withdrawing phenyl bistrifluoromethyl group with the *tert*‐leucine residue (**A4**, entry 5), which substantially reduced both the acidity and the H‐bond donor propensity of the thiourea moiety, indicating that the H‐bonding properties of the catalyst are a key aspect for the development of a successful reaction. The removal of the phenyl bistrifluoromethyl thiourea group, **B3** and **B4** catalyst, was detrimental for the reaction outcome; whereas urea catalyst **B2** was both slightly less reactive and less stereoselective than its thiourea analogue **B1** (compare entries 6 and 7). In order to improve the efficiency of the catalysis by amine **B1**, different reaction parameters were studied. Examination of the temperature and reaction media (entries 10–17) identified 30 °C and toluene as the more effective combination (entry 6) both in terms of the yield and diastereocontrol and enantiocontrol. A second cycle of optimization using these conditions revealed that a slight excess of dehydroalanine (50 mol %), a high concentration (1 M), and catalyst loading of 10 % were optimal. These conditions provided the product **3 a** with synthetically useful results over a 16 h reaction time (entry 6, **3 a** isolated in 97 % yield, 9 : 1 *dr*, and 99 % ee) and were selected to evaluate the scope and limitations of this new reaction.

Notably, an array of carbocyclic and heterocyclic ring systems was amenable to this enamine activation approach (Table 2). In particular, tetrahydropyran‐4‐one, tetrahydrothiopyran‐4‐one, and *N*‐*tert*‐butoxycarbonyl (Boc)‐piperidin‐4‐one were successfully alkylated (**3 d**–**f**, 72–97 %, up to 10 : 1 *dr*, and 98–99 % ee), while the introduction of disubstitution at the 4‐position of cyclohexanone, making a cyclohexadione derivative, 1,4‐cyclohexanedione monoethylene acetal can be tolerated without loss in yield or selectivity (**3 g**, 97 %, 9 : 1 *dr*, 99 % ee). Moreover, we found that this new protocol could be efficiently employed with cyclopentanone, despite the formation of equal amounts of the two diastereomers (**3 b**, 1 : 1 *dr*, 99 % ee), whereas the seven‐membered ketone proved to be more difficult and failed to give the desired product (**3 c**, no reaction). The use of an unsymmetrical ketone, such as dihydro‐2*H*‐pyran‐3(4*H*)‐one, provided enantioselective alkylation with no regioselectivity, affording the respective amino acid regioisomers (**3 i** and **3 i’**) with high stereocontrol. In addition, it should be noted that this enantioselective ketone alkylation failed using both acyclic substrates (e. g., acetophenone, methyl vinyl ketone, and 1‐tetralone) and other dehydroalanine‐derived Michael acceptors (e. g., *N*‐Boc or *N*‐benzyloxycarbonyl (Cbz)), while with the use of methyl 2‐phthalimidoacrylate, the corresponding product **3 j** was obtained with almost the same results as **3 a** both in terms of the yield and the stereoselectivity of the reaction, proving that the ester moiety plays a marginal role in the stereochemical outcome of the reaction. Finally, the α‐substituted ketone 2‐methyl cyclohexanone also did not react under our standard conditions.^[22]^

**Table 2:**
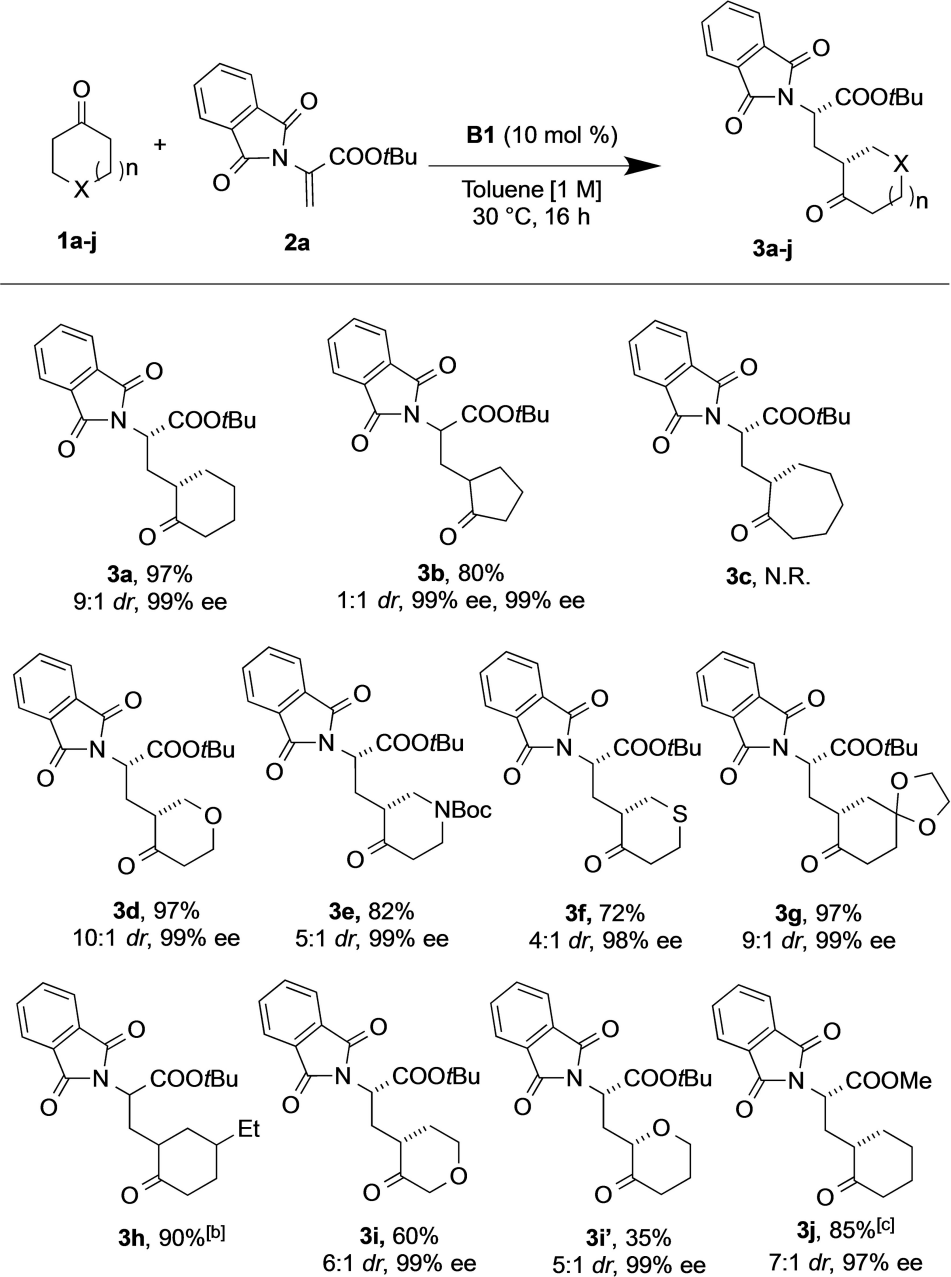
Scope of asymmetric alkylation of cyclic ketones with dehydroalanine.^[a]^

[a] All reactions were carried out with ketone **1 a**–**j** (0.15 mmol), **2 a** (1,5 equiv., 0.23 mmol), and 10 mol % of catalyst **B1** (0.015 mmol) in toluene (0.15 mL, 1 M) for 16 h at 30 °C. Isolated yield. The *dr* values were determined by ^1^H NMR analysis of the crude reaction mixtures. The ee values were determined by HPLC analysis using a chiral stationary phase. The absolute configuration of the major diastereomer of **3 a** was assigned as (*S,S*) based on the specific rotation value correlation with *tert*‐butyl (*2S,3aS,7aS*)‐octahydro‐1*H*‐indole‐2‐carboxylate (**8**) derived from the Michael addition product **3 a** (Scheme 2, see below), and those of **3 b**–**j** were assigned by analogy. [b] Mixture of inseparable diastereomeric products. [c] Using methyl 2‐phthalimidoacrylate. N. R.: no reaction.

Altogether, these results suggest a cooperative mechanism of catalysis by the thiourea and the primary amino moiety, which synergistically channel the process toward a highly stereoselective conjugated addition pathway by concomitant activation of both the nucleophilic (cyclohexanone, **1 a**) and electrophilic (dehydroalanine, **2 a**) partners. To further support this hypothesis, we investigated the catalyst **B1** (Figure S1), dehydroalanine **2 a** with the catalyst **B1** (Figure S2), and a reaction mixture concentration of 1 M in toluene at 5 min after the start of the reaction (Figure S3) by means of ESI‐MS.^[23]^ The spectra clearly showed the presence of the imine/enamine intermediate (*m/z* 564.2), the complex between the protonated catalyst **B1** and dehydroalanine **2 a** (*m/z* 757.2), and finally the anticipated enamine‐**2 a** complex (*m/z* 837.3) in which both reacting partners are brought together in close proximity to react (see Supporting Information for the details).

Considering the potential significance and accessibility of the novel and enantioenriched ketone‐based unnatural α‐amino acid frameworks, we sought to explore various synthetic transformations starting from **3 a**. First, the amino acid derivative **3 a** was prepared with a mmol‐scale reaction of **1 a** with 2‐phthalimidoacrylate (**2 a**) using catalyst **B1** in 16 h at room temperature, affording a 90 % yield, 9 : 1 *dr*, and 99 % ee. Deprotection of the adduct **3 a**, successfully obtained using ethane‐1,2‐diamine in ethanol (0.05 M), afforded the free amine, which spontaneously cyclized to form the 1‐pyrroline‐5‐carboxylic acid derivative **4**, which could be further processed by a suitable imine reduction in a cascade system to furnish stereochemically dense and constrained noncanonical proline analogue **5** (i. e., octahydroindole‐2‐carboxylic acid (Oic)), which has been used as a building block for the construction of pharmacologically relevant sequences, particularly bradykinin antagonists.^[24]^ The absolute configuration of **5** was assigned as (*S,S,S*) by comparing its specific rotation value with the reported one.^[25]^

The straightforward preparation of compound **5** allowed us to assign the relative and absolute configuration of amino acid **3 a**, considering that the cascade reaction for the preparation of **5** is a stereospecific process that depends on the stereochemistry of **3 a**. Moreover, the relative stereochemical relationship of the two stereocenters of **3 a** was further validated by Overhauser enhancement experiments (NOESY) of **4** due to the presence of an nOe correlation between H_1_ and H_2a_ as well as an nOe correlation between H_2a_ and H_3_, suggesting that the two hydrogens of the stereocenters are located on the same side of the ring (see the Supporting Information for details). Baeyer‐Villiger oxidation of **3 a** with *m*‐CPBA in DCM at room temperature furnished the seven‐membered‐ring lactone **6** as a single regioisomer in good yield. Treatment of ketone **3 a** with hydrazine derivatives was also very useful to show the versatility of the product. In particular, treatment with tosylhydrazine in methanol at room temperature afforded the corresponding *N*‐tosylhydrazones **7** as a single stereoisomer; these compounds have been extensively employed as reaction partners in various transition‐metal‐catalyzed carbene‐based cross‐coupling reactions.^[26]^ Next, ketone **3 a** was transformed into 2,3‐substituted indole‐amino acid **8** by Fischer indolization with phenylhydrazine hydrochloride in ethanol at 90 °C. Surprisingly, only one of two formally possible regioisomers, the other one would have been indolenine, was formed. This reaction hinged upon the classical reactivity of more enolizable ketones, underscoring the utility of this transformation and further demonstrating the ease with which a variety of unique heterocyclic compounds can be synthesized.

## Conclusion

In summary, considering an outstanding challenge in synthetic chemistry, we have reported a rare example of organocatalytic asymmetric α‐alkylation of ketones by a stereoselective Michael addition reaction of cyclic ketones to dehydroalanine. Key to the development of the chemistry was the use of a bifunctional primary amine‐thiourea catalyst that combines H‐bond‐directing activation and enamine catalysis. This strategy allows rapid and facile access to a series of valuable unnatural amino acids bearing a ketone moiety and 1,3‐nonadjacent stereocenters through an enantioselective nucleophilic addition/diastereoselective protonation cascade under operationally simple conditions with high stereoselectivities and yields. The dual‐activation strategy secures direct access to unreported carbonyl‐based unnatural α‐amino acids **3 a**–**j** with high stereocontrol (up to 10 : 1 *dr* and up to 99 % ee) and high yields (up to 97 %) under mild reaction conditions. The synthetic versatility of carbonyl‐containing α‐amino acids was further explored through a range of direct transformations including Fischer indolization, Bayer‐Villiger oxidation, hydrazone formation, and reductive amination directly at the carbonyl group, affording some other useful and interesting unnatural amino acids.

## Experimental Section

**General procedure for the Michael addition of ketones to** ***tert*** **‐butyl 2‐phtalimidoacrylate (2 a)**: A vial was charged with catalyst **B1** (0.015 mmol, 0.1 equiv.), *tert*‐butyl 2‐phtalimidoacrylate **2 a** (62.7 mg, 0.23 mmol, 1.5 equiv.), the appropriate ketone **1 a**–**j** (0.15 mmol, 1 equiv.), and toluene (0.15 mL). The vial was sealed and immersed in a preheated (30 °C) oil bath and stirred at this temperature for 16 h. The diastereomeric ratio (d.r.) was determined by ^1^H NMR analysis of the crude reaction mixture. The solvent was removed in vacuum and the product **3 a**–**j** was isolated by flash column chromatography on silica gel.

## Conflict of interest

The authors declare no conflict of interest.

1

## Supporting information

As a service to our authors and readers, this journal provides supporting information supplied by the authors. Such materials are peer reviewed and may be re‐organized for online delivery, but are not copy‐edited or typeset. Technical support issues arising from supporting information (other than missing files) should be addressed to the authors.

Supporting Information

## Data Availability

The data that support the findings of this study are available in the supplementary material of this article.
