# Supplementary material for: Asymmetric Alkylation of Cyclic Ketones with Dehydroalanine via H‐Bond‐Directing Enamine Catalysis: Straightforward Access to Enantiopure Unnatural α‐Amino Acids
Source: Chemistry. 2022 Aug 18;28(57):e202201994. doi: 10.1002/chem.202201994 (PMC9805190; doi:10.1002/chem.202201994)
Supplement: Supplementary file 1 — Supporting Information [file CHEM-28-0-s001.pdf]

Chemistry–A European Journal

Supporting Information

Asymmetric Alkylation of Cyclic Ketones with Dehydroalanine via H-Bond-Directing Enamine Catalysis: Straightforward Access to Enantiopure Unnatural α-Amino Acids

Michele Retini, Silvia Bartolucci, Francesca Bartoccini, and Giovanni Piersanti*

Table of Contents

1. General Informations S3

2. Starting Materials S3

3. Catalysts S3

4. General procedure for the Michael addition of ketones to *tert*-butyl 2-phtalimidoacrylate S4

5. Characterization data of ketone-based unnatural amino acids (3a-j) S4

6. Synthetic Procedures and Spectral Data for product derivatizations S10

7. Intermediate Characterization: ESI/MS Studies of the S13

8. References S17

9. Copies of ^{1}H NMR and ^{13}C NMR spectra S18

10. Copies of chiral HPLC chromatogram S49

1. General information

All reactions were run in air unless otherwise noted. Column chromatography purifications were performed in flash chromatography conditions using Merck 230-400 Mesh silica gel. Analytical thin layer chromatography (TLC) was carried out on Merck silica gel plates (Silica Gel 60 F_{254}), that were visualized by exposure to ultraviolet light and an aqueous solution of $KMnO_4$. ^{1}H NMR and ^{13}C NMR spectra were recorded on a Bruker Avance 400 spectrometer, using $CDCl_3$, $(CD_3)_2CO$, CD_3OD as solvent. Chemical shifts (δ scale) are reported in parts per million (ppm) relative to the central peak of the solvent. Coupling constants (*J* values) are given in hertz (Hz). The diastereomeric ratio was determined by ^{1}H NMR analysis of the crude reaction mixture on the Michael addition products **3**. Enantiomeric excesses were determined by HPLC analyses on chiral stationary phase performed on an Agilent 1260-Infinity II series instrumentation. Daicel Chiralpak AD-H or OD-H columns with hexane/*i*-PrOH as the eluent were used. HPLC traces were compared to racemic samples prepared using benzyl amine as the catalyst (40 mol %) following the same general procedure described in the experimental section but running the reaction at 90 °C for 48 h, allowing the preparation of the racemic compounds with low yields and as diastereoisomers mixture. The absolute configuration of the major diastereomer of **3a** was assigned as (*S*,*S*) based on the specific rotation value correlation with *tert*-butyl (2*S*,3*aS*,7*aS*)-octahydro-1*H*-indole-2-carboxylate (**8**) derived from the Michael addition product **3a** (see the corresponding section) and those of **3b-j** were assigned by analog.
Optical rotation analysis was performed with a polarimeter using a sodium lamp (λ 589 nm, D-line); $[\alpha]_D^{25}$ values are reported in 10^{-1} deg cm^2 g^{-1}; concentration (c) is in g for 100 mL. ESI-MS spectra were taken on a Waters Micromass ZQ instrument.

2. Starting materials

All ketones were purchased from best-known commercial suppliers and used without further purification. *Tert*-butyl 2-phtalimidoacrylate (**2a**) and methy- 2-phtalimidoacrylate were synthesized according to the literature procedure.[1]

3. Catalysts

Bifunctional thiourea **A1** and **A2** are commercially available, catalyst 1-(3,5-bis(trifluoromethyl)phenyl)-3-((1*R*,2*R*)-2-(dimethylamino)cyclohexyl)thiourea (**A3**), (*S*)-2-[[(1*R*,2*R*)-2-Aminocyclohexyl] thioureido]-*N*-benzyl-*N*,3,3-trimethylbutanamide (**A4**), (1*R*,2*R*)-1,2-diphenylethane-1,2-diamine (**B3**) and *N*-((1*R*,2*R*)-2-amino-1,2-diphenylethyl)-4-methylbenzenesulfonamide (**B4**) are commercially available. The bifunctional primary amine-thiourea catalyst **B1** was prepared according to literature[2] procedure and 1-((1*R*,2*R*)-2-amino-1,2-diphenylethyl)-3-(3,5-bis(trifluoromethyl)phenyl)urea (**B2**) was prepared according to literature procedure.[3]

4. General procedure for the Michael addition of ketones to *tert*-butyl 2-phtalimidoacrylate (2a)

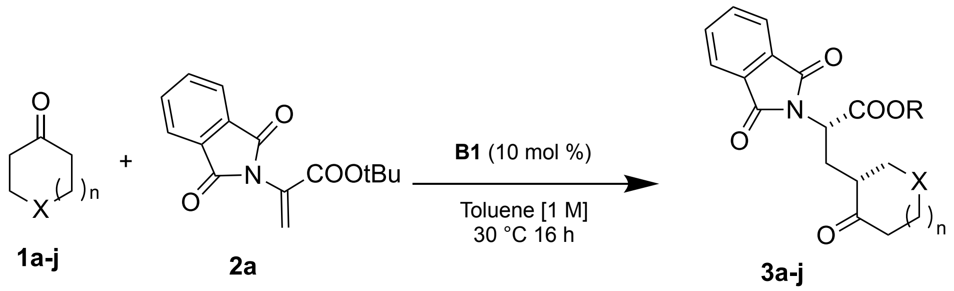

1a-j **2a** **3a-j**

A vial was charged with catalyst **B1** (0.015 mmol, 0.1 equiv), *tert*-butyl 2-phtalimidoacrylate **2a** (62.7 mg, 0.23 mmol, 1.5 equiv), the appropriate ketone **1a-j** (0.15 mmol, 1 equiv), and toluene (0.15 mL). The vial was sealed and immersed in a preheated (30 °C) oil bath and stirred at this temperature for 16 h. The diastereomeric ratio (d.r.) was determined by ^{1}H NMR analysis of the crude reaction mixture. The solvent was removed in vacuum and the product **3a-j** was isolated by flash column chromatography on silica gel.

5. Characterization data of ketone-based unnatural amino acids (3a-j)

(*S*)-*tert*-butyl 2-(1,3-dioxoisoindolin-2-yl)-3-((*S*)-2-oxocyclohexyl)propanoate (3a)

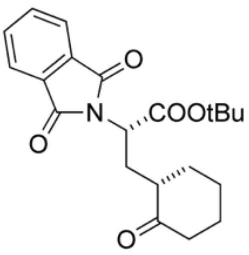

3a

The reaction was carried out following the general procedure to furnish the crude products as a 9:1 mixture of diastereoisomers; d.r. determined by integration of ^{1}H NMR signals: δ_{major} 5.04 ppm (dd), δ_{minor} 4.81 ppm (dd). The title compound **3a** was isolated as a single diastereoisomer by flash column chromatography on silica gel (gradient from cyclohexane/ethyl acetate 9:1 to 7:3) in 97% yield (55 mg, pale-yellow oil). The enantiomeric excess was determined to be 99% by HPLC analysis (chiral column AD-H; mobile phase hexane/*i*-PrOH 90:10, flux 1.0 mL min^{-1}, λ = 220 nm), tr (minor) = 13.1 min, tr (major) = 16.6 min.

The reaction was also carried out on 2 mmol scale to furnish the crude products as a 9:1 mixture of diastereoisomers. The title compound **3a** was isolated as a single diastereoisomer by flash column chromatography on silica gel (gradient from cyclohexane/ethyl acetate 9:1 to 7:3) in 90% yield (668 mg, pale-yellow oil).

^{1}H NMR ($CDCl_3$, 400 MHz): δ 1.41 (s, 9H), 1.45-1.49 (m, 1H), 1.57-1.64 (m, 2H), 1.79-1.82 (m, 1H), 1.92-2.05 (m, 3H), 2.20-2.38 (m, 3H), 2.62-2.69 (m, 1H), 5.02 (dd, *J* = 10.8, 4.3 Hz, 1H), 7.71-7.76 (m, 2H), 7.83-7.88 (m, 2H).

^{13}C NMR ($CDCl_3$, 100 MHz): δ 25.3, 27.7, 27.9, 28.2, 29.3, 35.1, 42.2, 47.1, 51.5, 82.4, 123.4, 131.9, 134.1, 167.9, 168.1, 211.9.

HRMS (ESI-TOF) m/z calcd. for $C_{21}H_{26}NO_5$ $[M+H]^+$: 372.1805; found 372.1811.
$[\alpha]_D^{25}$= -17.8 (c = 1.043, $CHCl_3$).

(*S*)-*tert*-butyl 2-(1,3-dioxoisoindolin-2-yl)-3-((*S*)-2-oxocyclopentyl)propanoate (3b)

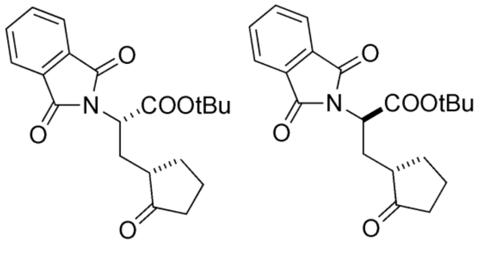

3b **anti-3b**

The reaction was carried out following the general procedure to furnish the crude products as a 1:1 mixture of diastereoisomers; d.r. determined by integration of ^{1}H NMR signals: δ_{major} 5.06 ppm (dd), δ_{minor} 4.83 ppm (dd). The title compound **3b** was isolated as 1:1 diastereomeric mixture by flash column chromatography on silica gel (gradient from cyclohexane/ethyl acetate 9:1 to 75:25) in 77% overall yield (42 mg, colorless oil).The enantiomeric excess was of both diastereoisomers was determined to be 99% by HPLC analysis (chiral column OD-H; mobile phase hexane/*i*-PrOH 95:5, flux 1.0 mL min^{-1}, λ = 220 nm), tr_{d1} (major) = 23.3 min, tr_{d1} (minor) = 28.3 min, tr_{d2} (major) = 26.3 min, tr_{d2} (minor) = 30.3 min.

^{1}H NMR ($CDCl_3$, 400 MHz, 1:1 mixture of diastereoisomers **3b** and **anti-3b**): δ 1.42 (s, $9H_{3b}$, $9H_{anti\text{-}3b}$), 1.46-1.80 (m, $2H_{3b}$, $2H_{anti\text{-}3b}$), 1.93-2.17 (m, $4H_{3b}$, $4H_{anti\text{-}3b}$), 2.21-2.38 (m, $2H_{3b}$, $2H_{anti\text{-}3b}$), 2.61-2.67 (m, 1H), 2.72-2.79 (m, 1H), 4.83 (dd, *J* = 11.8, 4.0Hz, $1H_{anti\text{-}3b}$), 5.06 (dd, *J* = 10.5, 4.5Hz, $1H_{3b}$), 7.72-7.77 (m, $2H_{3b}$, $2H_{anti\text{-}3b}$), 7.84-7.90 (m, $2H_{3b}$, $2H_{anti\text{-}3b}$).

^{13}C NMR ($CDCl_3$, 100 MHz): δ 20.5, 20.6, 27.8, 27.9, 28.9, 29.3, 29.7, 29.8, 37.5, 37.8, 46.5, 46.6, 51.5, 51.8, 82.7, 82.8, 123.47, 123.49, 131.84, 131.85, 134.15, 167.7, 167.8, 167.9.

HRMS (ESI-TOF) m/z calcd. for $C_{20}H_{24}NO_5$ $[M+H]^+$: 358.1649; found 358.1654.
$[\alpha]_D^{25}$= -19.3 (c = 1.03 g/ml, $CHCl_3$).

(*S*)-*tert*-butyl 2-(1,3-dioxoisoindolin-2-yl)-3-((*S*)-4-oxotetrahydro-2*H*-pyran-3-yl)propanoate (3d)

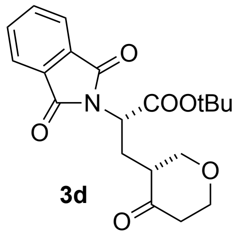

3d

The reaction was carried out following the general procedure to furnish the crude products as a 10:1 mixture of diastereoisomers; d.r. determined by integration of ^{1}H NMR signals: δ_{major} 5.06 ppm (dd), δ_{minor} 4.77 ppm (dd). The title compound **3d** was isolated as a single diastereoisomer by flash column chromatography on silica gel (gradient from cyclohexane/ethyl acetate 9:1 to 8:2) in 97% yield (54 mg, colorless oil). The enantiomeric excess determined to be

99% by HPLC analysis (chiral column AD-H; mobile phase hexane/*i*-PrOH 90:10, flux 1.0 mL min^{-1}, λ = 220 nm), tr (minor) = 21.9 min, tr (major) = 23.4 min.

^{1}H NMR ($CDCl_3$, 400 MHz): δ 1.42 (s, 9H), 1.88-1.95 (m, 1H), 2.35-2.40 (m, 1H), 2.52-2.65 (m, 3H), 3.36 (t, *J* = 10.8 Hz, 1H), 3.63-3.69 (m, 1H), 4.07-4.12 (m, 1H), 4.18-4.24 (m, 1H), 5.04 (dd, *J* = 10.9, 3.9 Hz, 1H), 7.73-7.77 (m, 2H), 7.84-7.88 (m, 2H).

^{13}C NMR ($CDCl_3$, 100 MHz): δ 24.7, 27.9, 42.8, 48.1, 51.3, 68.9, 73.0, 82.7, 123.5, 131.8, 134.3, 167.7, 167.9, 207.2.

HRMS (ESI-TOF) m/z calcd. for $C_{20}H_{23}NO_6Na$ [M+Na]$^+$: 396.1418; found 396.1440.

$[\alpha]_D^{25}$= -32.6 (c = 0.767, $CHCl_3$).

(*S*)-*tert*-butyl 3-((*S*)-3-*tert*-butoxy-2-(1,3-dioxoisoindolin-2-yl)-3-oxopropyl)-4-oxopiperidine-1-carboxylate (3e)

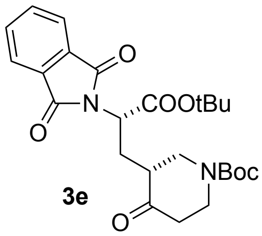

3e

The reaction was carried out following the general procedure to furnish the crude products as a 5:1 mixture of diastereoisomers; d.r. determined by integration of ^{1}H NMR signals: δ_{major} 5.03 ppm (dd), δ_{minor} 4.88 ppm (dd). The title compound **3e** was isolated as a single diastereoisomer by flash column chromatography on silica gel (gradient from cyclohexane/ethyl acetate 9:1 to 8:2) in 82% yield (59.3 mg, colorless oil). The enantiomeric excess determined to be 99% by HPLC analysis (chiral column AD-H; mobile phase hexane/*i*-PrOH 90:10, flux 1.0 mL min^{-1}, λ = 220 nm), tr (minor) = 17.5 min, tr (major) = 23.7 min.

^{1}H NMR ($CDCl_3$, 400 MHz): δ 1.39 (s, 9H), 1.41 (s, 9H), 1.93-2.06 (m, 1H), 2.35-2.50 (m, 3H), 2.59-2.66 (m, 1H), 2.78-2.94 (m, 1H), 3.13-3.20 (m, 1H), 4.16-4.22 (m, 2H), 5.01 (dd, *J* = 10.7, 3.9 Hz, 1H), 7.72-7.77 (m, 2H), 7.84-7.88 (m, 2H).

^{13}C NMR ($CDCl_3$, 100 MHz): δ 27.8, 28.2, 41.1, 43.9, 46.6, 47.7, 49.2, 51.2, 80.5, 82.7, 123.5, 131.8, 134.2, 154.3, 167.6, 167.8, 208.4.

HRMS (ESI-TOF) m/z calcd. for $C_{25}H_{33}N_2O_7$ [M+H]$^+$: 473.2282; found 473.2277.

$[\alpha]_D^{25}$= -36 (c = 0.86, $CHCl_3$).

(*S*)-*tert*-butyl 2-(1,3-dioxoisoindolin-2-yl)-3-((*S*)-4-oxotetrahydro-2*H*-thiopyran-3-yl)propanoate (3f)

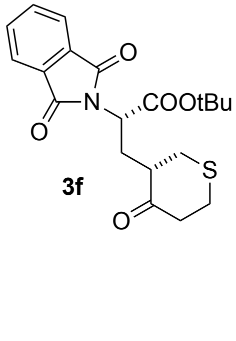

3f

The reaction was carried out following the general procedure to furnish the crude products as a 6:1 mixture of diastereoisomers; d.r. determined by integration of ^{1}H NMR signals: δ_{major} 4.96 ppm (dd), δ_{minor} 4.78 ppm (dd). The title compound **3f** was isolated as a single diastereoisomer by flash column chromatography on silica gel (gradient from cyclohexane/ethyl acetate 95:5 to 8:2) in 42% yield (25 mg, pale-yellow solid).The enantiomeric excess determined to be 99% by HPLC analysis (chiral column AD-H; mobile phase hexane/*i*-PrOH 90:10, flux 1.0 mL min^{-1}, λ = 220 nm), tr (minor) = 24.2 min, tr (major) = 28.5 min.

^{1}H NMR ($CDCl_3$, 400 MHz): δ 1.42 (s, 9H), 2.01-2.08 (m, 1H), 2.61-2.79 (m, 5H), 2.82-3.02 (m, 3H), 4.94 (dd, *J* = 11.0, 4.0 Hz, 1H), 7.74-7.78 (m, 2H), 7.84-7.89 (m, 2H).

^{13}C NMR ($CDCl_3$, 100 MHz): δ 27.9, 29.2, 31.5, 37.0, 44.6, 49.8, 51.1, 82.7, 123.5, 131.8, 134.2, 167.7, 167.9, 209.0.

HRMS (ESI-TOF) m/z calcd. for $C_{20}H_{24}NO_5S$ $[M+H]^+$: 390.1370; found 390.1378.

$[\alpha]_D^{25}$= -52.3 (c = 0.874, $CHCl_3$).

(*S*)-*tert*-butyl 2-(1,3-dioxoisoindolin-2-yl)-3-((*S*)-8-oxo-1,4-dioxaspiro[4.5]decan-7-yl)propanoate (3g)

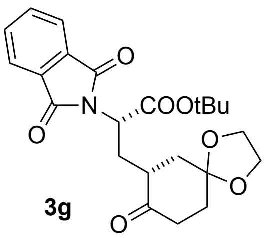

3g

The reaction was carried out following the general procedure to furnish the crude products as a 9:1 mixture of diastereoisomers; d.r. determined by integration of ^{1}H NMR signals: δ_{major} 5.08 ppm (dd), δ_{minor} 4.77 ppm (dd). The title compound **3g** was isolated as a single diastereoisomer by flash column chromatography on silica gel (gradient from cyclohexane/ethyl acetate 95:5 to 8:2) in 42% yield (25 mg, pale-yellow solid). The enantiomeric excess determined to be >99% by HPLC analysis (chiral column AD-H; mobile phase hexane/*i*-PrOH 90:10, flux 1.0 mL min^{-1}, λ = 220 nm), tr (minor) = 27.3 min, tr (major) = 46.0 min.

^{1}H NMR ($CDCl_3$, 400 MHz): δ 1.42 (s, 9H), 1.77-1.84 (m, 1H), 1.93-2.03 (m, 4H), 2.32-2.37 (m, 1H), 2.56-2.67 (m, 3H), 3.93-3.98 (m, 4H), 5.07 (dd, *J* = 10.9, 3.8 Hz, 1H), 7.72-7.77 (m, 2H), 7.83-7.88 (m, 2H).

^{13}C NMR ($CDCl_3$, 100 MHz): δ 27.9, 29.0, 35.0, 38.2, 41.6, 42.7, 51.3, 64.6, 64.7, 82.5, 107.1, 123.5, 131.9, 134.1, 167.97, 167.99, 210.6.

HRMS (ESI-TOF) m/z calcd. for $C_{23}H_{28}NO_7$ $[M+H]^+$: 430.1860; found 430.1845

$[\alpha]_D^{25}$= -13.2 (c =1.024, $CHCl_3$).

(2*S*)-*tert*-butyl 2-(1,3-dioxoisoindolin-2-yl)-3-((1*S*)-5-ethyl-2-oxocyclohexyl)propanoate (3h)

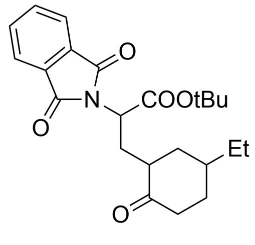

3h

The reaction was carried out following the general procedure to furnish the crude products as a mixture of three diastereoisomers (2:2:1); d.r. determined by integration of ^{1}H NMR signals: δ 5.06 ppm (dd), δ 4.88 ppm (dd) and δ 4.80 ppm (dd). The title compound **3h** was isolated as a mixture of three diastereoisomers (2:3:1) by flash column chromatography on silica gel (gradient from cyclohexane/ethyl acetate 95:5 to 8:2) in 90% yield (55 mg, pale-white solid).

^{1}H NMR ($CDCl_3$, 400 MHz, 2:3:1 mixture of diastereisomers): δ 0.85-0.89 (m, 4.99H), 0.91-0.96 (m, 0.99H) 1.41-1.42 (s, 17.91H), 1.66-2.42 (m, 17.91H), 2.60-2.71 (m, 2H), 4.80 (dd, *J* = 10.0, 5.3 Hz, 0.33H), 4.88 (dd, *J* = 10.5, 4.5 Hz, 1H), 5.06 (dd, *J* = 10.8, 4.2 Hz, 0.66H), 7.73-7.76 (m, 3.98H), 7.85-7.88 (m, 3.98H).

^{13}C NMR ($CDCl_3$, 100 MHz, 2:3:1 mixture of diastereisomers): δ 11.70, 11.78, 12.0, 25.9, 26.2, 26.9, 27.84, 27.86, 27.87, 28.5, 28.6, 29.0, 29.2, 29.7, 29.9, 30.2, 31.4, 31.6, 33.4, 33.5, 33.8, 33.9, 37.1, 37.8, 37.9, 38.1, 38.5, 38.6, 39.6, 40.9, 41.36, 41.42, 43.9, 45.0, 45.9, 47.0, 50.9, 51.5, 51.6, 82.4, 82.57, 82.59, 82.7, 123.44, 123.46, 131.84, 131.88, 134.05, 134.08, 134.11, 167.75, 167.87, 167.92, 168.02, 168.11, 168.4, 211.1, 212.3, 213.0.

HRMS (ESI-TOF) m/z calcd. for $C_{23}H_{30}NO_5$ $[M+H]^+$: 400.2118; found 400.2124.

$[\alpha]_D^{25}$= -18.1 (c = 0.965, $CHCl_3$).

(*S*)-*tert*-butyl 2-(1,3-dioxoisoindolin-2-yl)-3-((*S*)-3-oxotetrahydro-2*H*-pyran-4-yl)propanoate (3i)

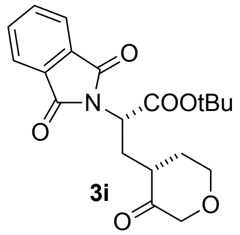

3i

The reaction was carried out following the general procedure to furnish the crude products as a 3:1 mixture of diastereoisomers; d.r. determined by integration of ^{1}H NMR signals: δ_{major} 5.11 ppm (dd), δ_{minor} 4.68 ppm (dd). The title compound **3i** was isolated as a single diastereoisomer by flash column chromatography on silica gel (gradient from cyclohexane/ethyl acetate 95:5 to 8:2) in 60% yield (33 mg, pale-yellow solid). The enantiomeric excess determined to be 99% by HPLC analysis (chiral column AD-H; mobile phase hexane/*i*-PrOH 90:10, flux 1.0 mL min^{-1}, λ = 220 nm), tr (minor) = 16.9 min, tr (major) = 22.4 min.

^{1}H NMR ($CDCl_3$, 400 MHz): 1.42 (s, 9H), 1.81-1.90 (m, 1H), 2.10-2.20 (m, 2H), 2.52-2.60 (m, 1H), 2.70-2.77 (m, 1H), 3.74-3.81 (m, 1H), 3.87-3.94 (m, 1H), 3.95-4.03 (m, 2H), 5.10 (dd, *J* = 10.3, 4.7 Hz, 1H), 7.73-7.77 (m, 2H), 7.84-7.88 (m, 2H).

^{13}C NMR ($CDCl_3$, 100 MHz): δ 26.0, 27.9, 29.1, 37.6, 49.9, 65.9, 81.3, 82.6, 123.3, 132.1, 134.0, 167.6, 167.8, 207.1.

HRMS (ESI-TOF) m/z calcd. for $C_{20}H_{24}NO_6$ $[M+H]^+$: 374.1598; found 374.1609.

$[\alpha]_D^{25}$= -40.1 (c = 0.82, $CHCl_3$).

(*S*)-*tert*-butyl 2-(1,3-dioxoisoindolin-2-yl)-3-((*S*)-3-oxotetrahydro-2*H*-pyran-2-yl)propanoate (3i')

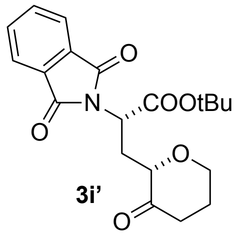

3i'

The reaction was carried out following the general procedure to furnish the crude products as a 3:1 mixture of diastereoisomers; d.r. determined by integration of ^{1}H NMR signals: δ_{major} 5.03 ppm (dd), δ_{minor} 4.83 ppm (dd).The title compound **3i'** was isolated as a single diastereoisomer by flash column chromatography on silica gel (gradient from cyclohexane/ethyl acetate 95:5 to 8:2) in 35% yield (17 mg, pale-yellow solid).The enantiomeric excess determined to be 99% by HPLC analysis (chiral column AD-H; mobile phase hexane/*i*-PrOH 90:10, flux 1.0 mL min^{-1}, λ = 220 nm), tr (minor) = 23.9 min, tr (major) = 25.3 min.

^{1}H NMR ($CDCl_3$, 400 MHz): 1.43 (s, 9H), 1.92-2.00 (m, 1H), 2.02-2.13 (m, 1H), 2.26-2.42 (m, 2H), 2.46-2.52 (m, 1H), 2.81-2.87 (m, 1H), 3.57-3.63 (m, 1H), 3.82-3.87 (m, 1H), 4.01-4.04 (m, 1H), 5.02 (dd, *J* = 9.0, 5.3 Hz, 1H), 7.70-7.76 (m, 2H), 7.83-7.88 (m, 2H).

^{13}C NMR ($CDCl_3$, 100 MHz): δ 26.0, 27.9, 29.1, 37.6, 49.9, 65.9, 81.3, 82.6, 123.3, 132.1, 134.0, 167.6, 167.8, 207.1.

HRMS (ESI-TOF) m/z calcd. for $C_{20}H_{24}NO_6$ $[M+H]^+$: 374.1598; found 374.1607.

$[\alpha]_D^{25}$= -56 (c = 0.98, $CHCl_3$).

(*S*)-methyl 2-(1,3-dioxoisoindolin-2-yl)-3-((*S*)-2-oxocyclohexyl)propanoate (3j)

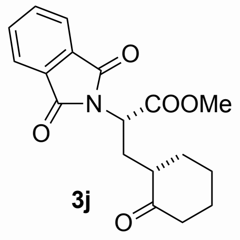

3j

The reaction was carried out following the general procedure, using methy- 2-phtalimidoacrylate, to furnish the crude products as a 6:1 mixture of diastereoisomers; d.r. determined by integration of ^{1}H NMR signals: δ_{major} 5.15 ppm (dd), δ_{minor} 4.91 ppm (dd). The title compound **3j** was isolated as a single diastereoisomer by flash column chromatography on silica gel (gradient from cyclohexane/ethyl acetate 9:1 to 8:2) in 75% yield (38 mg, white solid).The enantiomeric excess determined to be 97% by HPLC analysis (chiral column AD-H; mobile phase hexane/*i*-PrOH 90:10, flux 1.0 mL min^{-1}, λ = 220 nm), tr (minor) = 29.1 min, tr (major) = 31.4 min.

^{1}H NMR ($CDCl_3$, 400 MHz): δ 1.39-1-49 (m, 1H), 1.58-1.63 (m, 2H), 1.80-1.82 (m, 1H), 1.95-2.07 (m, 3H), 2.21-2.30 (m, 2H), 2.37-2.40 (m, 1H), 2.66-2.73 (m, 1H), 3.73 (s, 3H), 5.13 (dd, *J* = 10.9, 4 Hz, 1H), 7.74-7.77 (m, 2H), 7.85-7.87 (m, 2H).

^{13}C NMR ($CDCl_3$, 100 MHz): δ 25.3, 28.2, 29.3, 35.1, 42.2, 46.9, 50.6, 52.7, 123.6, 131.8, 134.2, 167.8, 169.7, 211.9.

HRMS (ESI-TOF) m/z calcd. for $C_{18}H_{20}NO_5$ $[M+H]^+$: 330.1336; found 330.1328.
$[\alpha]_D^{25}$= -48.2 (c = 0.786, $CHCl_3$).

6. Synthetic Procedures and Spectral Data for product derivatizations

(2*S*,3*aS*)-*tert*-butyl 3,3a,4,5,6,7-hexahydro-2*H*-indole-2-carboxylate (4):

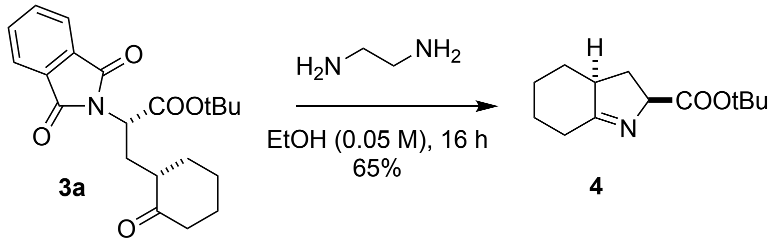

To a solution of compound **3a** (167 mg, 0.45 mmol, 1 equiv) in ethanol (10 mL) was added diethylene diamine (90 µL, 1.35 mmol, 3.0 equiv), and the reaction was stirred overnight at room temperature for 16 h. The mixture was concentrated and purified by flash column chromatography (dichloromethane/ethyl acetate 1:1) to give **4** as a colorless oil (66 mg, 0.29 mmol, 65%).
^{1}H NMR (($CD_3)_2CO$, 400 MHz): δ 1.03-1.13 (m, 1H), 1.31-1.40 (m, 1H), 1.42 (s, 9H), 1.48-1.53 (m, 1H), 1.59-1.66 (m, 1H), 1.75-1.80 (m, 1H), 1.95-2.01 (m, 1H), 2.10-2.24 (m, 3H), 2.55-2.60 (m, 1H), 2.69-2.83 (m, 1H), 4.47-4.51 (m, 1H).
^{13}C NMR (($CD_3)_2CO$, 100 MHz): δ 25.0, 26.6, 27.2, 27.3, 31.4, 33.5, 34.5, 73.5, 79.7, 171.9, 181.3.
HRMS (ESI-TOF) m/z calcd. for $C_{13}H_{22}NO_2$ $[M+H]^+$: 224.1645; found 224.1656.

The relative stereochemical relationship of the two stereocenters of **4** was assigned by Overhauser enhancement experiments NOESY, see the NMR spectra section while the absolute configuration was assigned as (*S*,*S*) based on the specific rotation value correlation with *tert*-butyl (2*S*,3*aS*,7*aS*)-octahydro-1*H*-indole-2-carboxylate (**5**) derived from **4**, see below.

(2*S*,3*aS*,7*aS*)-*tert*-butyl octahydro-1*H*-indole-2-carboxylate (5):

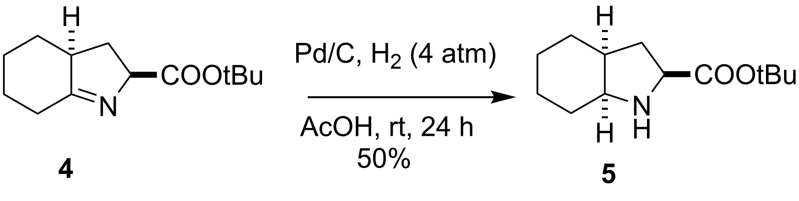

A 100 mL stainless-steel autoclave was charged, in the presence of air, with 10 wt % Pd/C (14.3 mg, 0.013 mmol, 0.10 equiv), 2.1 mL of acetic acid and compound **4** (30 mg, 0.13 mmol, 1 equiv). The autoclave was pressurized with H_2 (4 atm) and the mixture was stirred at room temperature per 24 h. The autoclave was degassed and the reaction mixture was filtered through a plug of Celite and the washed with a saturated solution potassium bicarbonate. The organic phase was dried over Na_2SO_4, concentrated and the residue was purified by flash chromatography (gradient from dichloromethane/methanol 10:0 to 95:5) to give **5** as a colorless oil (15 mg, 0.066 mmol, 50%).

^{1}H NMR (CD_3OD, 400 MHz): δ 1.26-1.31 (m, 3H), 1.44-1.48 (m, 2H), 1.49 (s, 9H), 1.52-1.55 (m, 2H), 1.69-1.75 (m, 3H), 2.02-2.09 (m, 1H), 2.20-2.28 (m, 1H), 3.08-3.13 (m, 1H), 3.66-3.70 (dd, *J* = 10.3, 5.7 Hz, 1H).
^{13}C NMR (CD_3OD, 100 MHz): δ 22.4, 24.5, 28.0, 28.1, 28.2, 37.0, 39.2, 59.5, 60.0, 83.0, 175.5.
HRMS (ESI-TOF) m/z calcd. for $C_{13}H_{24}NO_2$ $[M+H]^+$: 226.1802; found 226.1809.
$[\alpha]_D^{25}$= -28.16 (c = 0.98, CH_3OH). Lit. -29.4 (c = 1.0, CH_3OH).[4] The absolute configuration of **5** was assigned as (*S*,*S*,*S*) by comparing the specific rotation values with the reported ones.[4]

(*S*)-*tert*-butyl 2-(1,3-dioxoisoindolin-2-yl)-3-((*S*)-7-oxooxepan-2-yl)propanoate (6):

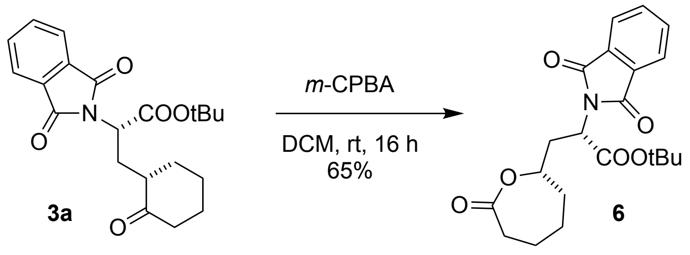

Meta-chloroperbenzoic acid (69 mg, 0.4 mmol, 2 equiv) was added to a 0.2 M stirred solution of **3a** in dichloromethane at 25 °C under N_2. After 16 h, a solution of saturated $Na_2S_2O_3$ was added and the aqueous layers extracted with CH_2Cl_2 (3x10 mL). The combined organic layers were dried over Na_2SO_4, concentrated under reduced pressure and the residue purified by flash chromatography (gradient from cyclohexane/ethyl acetate 9:1 to 6:4) to give **6** as a pale-withe solid (54.3 mg, 0.14 mmol, 70%).
^{1}H NMR ($CDCl_3$, 400 MHz): δ 1.42 (s, 9H), 1.53-1.64 (m, 2H), 1.69-1.78 (m, 1H), 1.84-1.93 (m, 3H), 2.36-2.47 (m, 2H), 2.55-2.64 (m, 2H), 4.09-4.14 (m, 1H), 5.10 (dd, *J* = 11.1, 3.3 Hz, 1H), 7.73-7.78 (m, 2H), 7.86-7.90 (m, 2H).
^{13}C NMR ($CDCl_3$, 100 MHz): δ 22.8, 27.8, 28.0, 34.6, 35.5, 50.0, 76.4, 82.8, 123.6, 131.9, 134.2, 167.6, 167.8, 174.6.
HRMS (ESI-TOF) m/z calcd. for $C_{21}H_{26}NO_6$ $[M+H]^+$: 388.1755; found 388.1760.
$[\alpha]_D^{25}$= -66.0 (c = 0.615, $CHCl_3$).

(*S*)-*tert*-butyl 2-(1,3-dioxoisoindolin-2-yl)-3-((*S,E*)-2-(2-tosylhydrazono)cyclohexyl)propanoate (7):

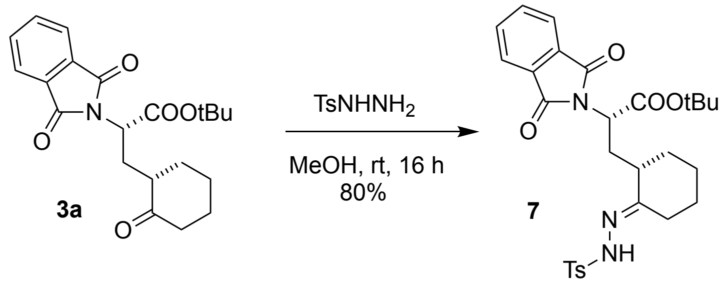

To a solution of **3a** (65 mg, 0.175 mmol, 1 equiv) in methanol (0.74 mL), was added *p*-toluenesulfonyl hydrazide (39 mg, 0.21 mmol, 1.2 equiv), and the mixture was stirred at room temperature for 16 h. The mixture was concentrated and purified by flash column chromatography (gradient from cyclohexane/ethyl acetate 9:1 to 6:4) to give **7** as a pale-white solid (76 mg, 0.14 mmol, 80%).
^{1}H NMR (CD_3OD, 400 MHz): δ 1.20-1.41 (m, 4H), 1.45 (s, 9H), 1.63-1.78 (m, 4H), 1.87-2.02 (m, 2H), 2.41 (s, 3H), 2.63-2.70 (m, 1H), 2.76-2.80 (m, 1H), 4.93 (dd, *J* = 11.3, 3.7 Hz, 1H), 7.34-7.37 (m, 2H), 7.81-7.87 (m, 6H).

^{13}C NMR (CD_3OD, 100 MHz): δ 18.7, 23.5, 25.0, 25.1, 25.4, 25.8, 28.7, 33.8, 39.4, 49.8, 80.5, 121.4, 126.4, 127.6, 130.2, 132.7, 134.5, 142.0, 161.2, 166.3, 167.4.

HRMS (ESI-TOF) m/z calcd. for $C_{28}H_{34}N_3O_6S$ $[M+H]^+$: 540.2163; found 540.2174.

$[\alpha]_D^{25}$= -94.7 (c = 1.05, CH_3OH).

(*S*)-*tert*-butyl 2-(1,3-dioxoisoindolin-2-yl)-3-((*S*)-2,3,4,9-tetrahydro-1*H*-carbazol-1-yl)propanoate (8):

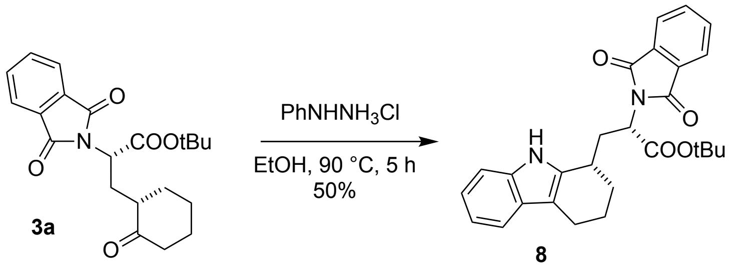

To a solution of **3a** (74.3 mg, 0.2 mmol, 1 equiv) in ethanol (1 mL), phenylhydrazine hydrochloride (34.7 mg, 0.24 mmol, 1.2 equiv) was added and the mixture was stirred at 90 °C for 5 h. The reaction mixture was allowed to cool to room temperature, the solvent was removed under reduced pressure and the residue purified by flash chromatography (gradient from cyclohexane/ethyl acetate 9:1 to 6:4) to give **8** as a pale-yellow solid (44.4 mg, 0.1 mmol, 50%).

^1H NMR ($CDCl_3$, 400 MHz): δ 1.03-1.10 (m, 1H), 1.35 (s, 9H), 1.39-1.51 (m, 1H), 1.67-1.72 (m, 1H), 1.85-1.98 (m, 1H), 2.24-2.32 (m, 2H), 2.65-2.73 (m, 1H), 2.99-3.15 (m, 3H), 4.12 (dd, *J* = 11.2, 3.0 Hz, 1H), 6.59 (td, *J* = 7.4, 0.9 Hz, 1H), 6.74 (td, *J* = 7.6, 1.2 Hz, 1H), 7.01-7.03 (m, 1H), 7.33-7.35 (m, 1H), 7.57-7.61 (m, 4H).

^{13}C NMR ($CDCl_3$, 100 MHz): δ 21.2, 27.7, 29.4, 30.4, 31.4, 39.5, 49.4, 56.6, 82.8, 120.5, 121.2, 122.9, 124.5, 127.0, 131.4, 133.5, 143.4, 154.2, 166.6, 167.8, 187.9.

HRMS (ESI-TOF) m/z calcd. for $C_{27}H_{29}N_2O_4$ $[M+H]^+$: 445.2122; found 445.2151.

$[\alpha]_D^{25}$= -22.3 (c = 0.65, $CHCl_3$).

7. Intermediate Characterization: ESI/MS Studies of the Asymmetric Organocatalytic Conjugate Addition of Cyclic Ketones to Dehydroalanine Catalyzed by Chiral Primary Amine Thiourea Bifunctional Catalysts

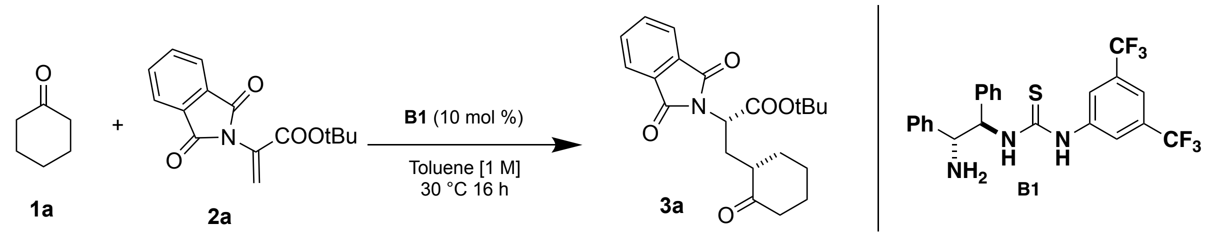

The ESI-MS analysis refers to the reaction under the optimized conditions (20 mol % of **B1**, 1.5 equiv **2a** and [**1a**] = 1 M in toluene). In a first experiment, detailed in Figure S1, the ESI-MS spectrum in positive mode of the catalyst **B1** showed the m/z 484.0, which can fragment to yield ions at m/z 450.0 (loss of 34 u) upon loss of H_2S. To rationalize the loss of H_2S and the formation of the carbodiimide ion at m/z 450 starting from the thiourea-compound **B1**, we assume as reported in literature[5] preceding tautomerization upon collisional activation that converts the predominant thione to the respective enthiol tautomer, which then eventually cleaves off H_2S in a subsequent fragmentation reaction. A second ESI-MS spectrum in positive mode, detailed in Figure S2, obtained from catalyst **B1** and **2a** showed the catalyst at m/z 484.0, which can fragment to yield ions at m/z 450.0 (loss of 34 u) upon loss of H_2S and the complex obtained from catalyst and **2a** at m/z 757, where both the reacting partners are brought together in close proximity, which can fragment to yield ions at m/z 723.2 (loss of 34 u) upon loss of H_2S.

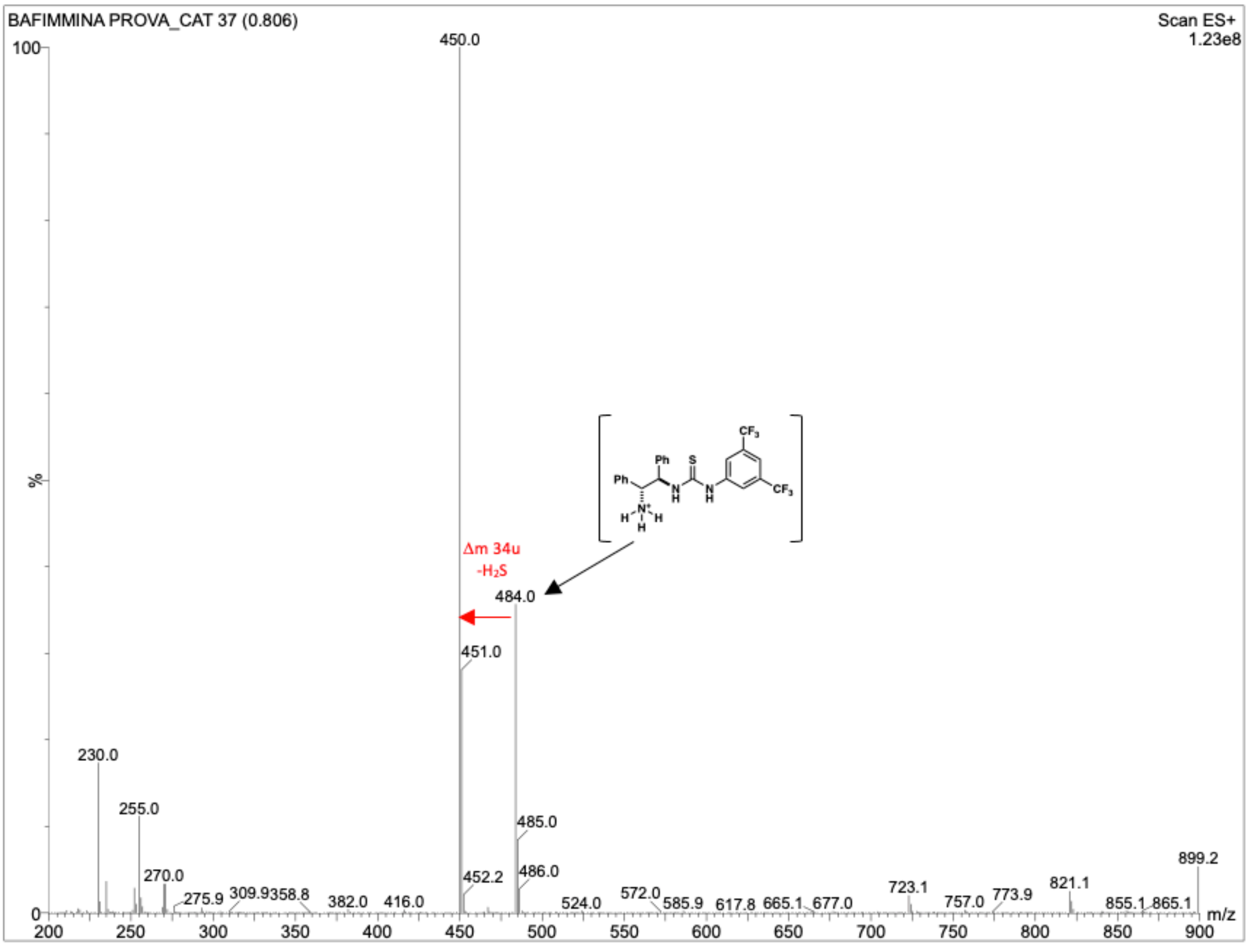

Figure S1. Detectable intermediates by ESI-MS analysis in positive mode of the catalyst **B1**.

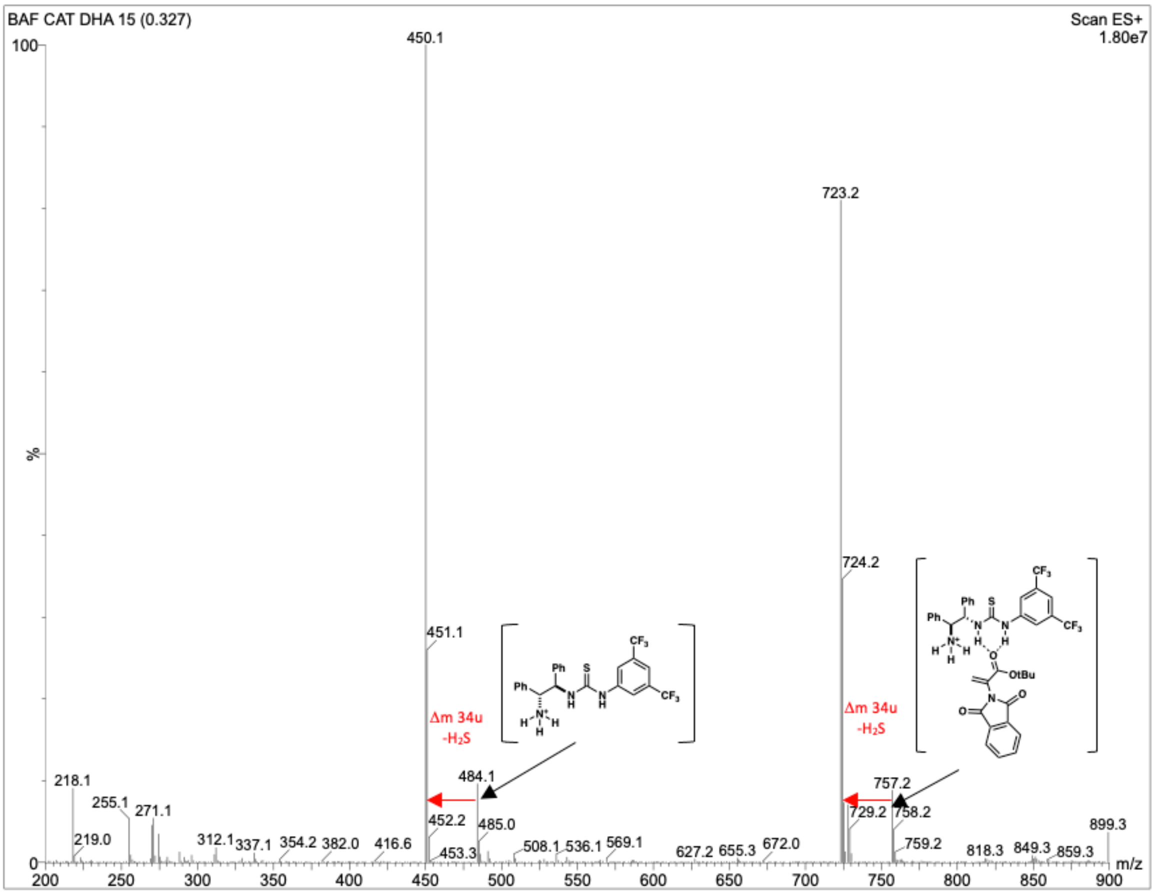

Figure S2. Detectable intermediates by ESI-MS analysis in positive mode of the complex obtained from catalyst **B1** and DHA.

In a third spectrum, detailed in Figure S3, the ESI-MS spectrum in positive mode of the reaction mixture showed the presence of the catalyst at m/z 484.1 (which can fragment to yield ions at m/z 450.0 (loss of 34 u) upon loss of H_2S), of the imine precursor at m/z 564.2, of the complex obtained from catalyst and **2a** at m/z 757 (which can fragment to yield ions at m/z 723.2 (loss of 34 u) upon loss of H_2S) and the complex obtained from imine and **2a** at m/z 837.3, also in this case, where both the reacting partners are brought together in close proximity. The analyzed sample was collected 5 minutes after the start of the reaction. A concomitant ^{1}H NMR analysis did not show any detectable trace of the product, which would provide the same isotopic pattern of complex.

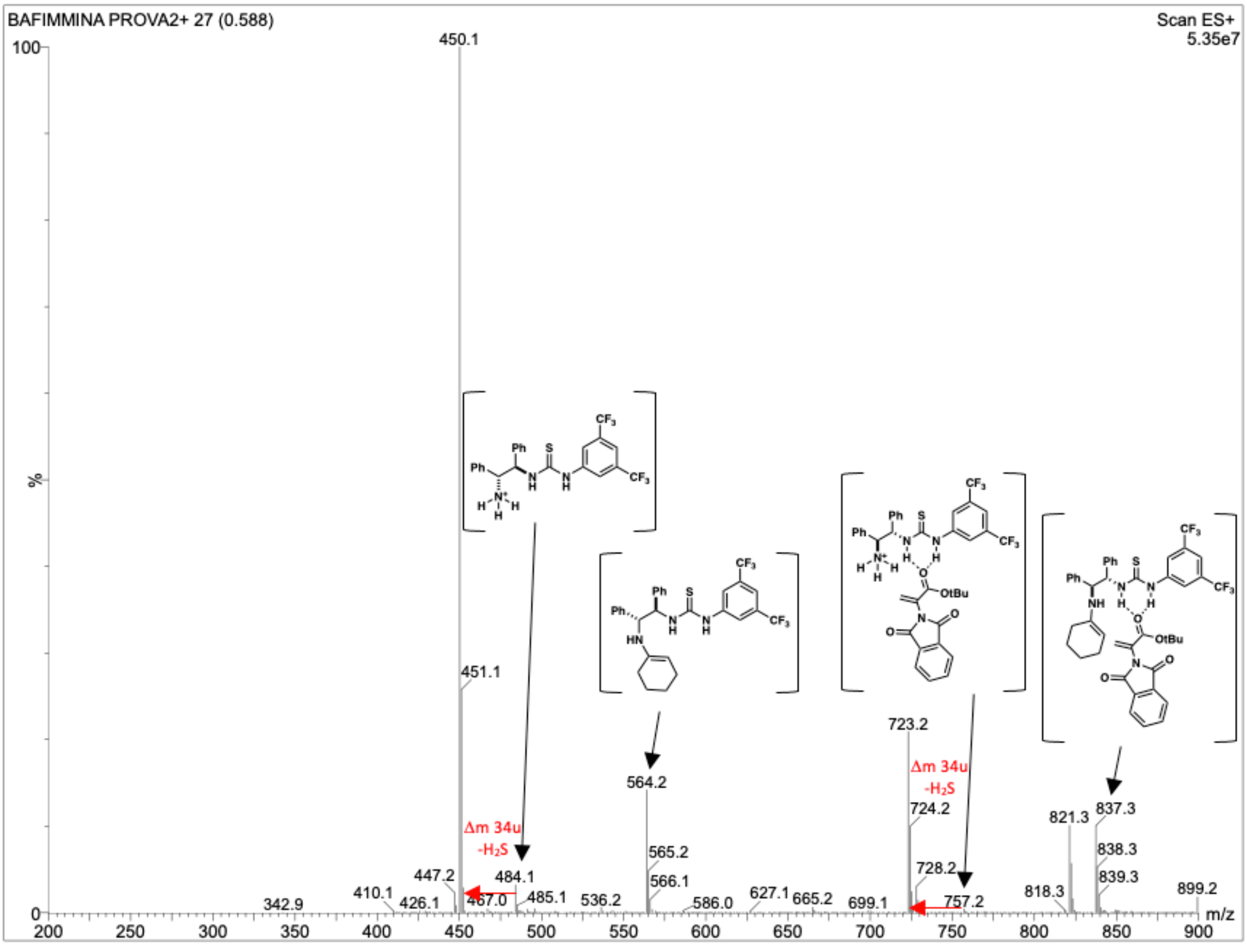

Figure S3. Detectable intermediates by ESI-MS analysis in positive mode of the reaction mixture. The analyzed sample was collected 5 minutes after the start of the reaction.

8. References:

[1] B. M. Trost, G. R. Dake, *J. Am. Chem. Soc.* **1997**, *119*, 7595 – 7596.

[2] F. Yu, Z. Jin, H. Huang, T. Ye, X. Liang, J. Ye, *Org. Biomol. Chem.* **2010**, *8*, 4767-4774.

[3] E. Arceo, A. Bahamonde, G. Bergonzini, P. Melchiorre, *Chem. Sci.* **2014**, *5*, 2438–2442.

[4] C. J. Blankley, J. S. Kaltenbronn, D. E. DeJohn, A. Werner, L. R. Bennett, G. Bobowski, U. Krolls, D. R. Johnson, W. M. Pearlman, M. L. Hoefle, A. D. Essenburg, D. M. Cohen, H. R. Kaplan, *J. Med. Chem.* **1987**, *30*, 992-998.

[5] F. Falvo, L. Fiebig, F. Dreiocker, R. Wang, P.B. Armentrout, M. Schäfer, *Int. J. Mass Spectrom*. **2012**, 330–332.

9. NMR spectra

^{1}H NMR, COSY and ^{13}C NMR of (*S*)-*tert*-butyl 2-(1,3-dioxoisoindolin-2-yl)-3-((*S*)-2-oxocyclohexyl)propanoate (3a)

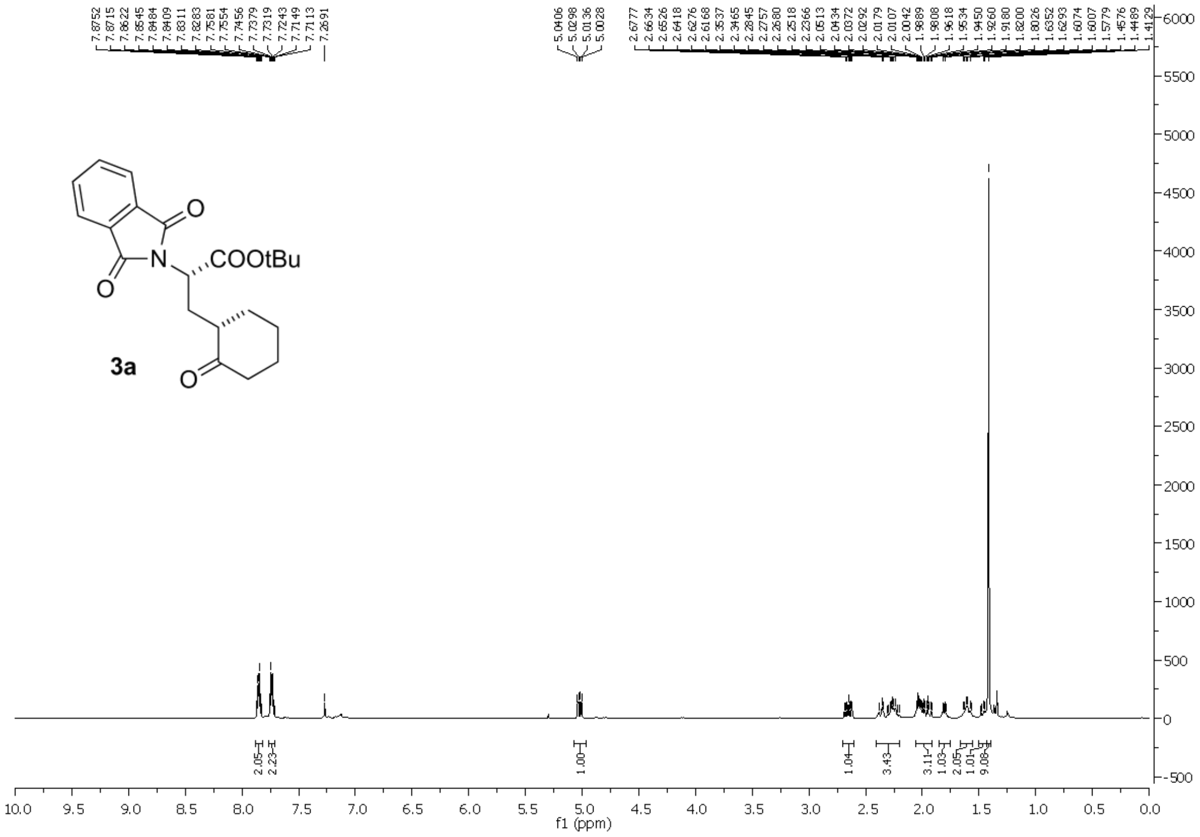

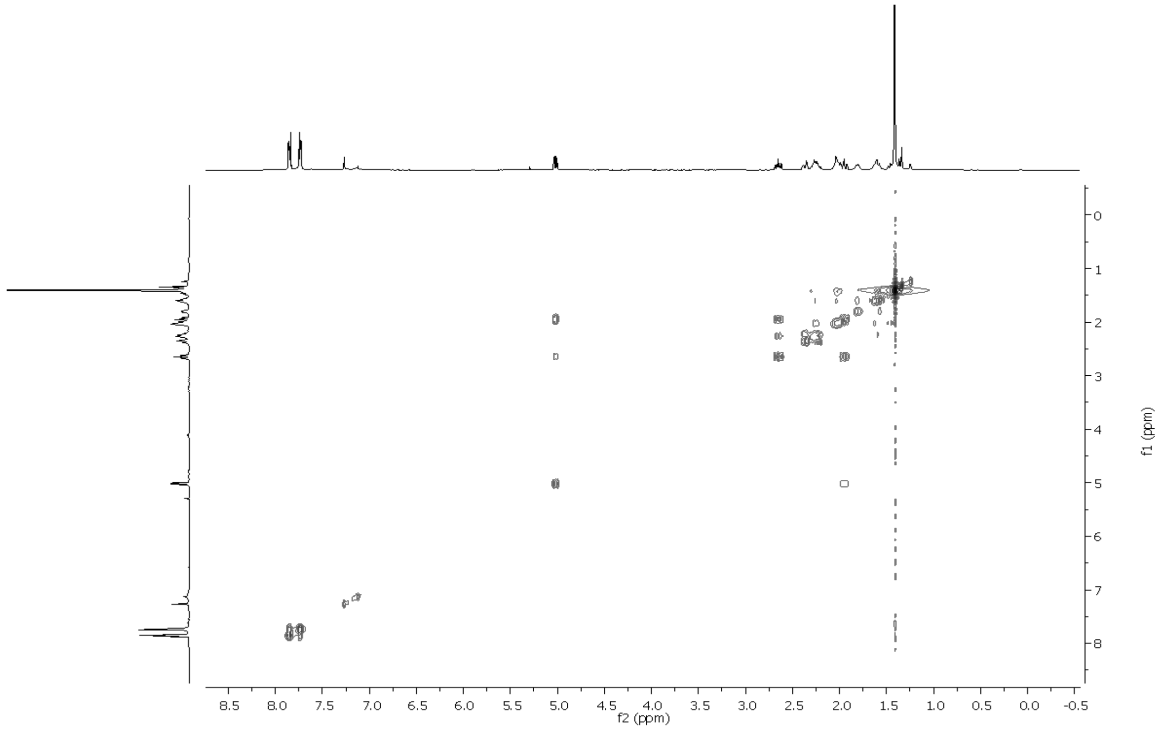

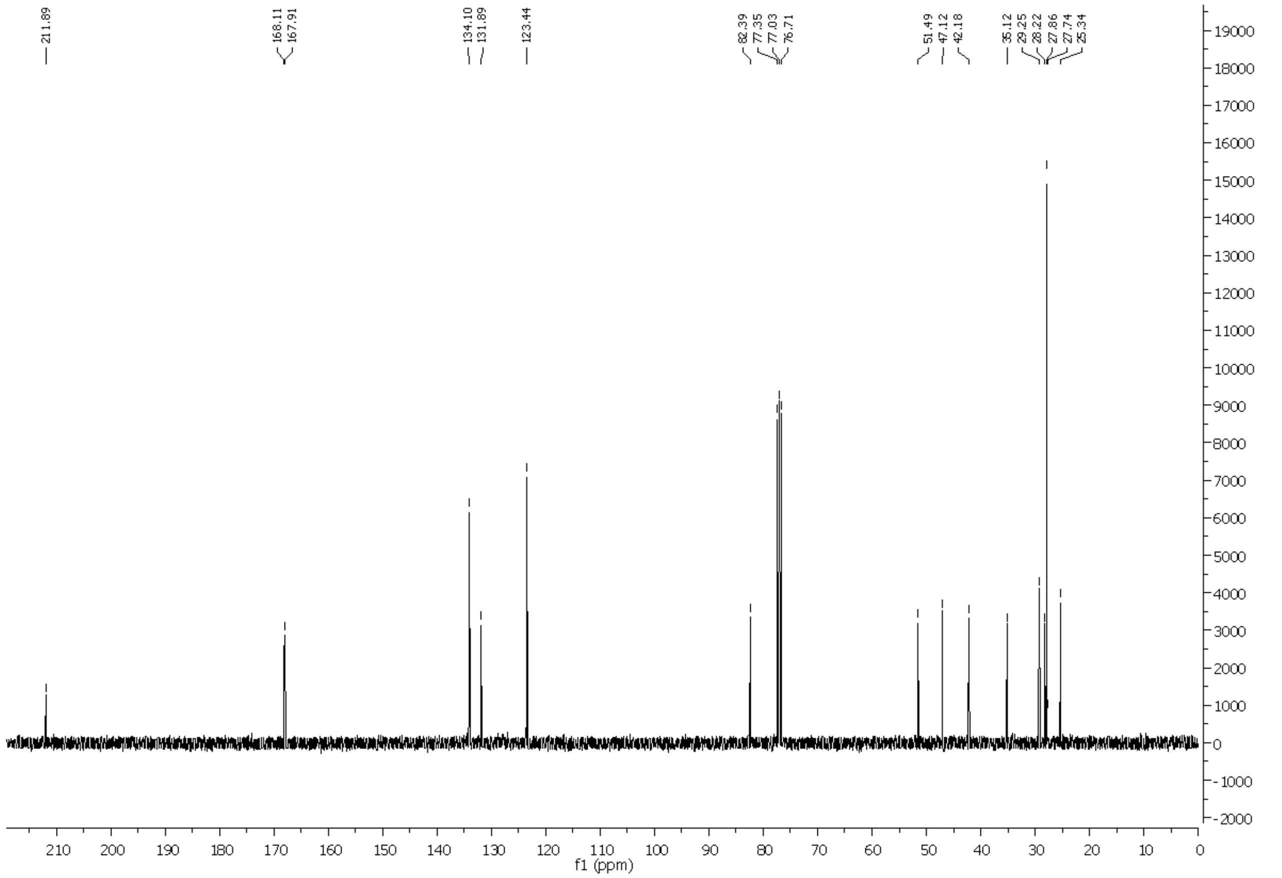
211.89
168.11
167.91
134.10
131.89
123.44
82.39
77.35
77.03
76.71
51.49
47.12
42.18
35.12
29.25
28.22
27.86
27.74
25.34
f1 (ppm)

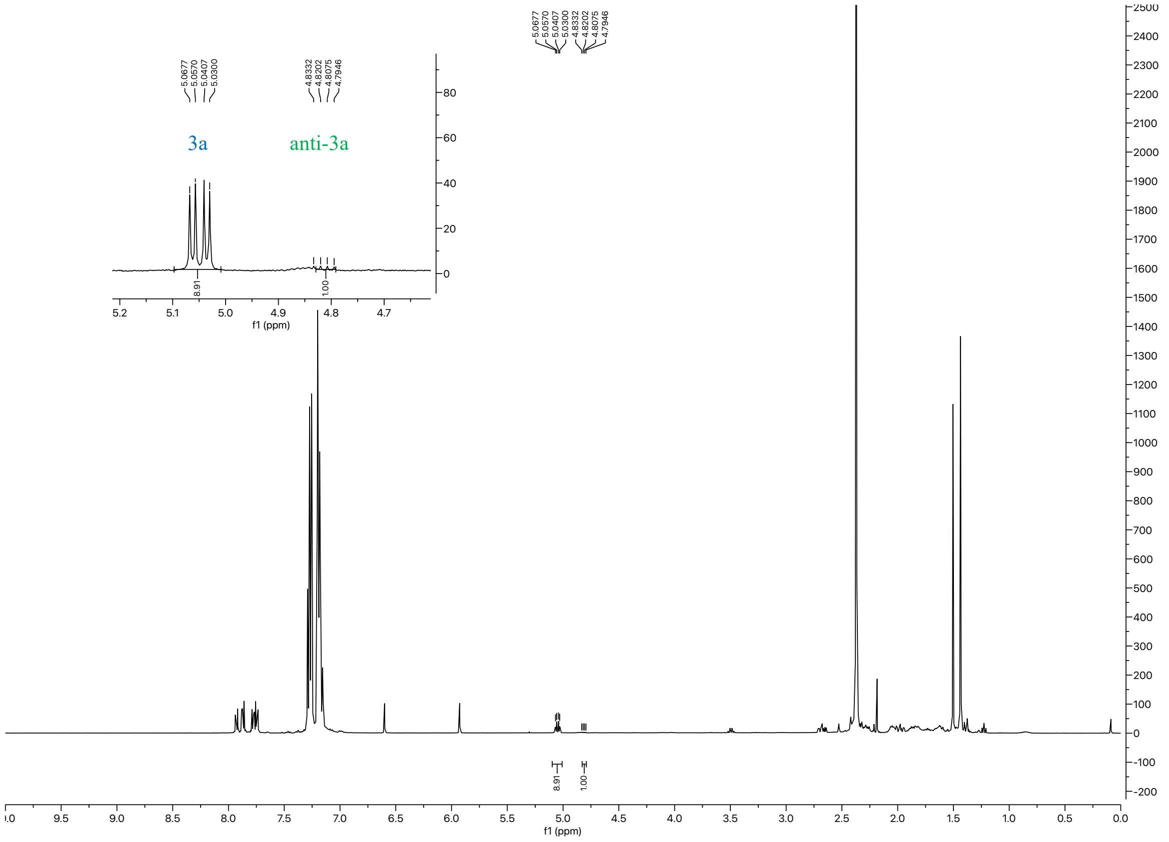
5.0677
5.0570
5.0407
5.0300
4.8332
4.8202
4.8075
4.7946
3a
anti-3a
8.91
1.00
f1 (ppm)

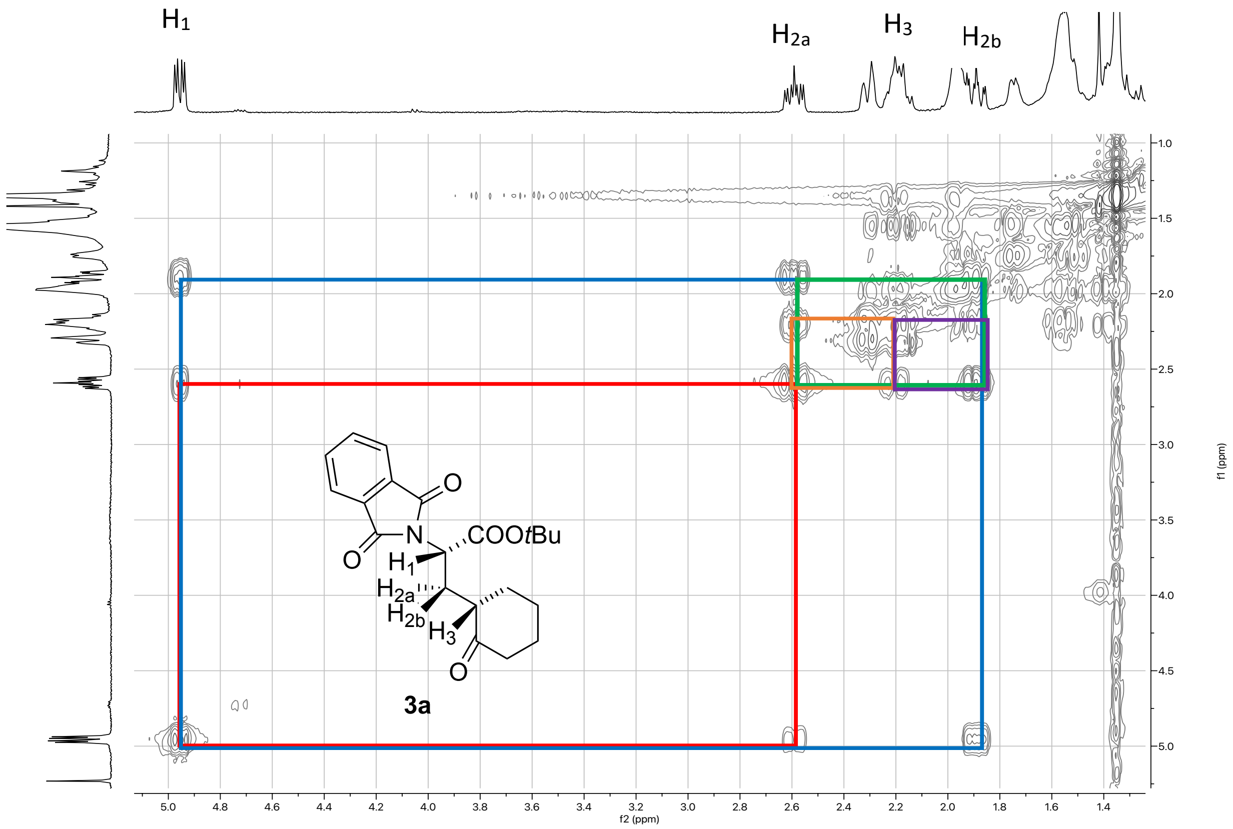
H1
H2a
H3
H2b
COOtBu
N
O
O
O
H1
H2a
H2b
H3
3a
f2 (ppm)
f1 (ppm)

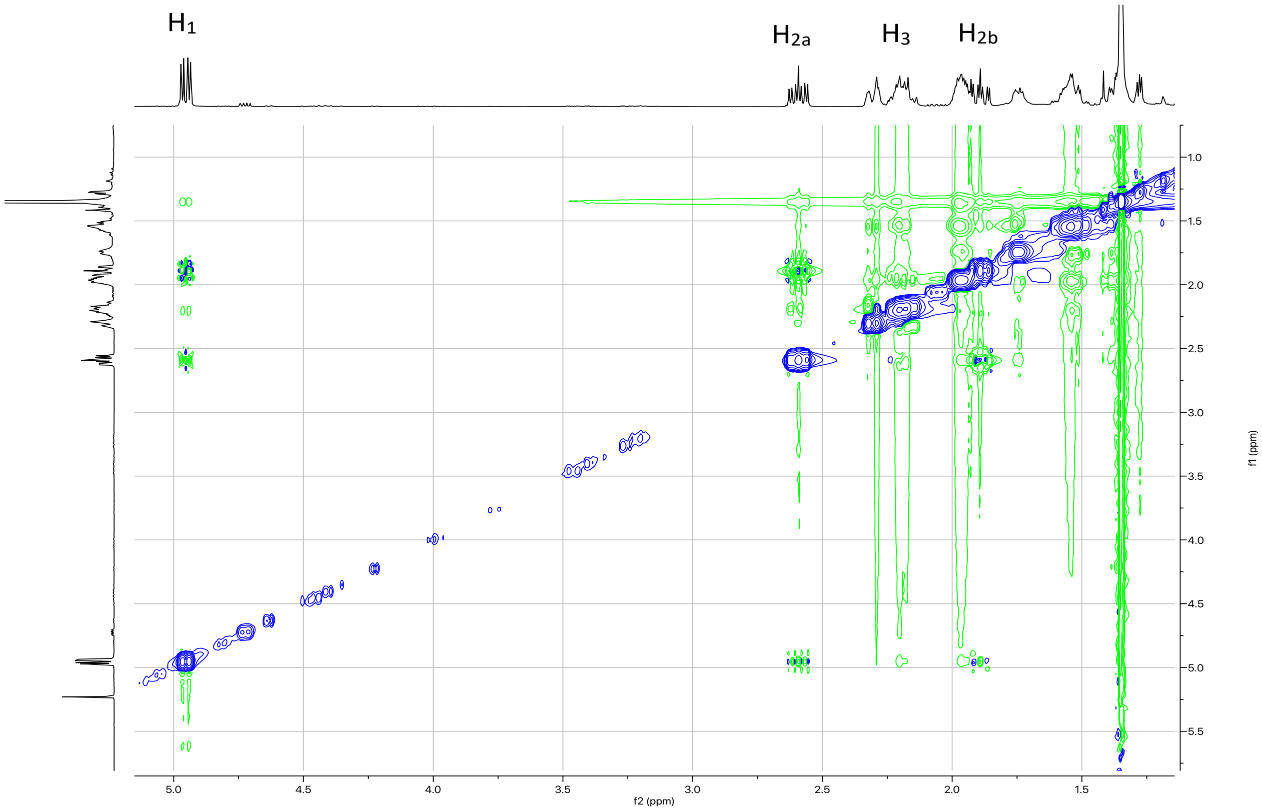
H1
H2a
H3
H2b
f2 (ppm)
f1 (ppm)

Observed nOe correlations:

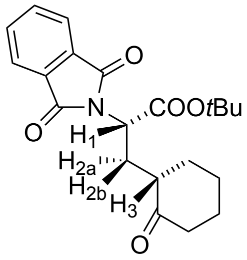

3a

- H_1 (4.95 ppm) and H_{2b} (1.89 ppm)
- H_1 (4.95 ppm) and H_{2a} (2.60 ppm), low intensity correlation
- H_{2a} (2.60 ppm) and H_3 (2.20 ppm) low intensity correlation
- H_1 (4.95) and H_3 (2.20 ppm) low intensity correlation

^{1}H NMR, COSY and ^{13}C NMR (*S*)-*tert*-butyl 2-(1,3-dioxoisoindolin-2-yl)-3-((*S*)-2-oxocyclopentyl)propanoate (3b)

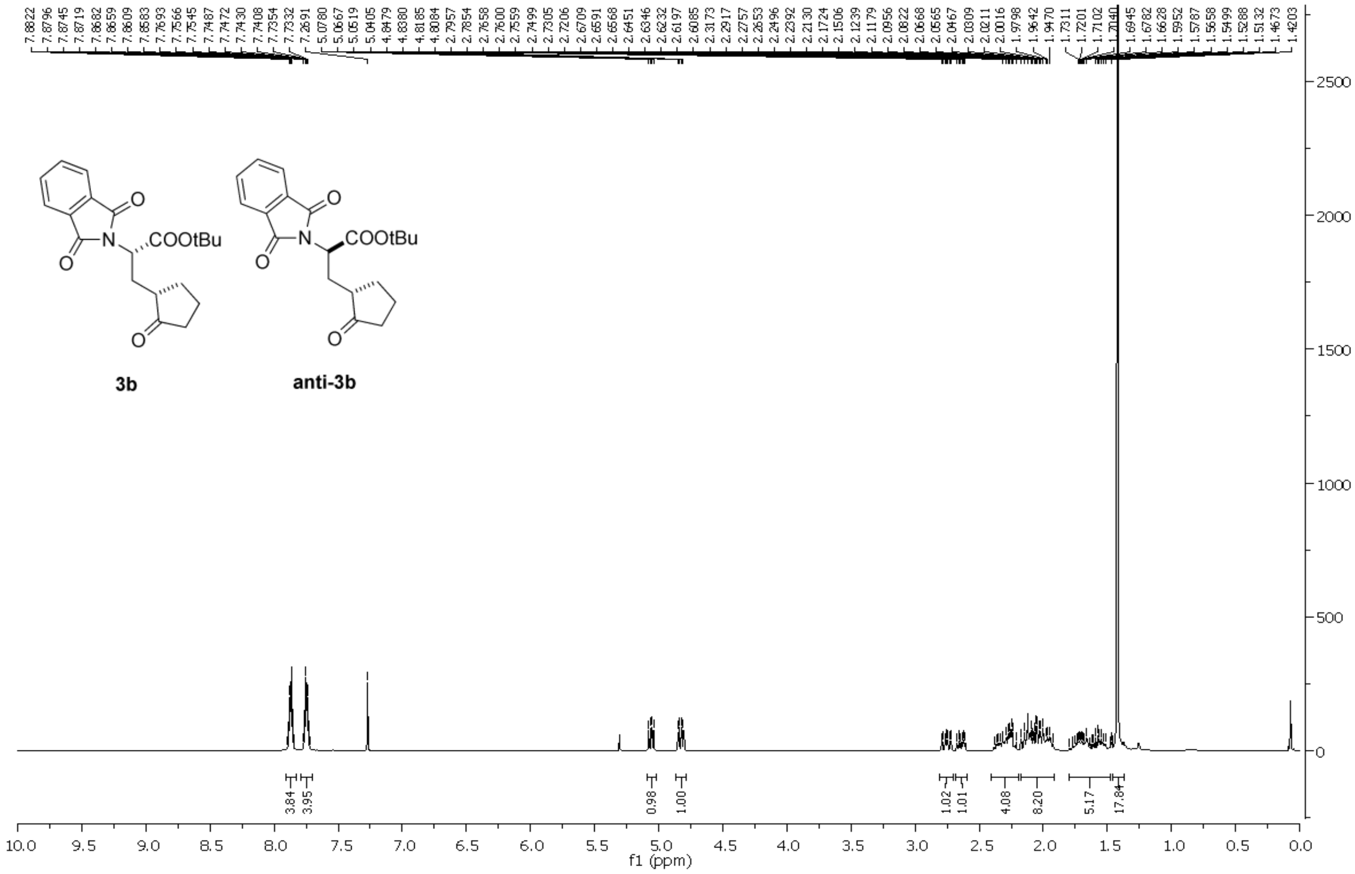

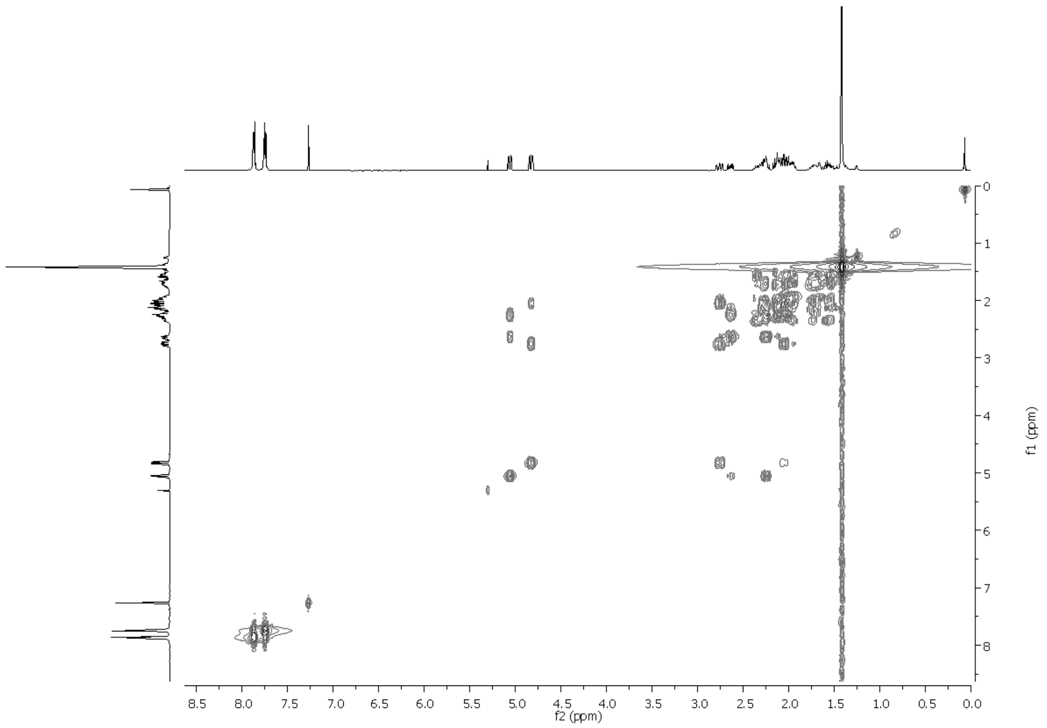

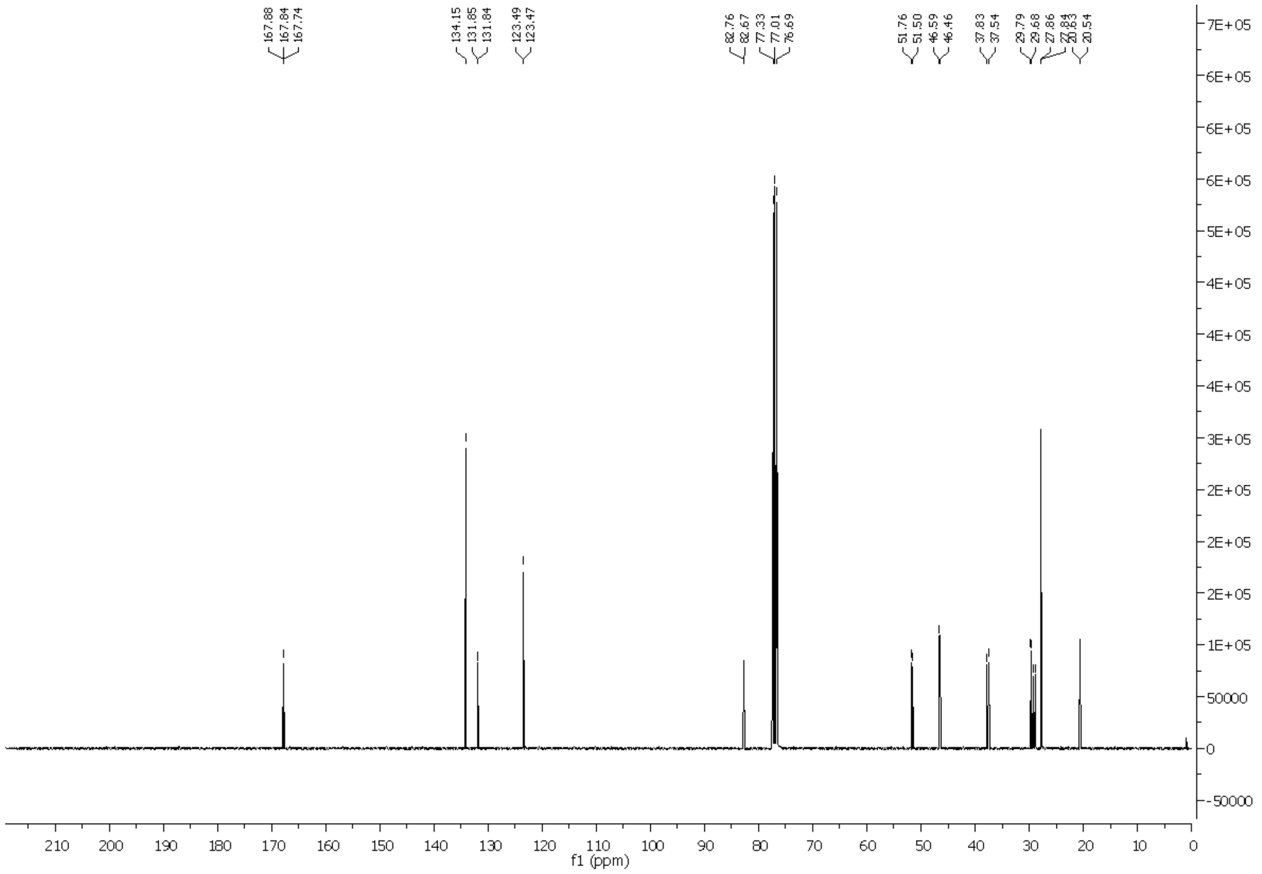
167.88
167.84
167.74
134.15
131.85
131.84
123.49
123.47
82.76
82.67
77.33
77.01
76.69
51.76
51.50
46.59
46.46
37.83
37.54
29.79
29.68
27.86
27.84
20.63
20.54
f1 (ppm)

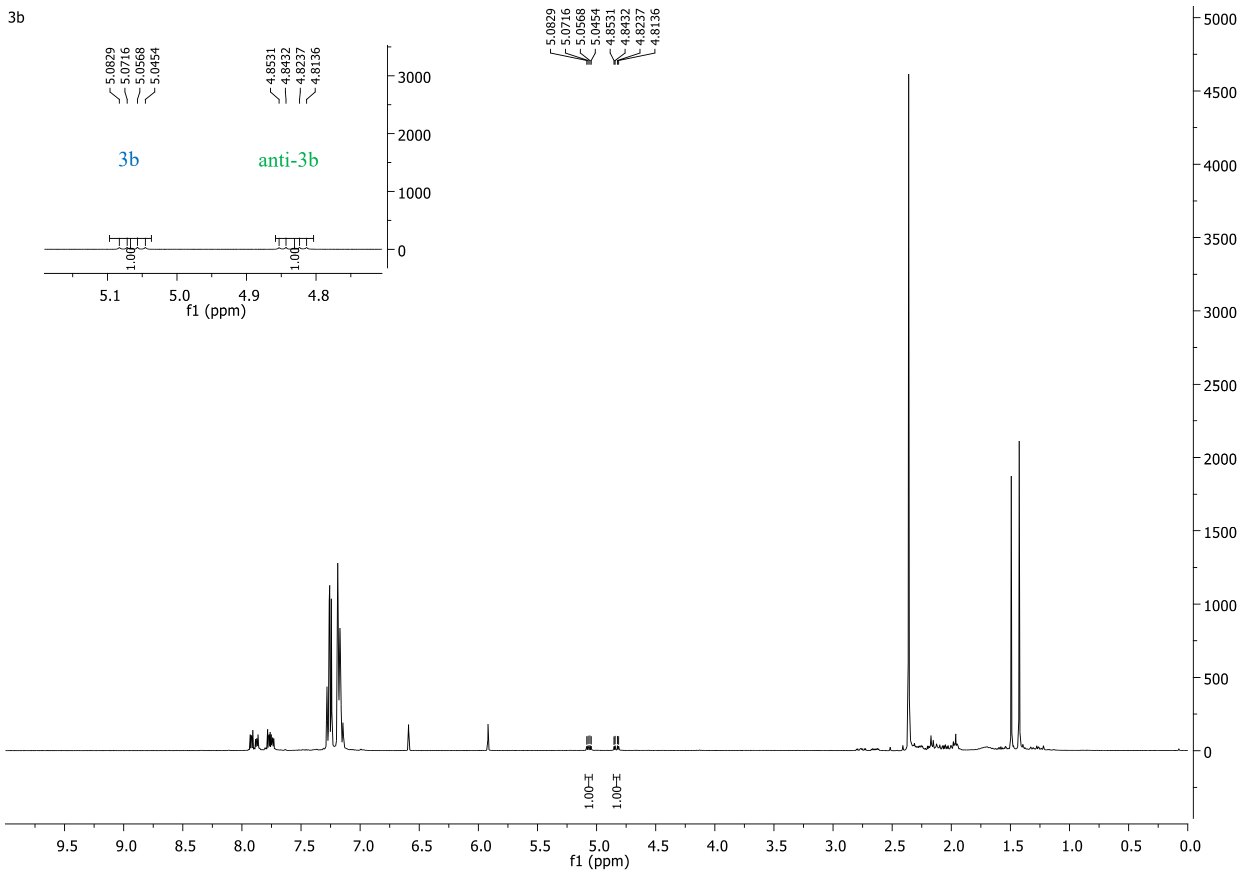
3b
5.0829
5.0716
5.0568
5.0454
4.8531
4.8432
4.8237
4.8136
3b
anti-3b
1.00
1.00
f1 (ppm)

^{1}H NMR, COSY and ^{13}C NMR (*S*)-*tert*-butyl 2-(1,3-dioxoisoindolin-2-yl)-3-((*S*)-4-oxotetrahydro-2*H*-pyran-3-yl)propanoate (3d)

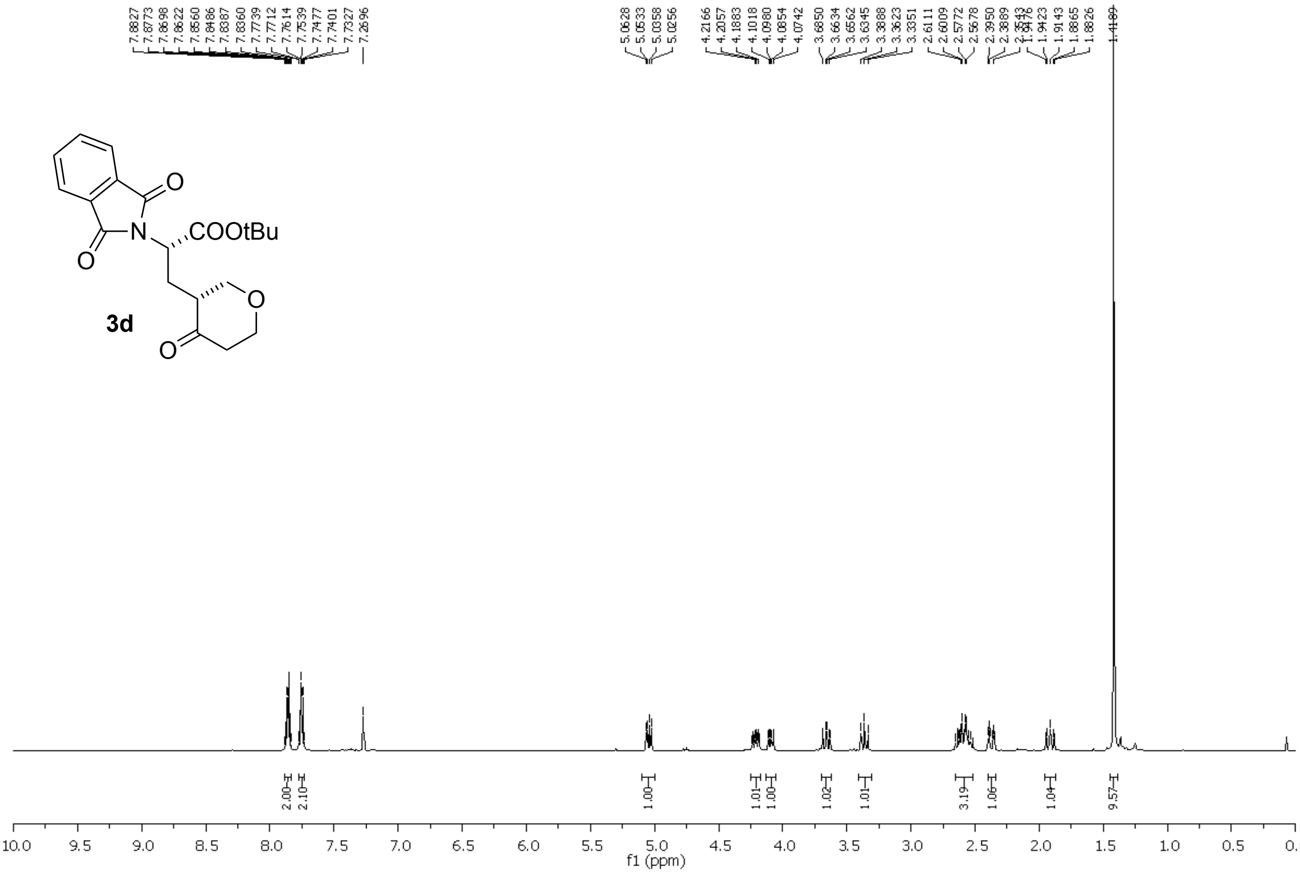

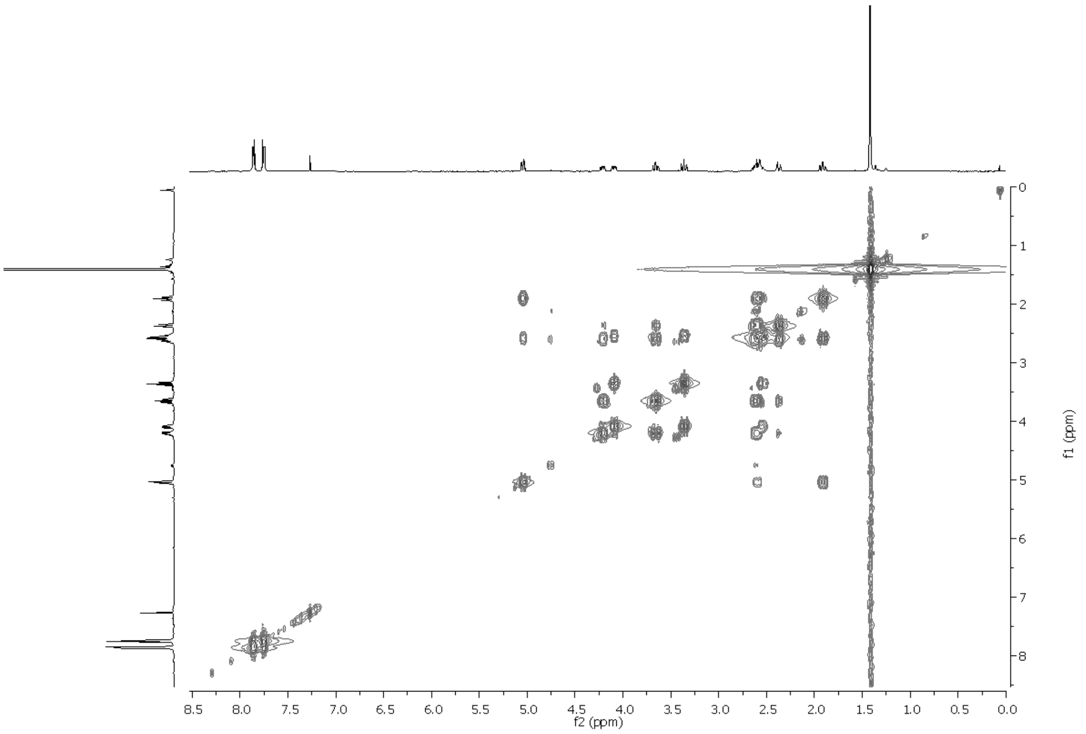

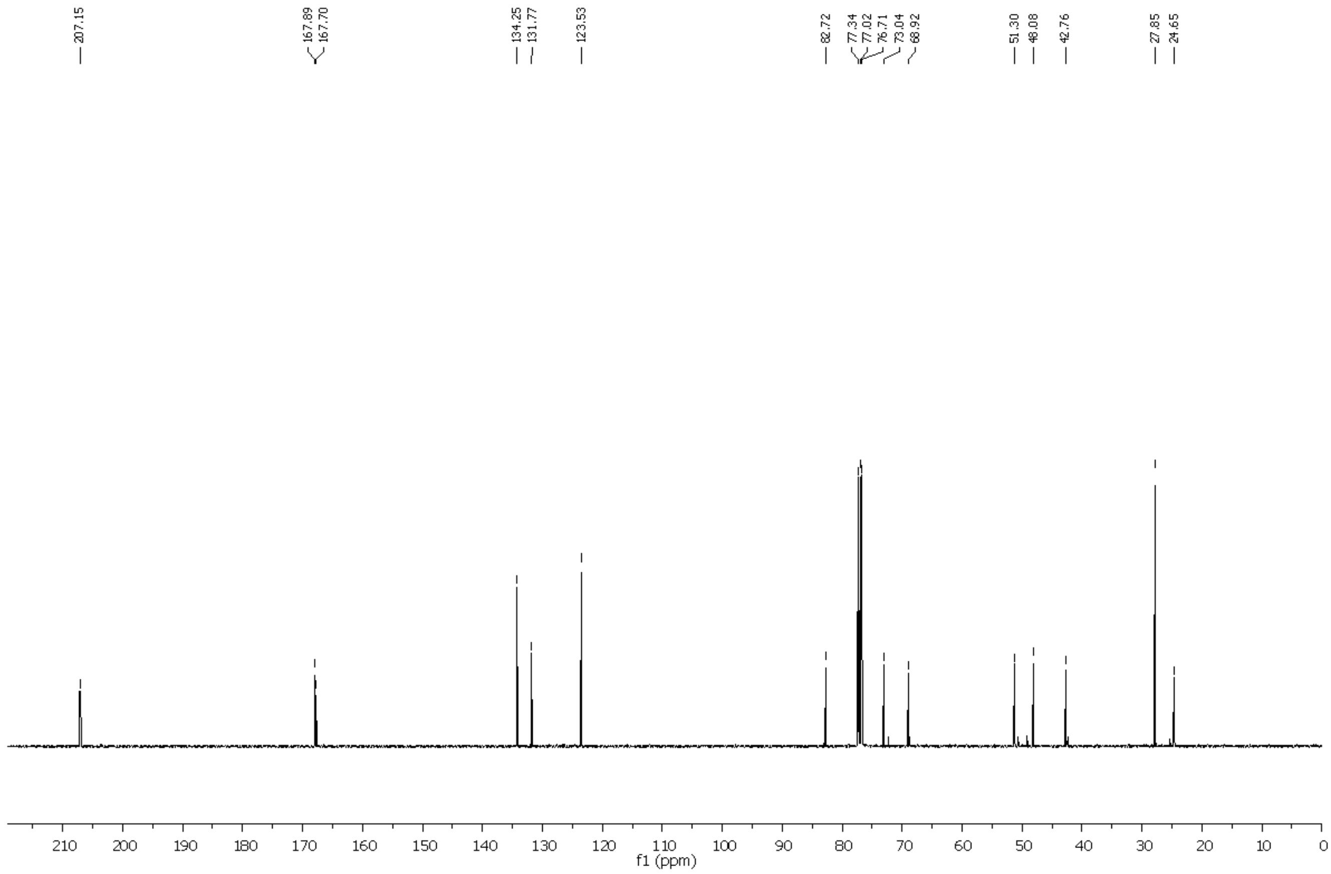
207.15
167.89
167.70
134.25
131.77
123.53
82.72
77.34
77.02
76.71
73.04
68.92
51.30
48.08
42.76
27.85
24.65
f1 (ppm)

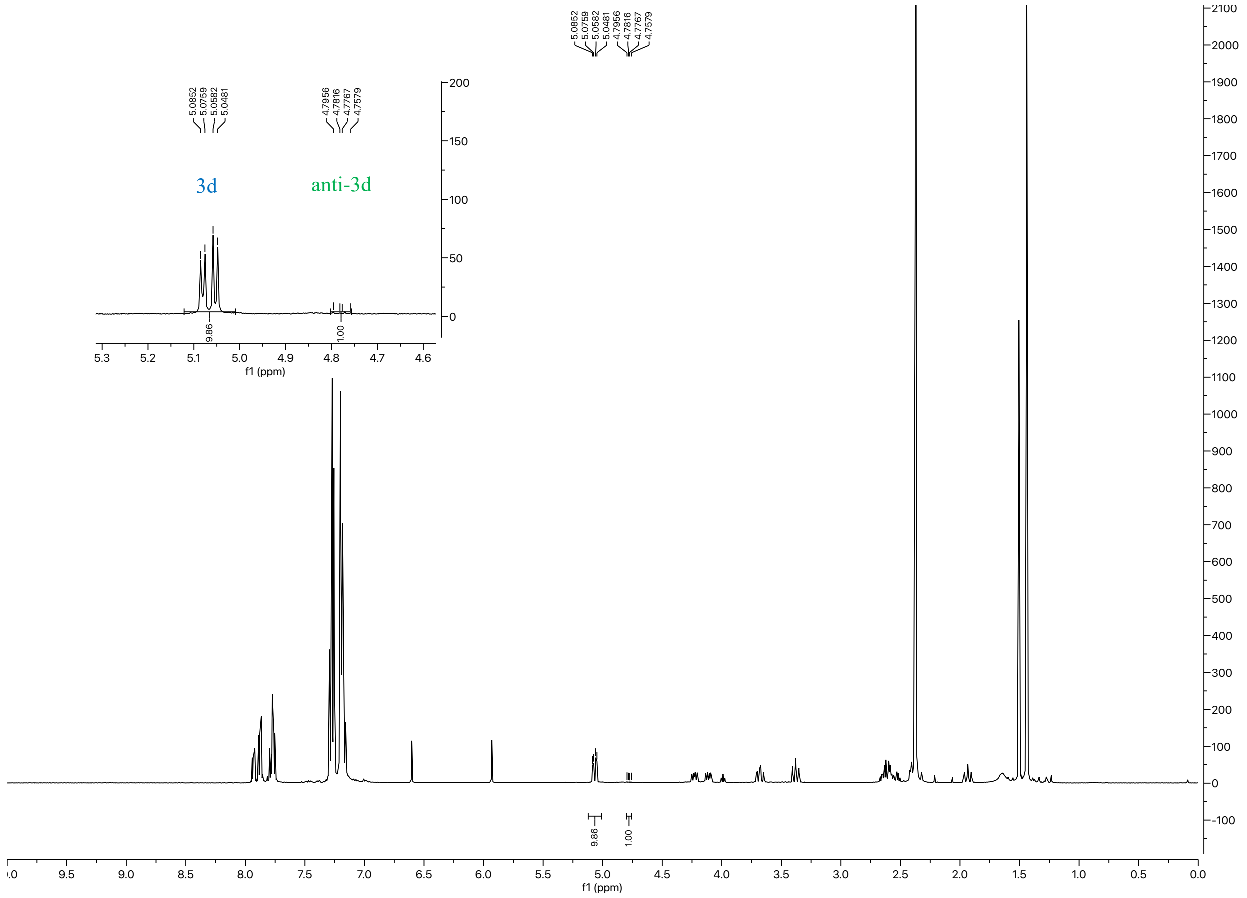
5.0852
5.0759
5.0582
5.0481
4.7956
4.7816
4.7767
4.7579
3d
anti-3d
9.86
1.00
f1 (ppm)

^{1}H NMR, COSY and ^{13}C NMR of (*S*)-*tert*-butyl 3-((*S*)-3-*tert*-butoxy-2-(1,3-dioxoisoindolin-2-yl)-3-oxopropyl)-4-oxopiperidine-1-carboxylate (3e)

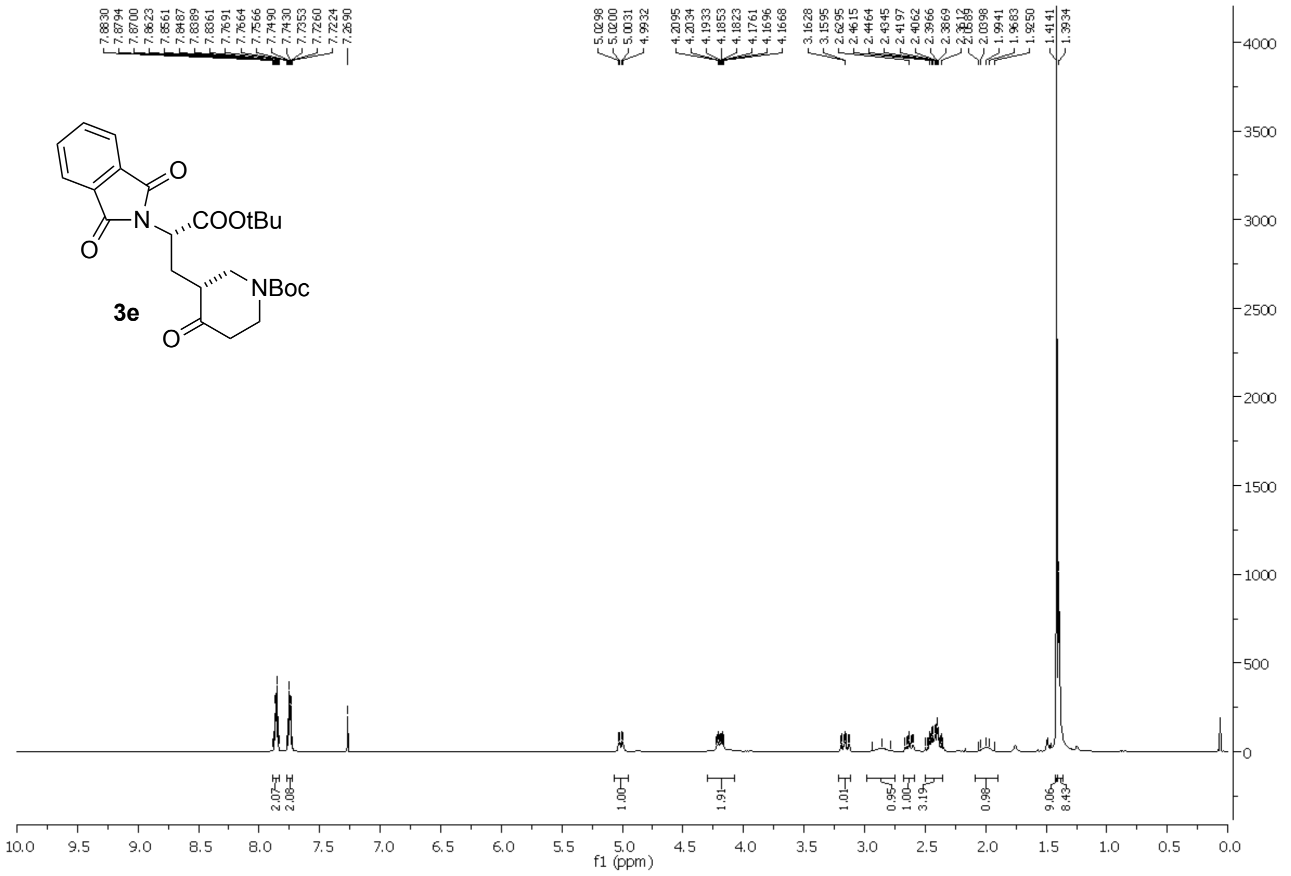

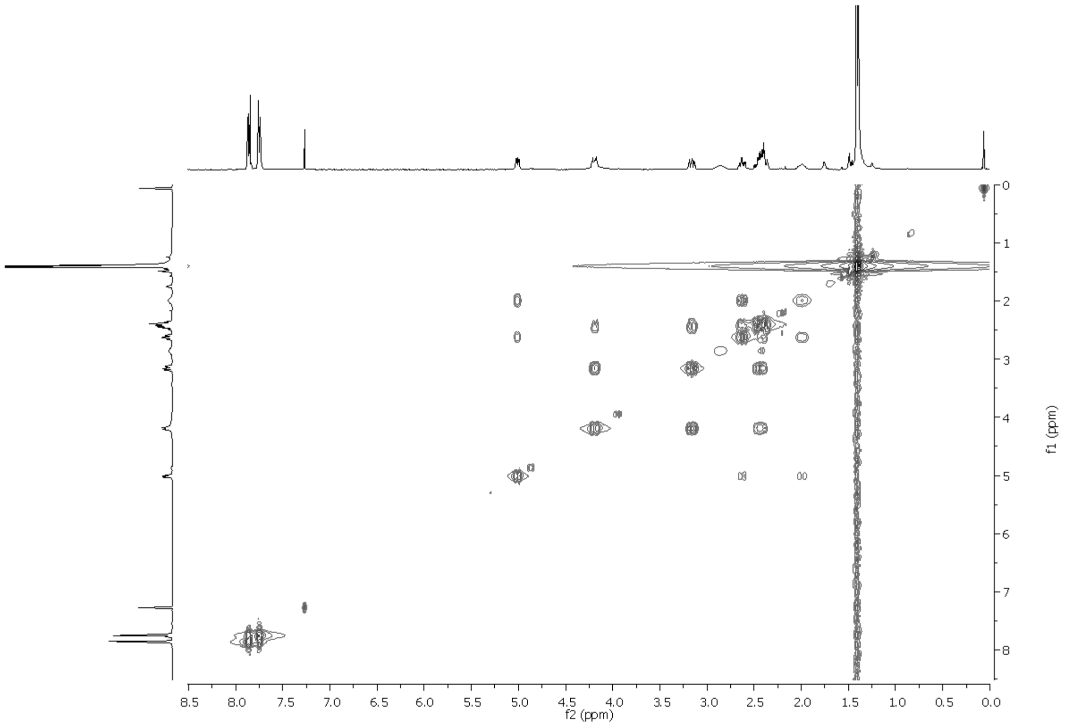

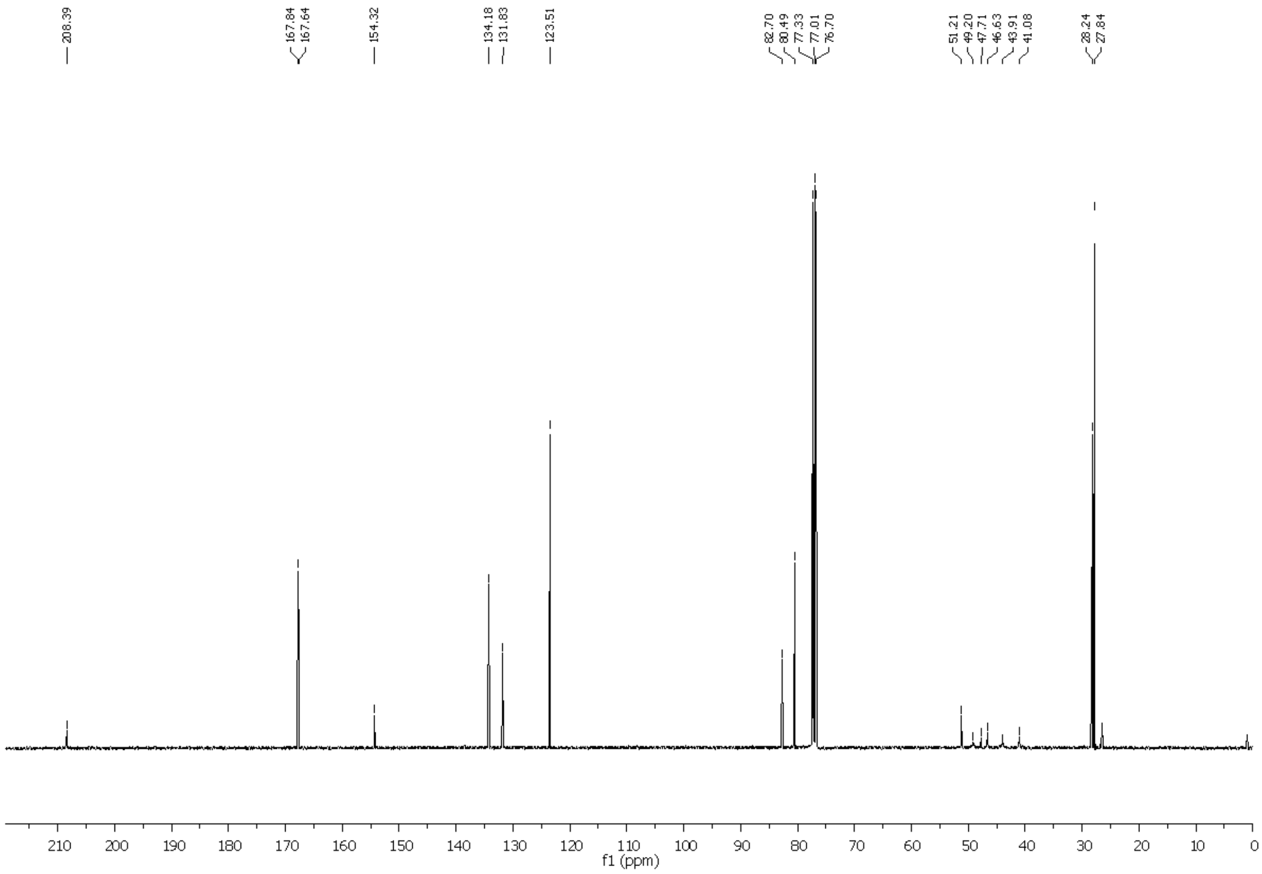
208.39
167.84
167.64
154.32
134.18
131.83
123.51
82.70
80.49
77.33
77.01
76.70
51.21
49.20
47.71
46.63
43.91
41.08
28.24
27.84
f1 (ppm)

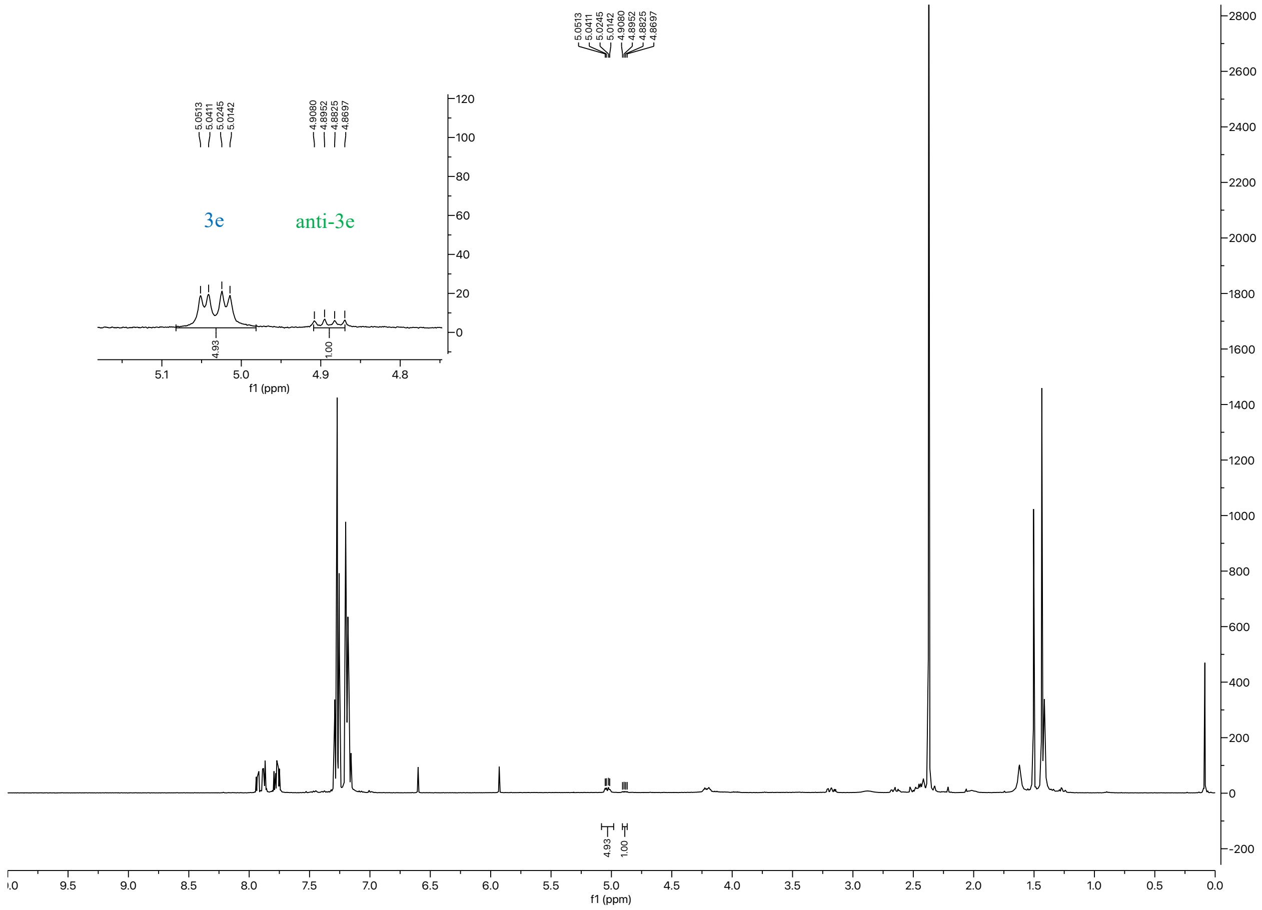
5.0513
5.0411
5.0245
5.0142
4.9080
4.8952
4.8825
4.8697
3e
anti-3e
4.93
1.00
f1 (ppm)

^{1}H NMR, COSY and ^{13}C NMR of (*S*)-*tert*-butyl 2-(1,3-dioxoisoindolin-2-yl)-3-((*S*)-4-oxotetrahydro-2*H*-thiopyran-3-yl)propanoate (3f)

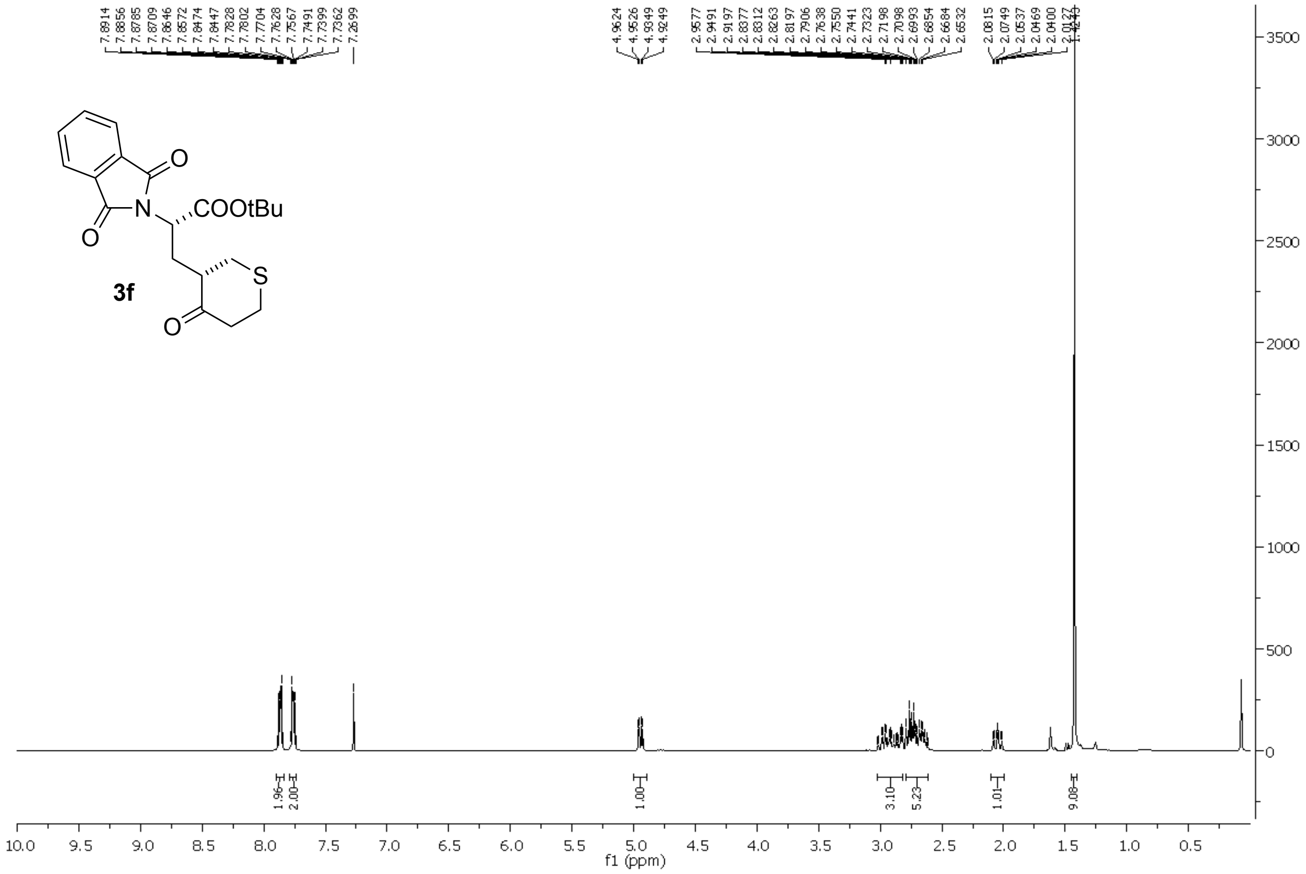

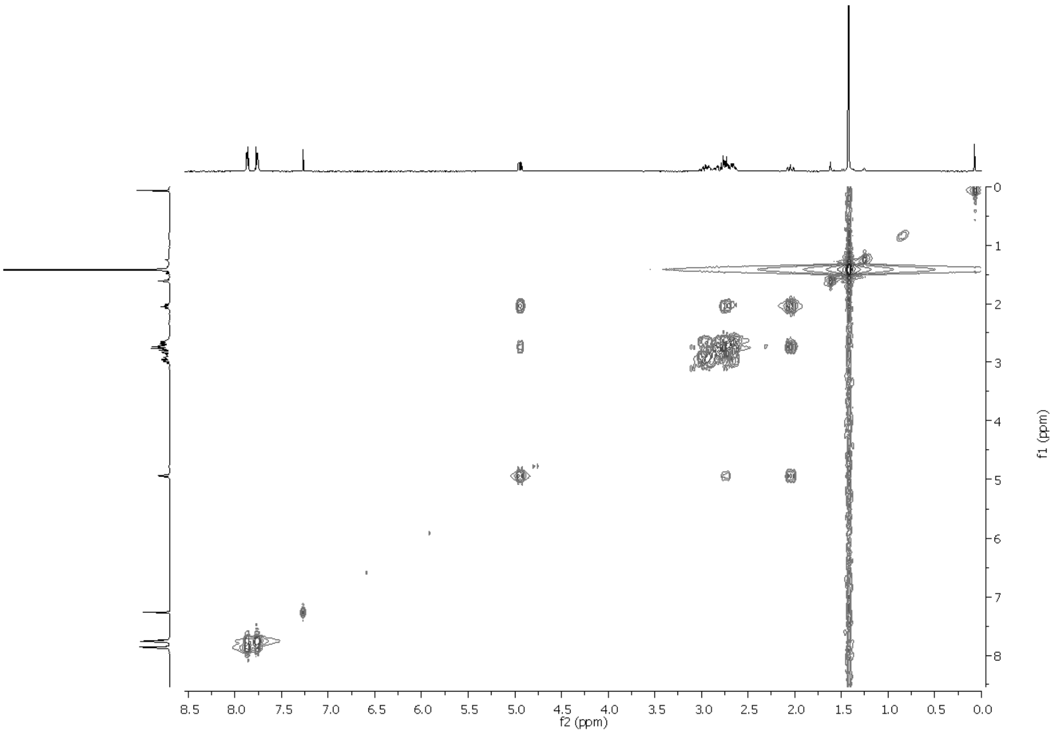

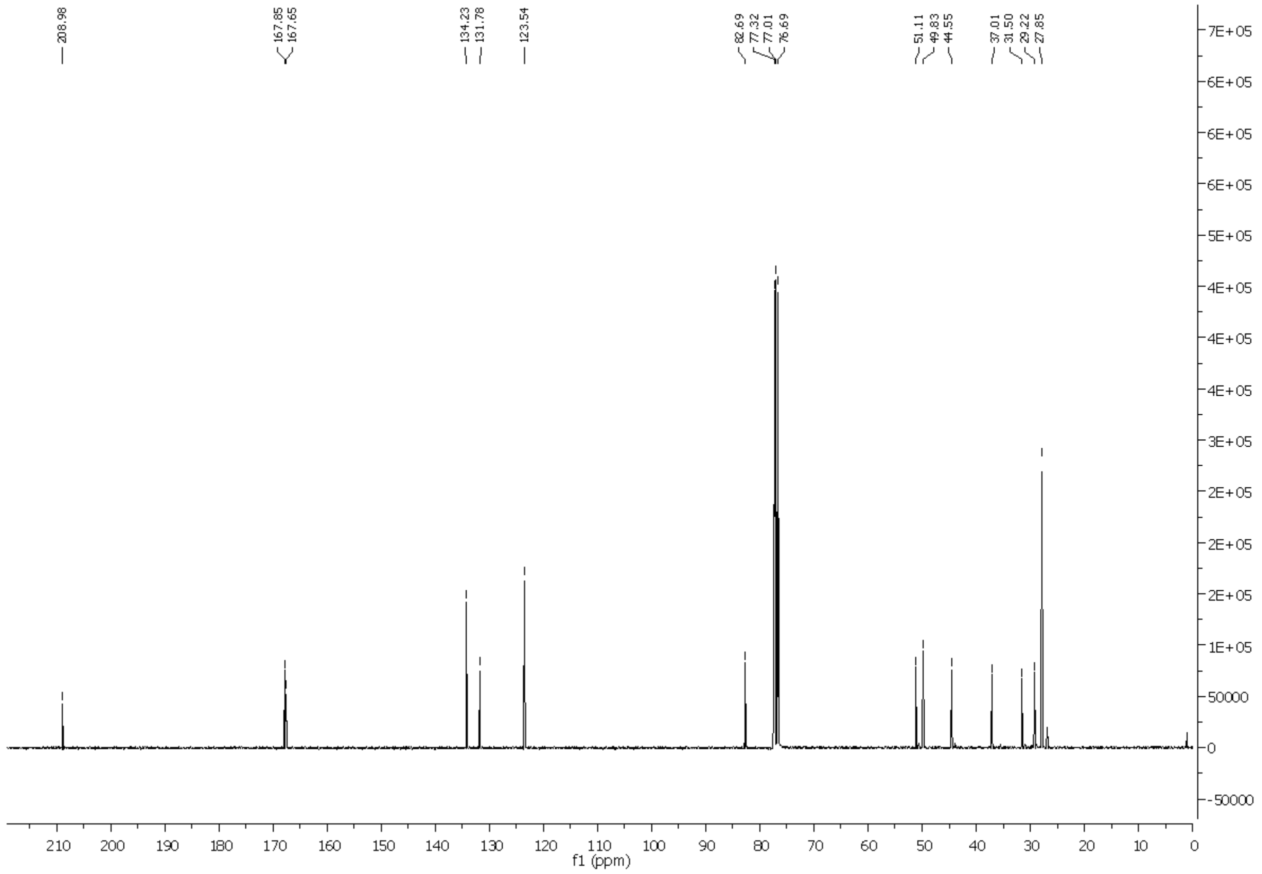

208.98
167.85
167.65
134.23
131.78
123.54
82.69
77.32
77.01
76.69
51.11
49.83
44.55
37.01
31.50
29.22
27.85
f1 (ppm)

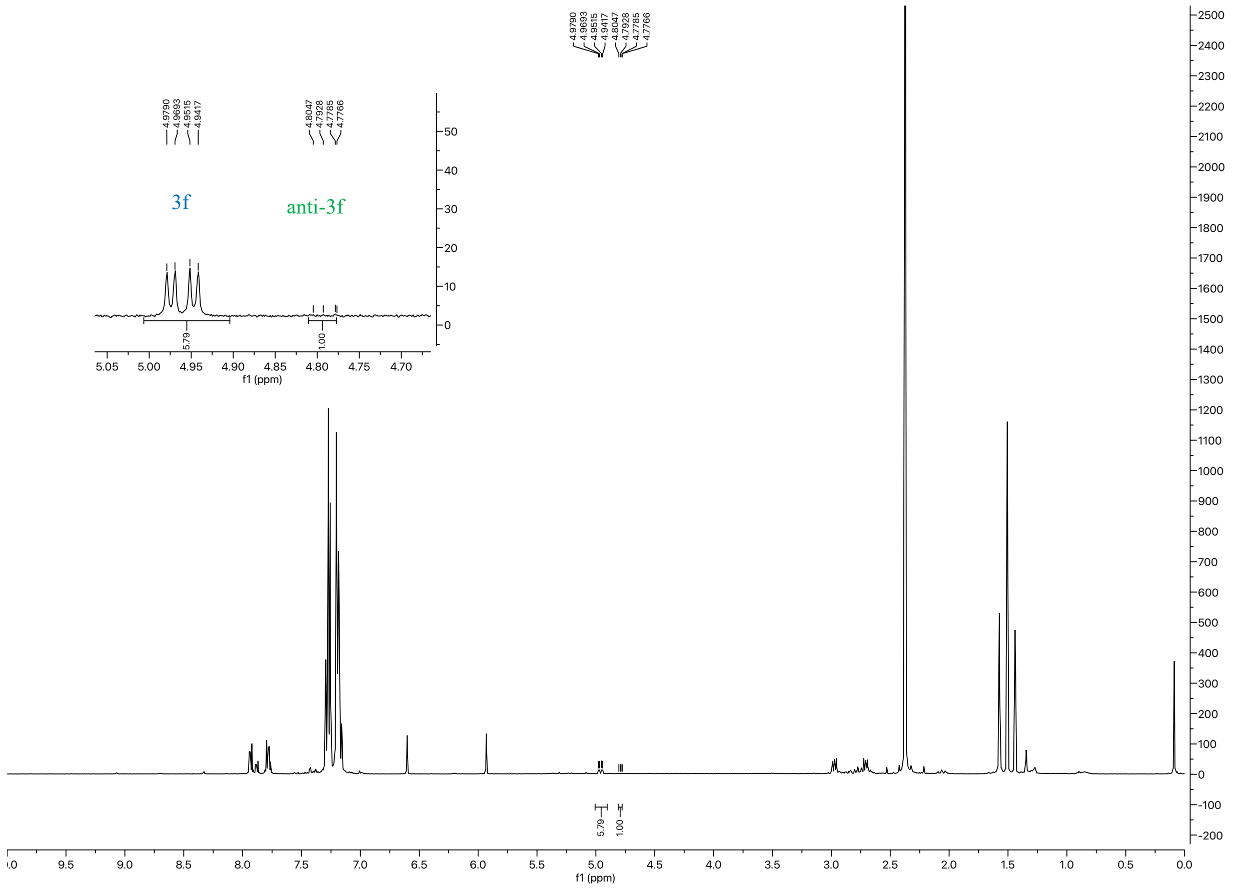

4.9790
4.9693
4.9515
4.9417
4.8047
4.7928
4.7785
4.7766
3f
anti-3f
5.79
1.00
f1 (ppm)

^{1}H NMR, COSY and ^{13}C NMR (*S*)-*tert*-butyl 2-(1,3-dioxoisoindolin-2-yl)-3-((*S*)-8-oxo-1,4-dioxaspiro[4.5]decan-7-yl)propanoate (3g)

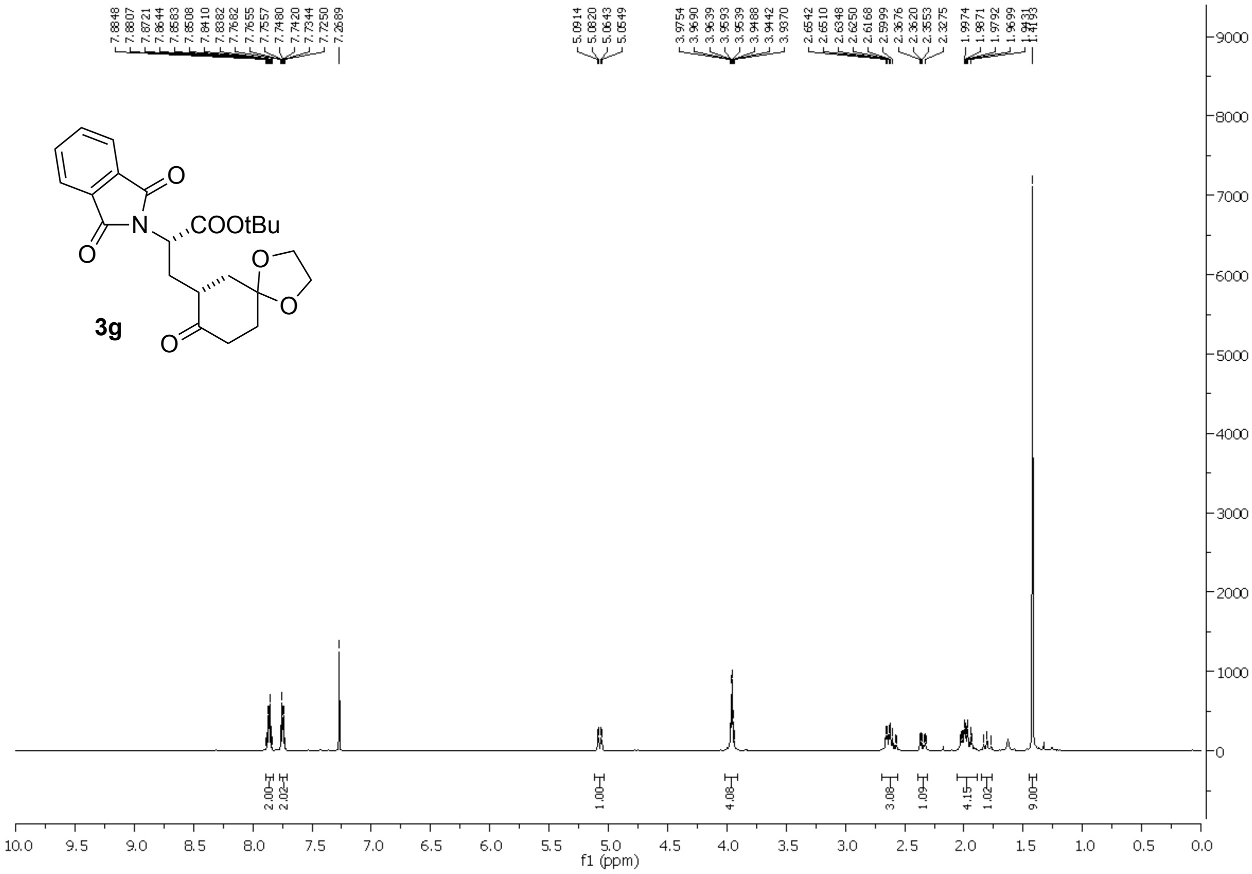

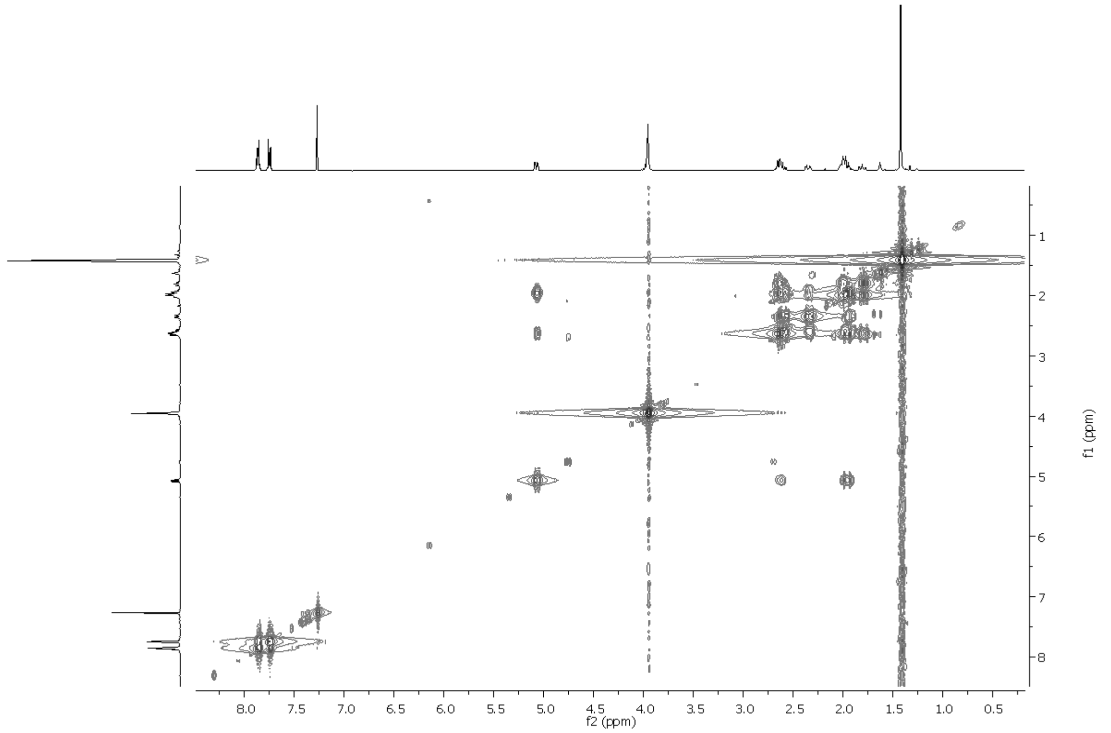

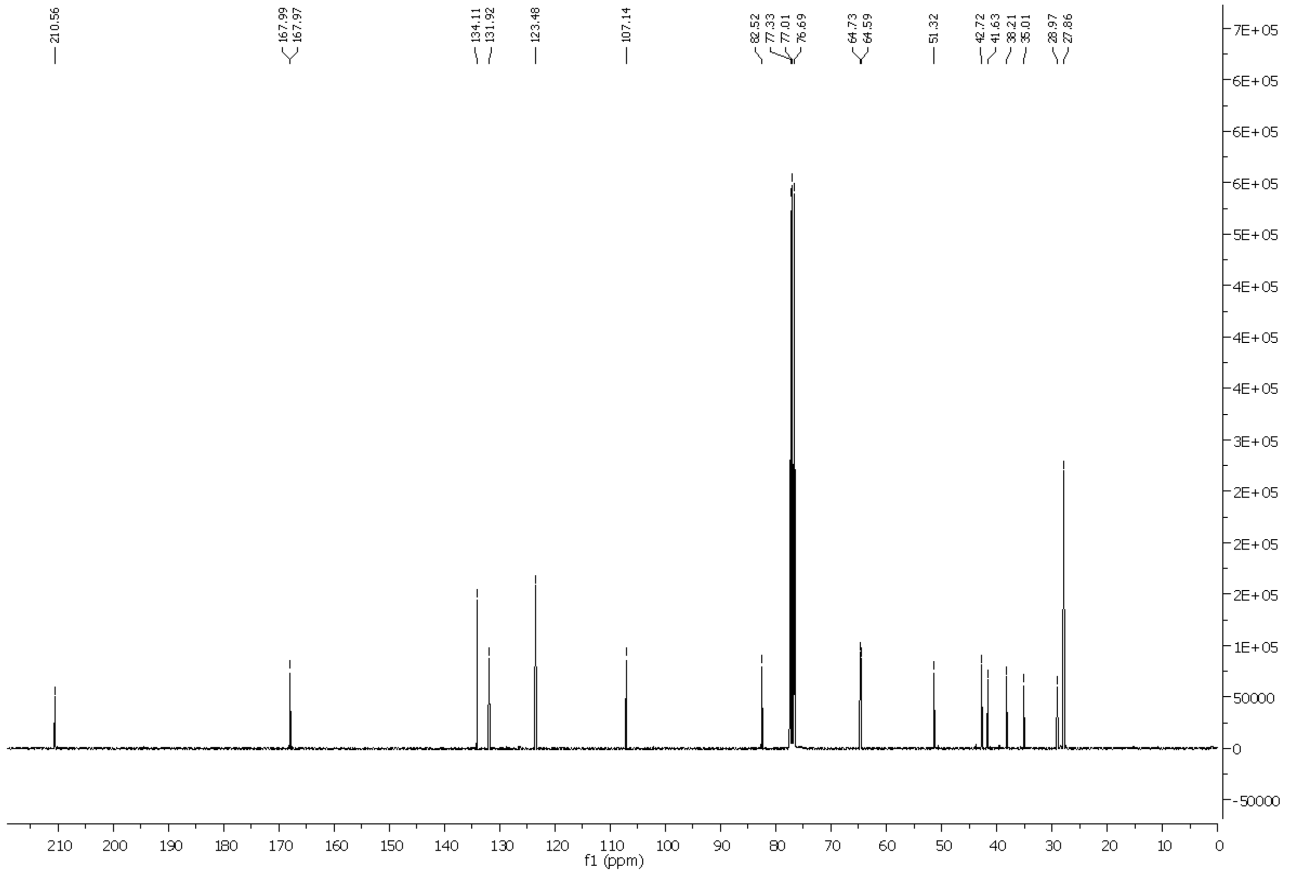
210.56
167.99
167.97
134.11
131.92
123.48
107.14
82.52
77.33
77.01
76.69
64.73
64.59
51.32
42.72
41.63
38.21
35.01
28.97
27.86
f1 (ppm)

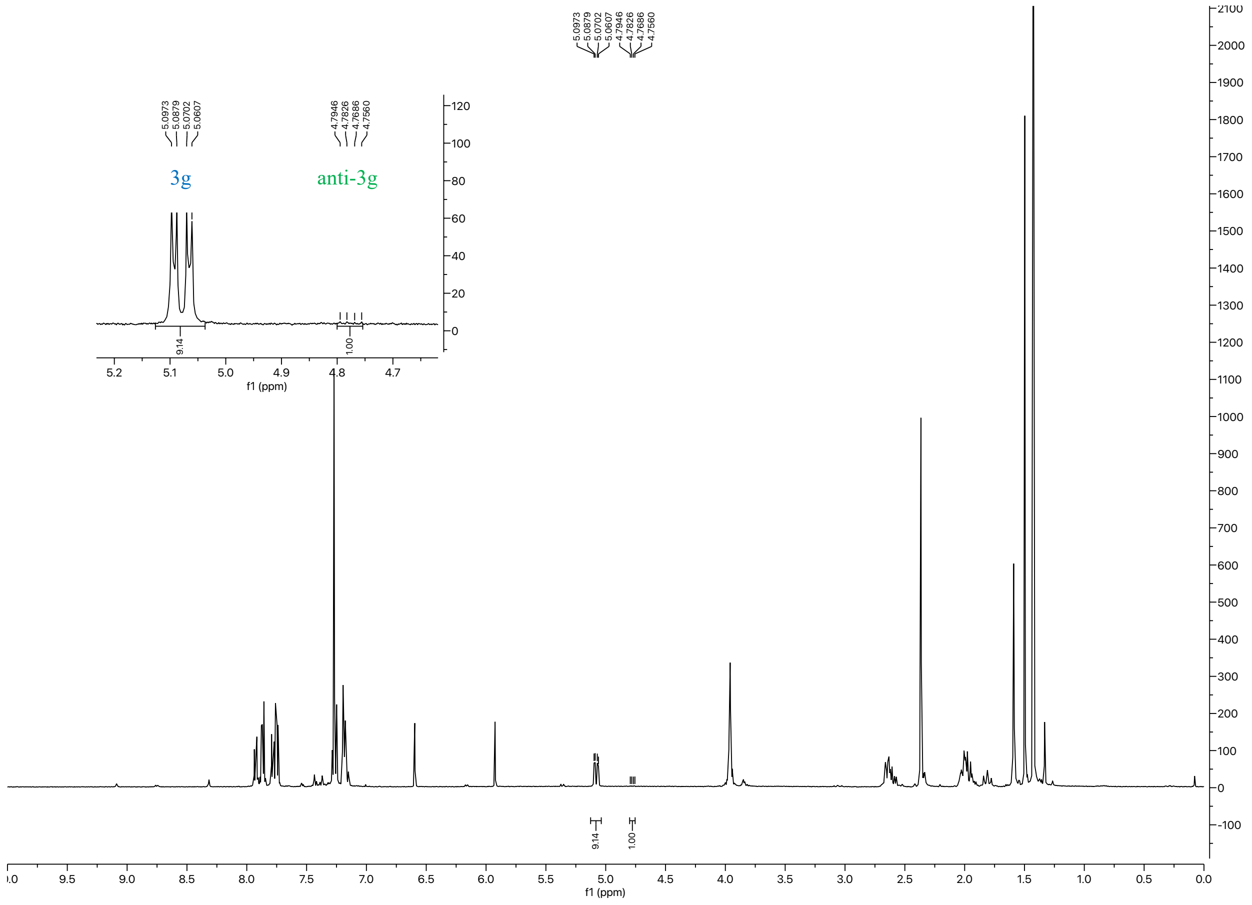
5.0973
5.0879
5.0702
5.0607
4.7946
4.7826
4.7686
4.7560
3g
anti-3g
9.14
1.00
f1 (ppm)

^{1}H NMR, COSY and ^{13}C NMR (2*S*)-*tert*-butyl 2-(1,3-dioxoisoindolin-2-yl)-3-((1*S*)-5-ethyl-2-oxocyclohexyl)propanoate(3h)

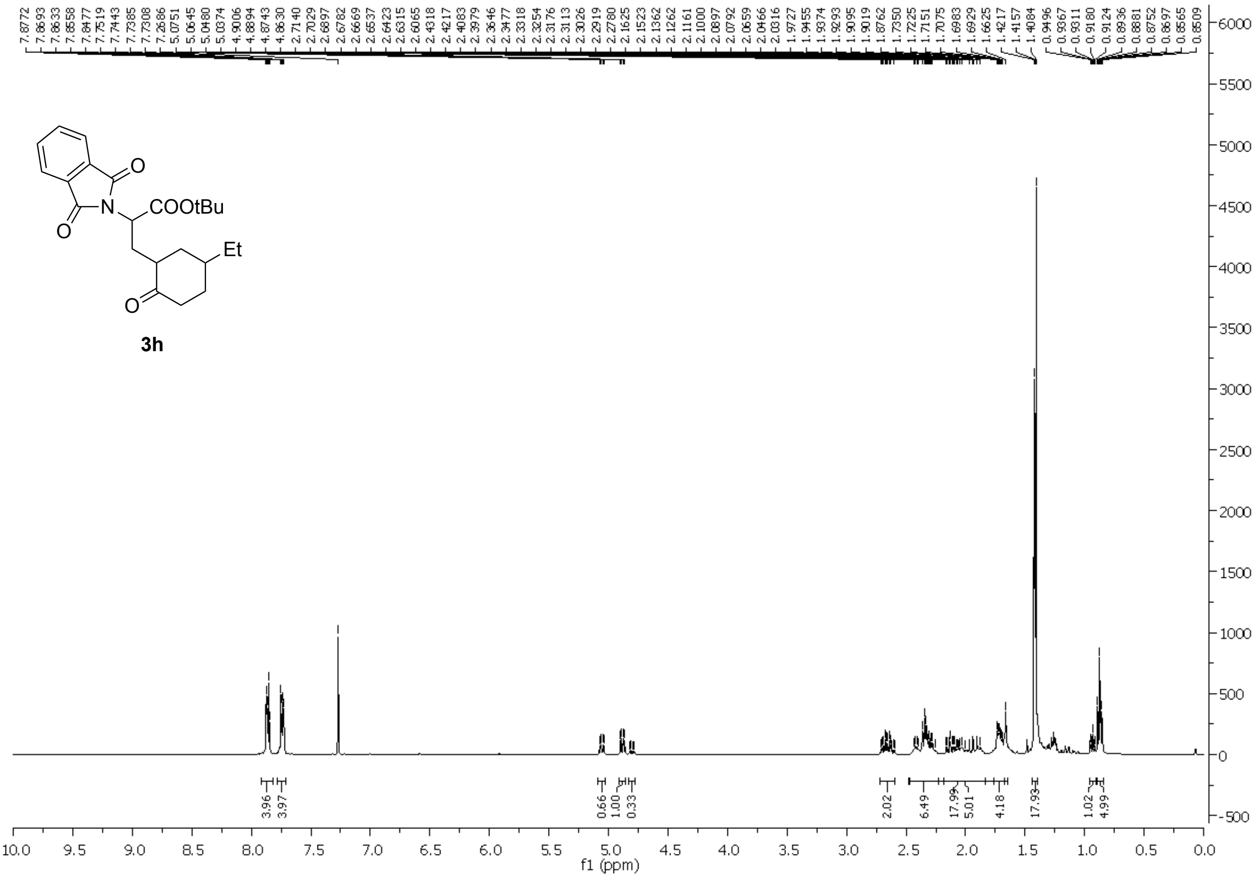

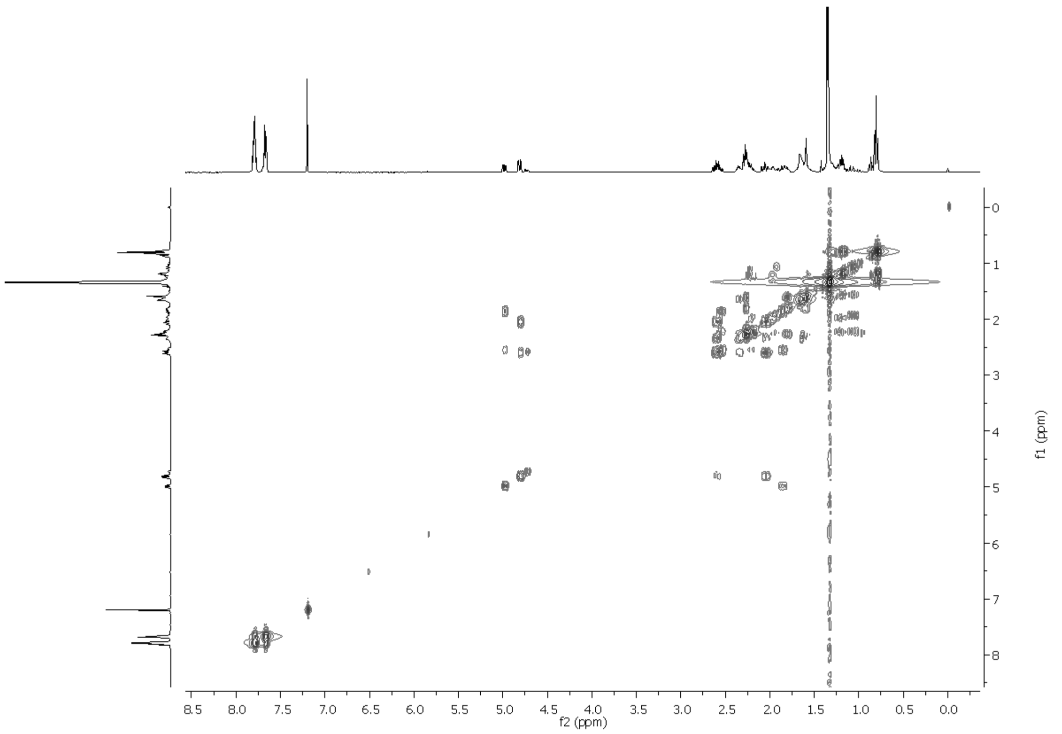

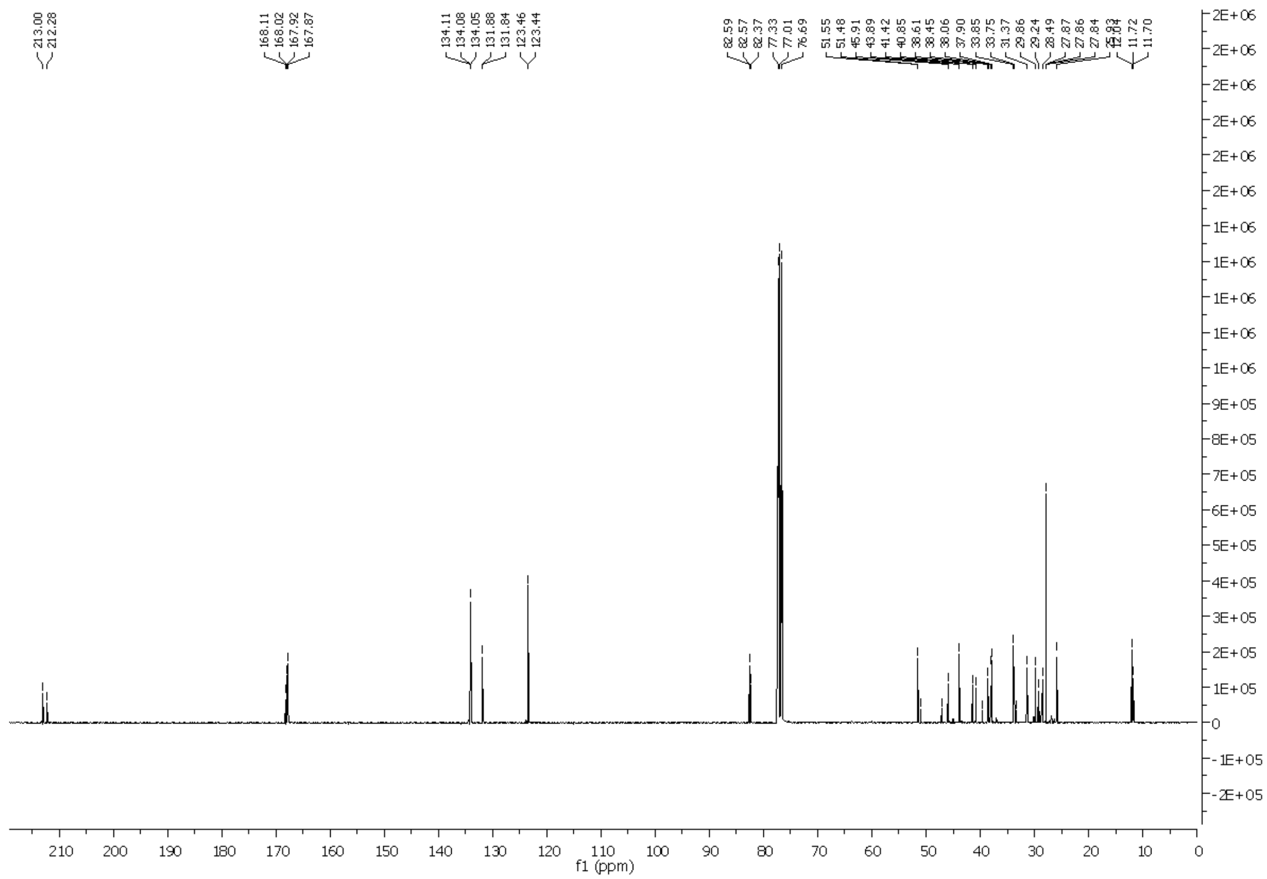

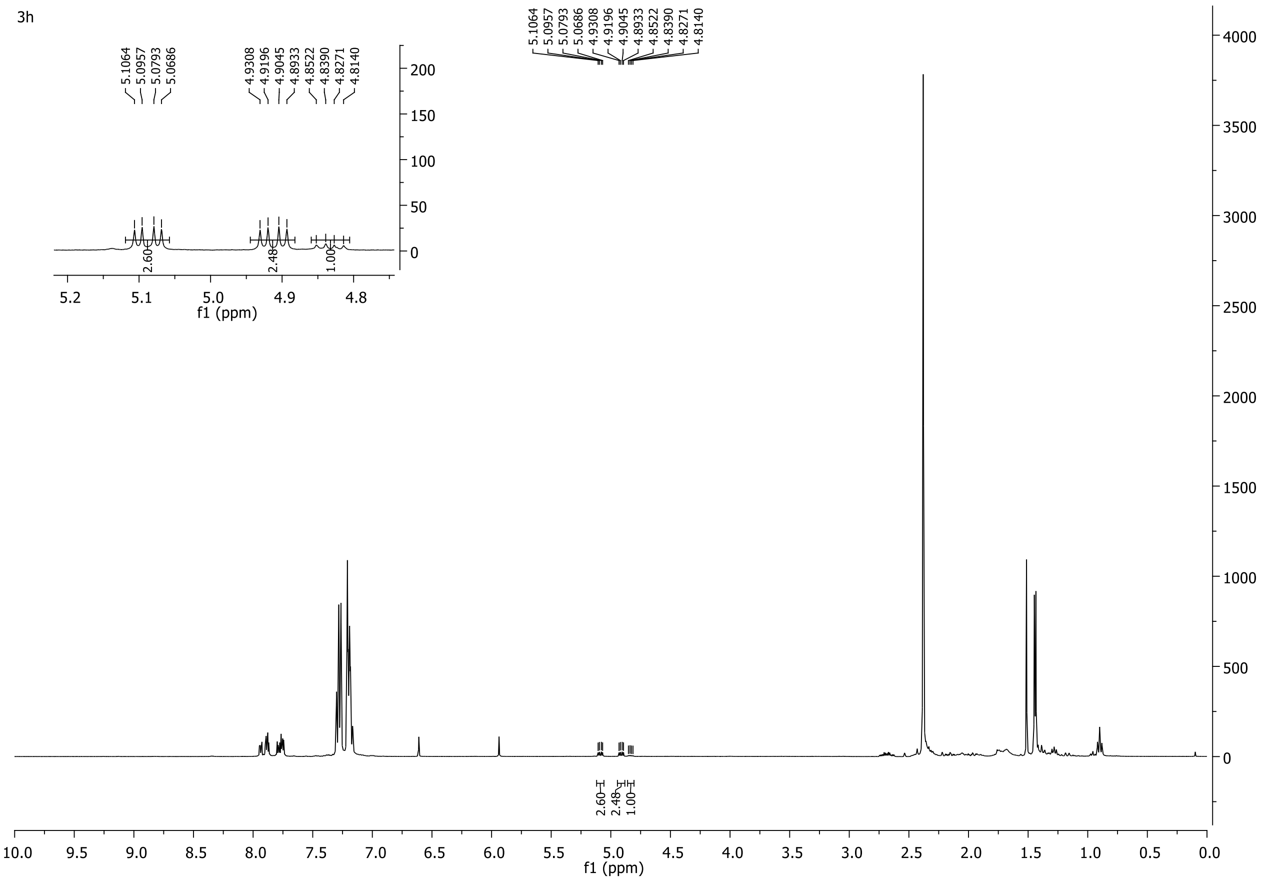
3h

^{1}H NMR, COSY and ^{13}C NMR (*S*)-*tert*-butyl 2-(1,3-dioxoisoindolin-2-yl)-3-((*S*)-3-oxotetrahydro-2*H*-pyran-4-yl)propanoate (3i)

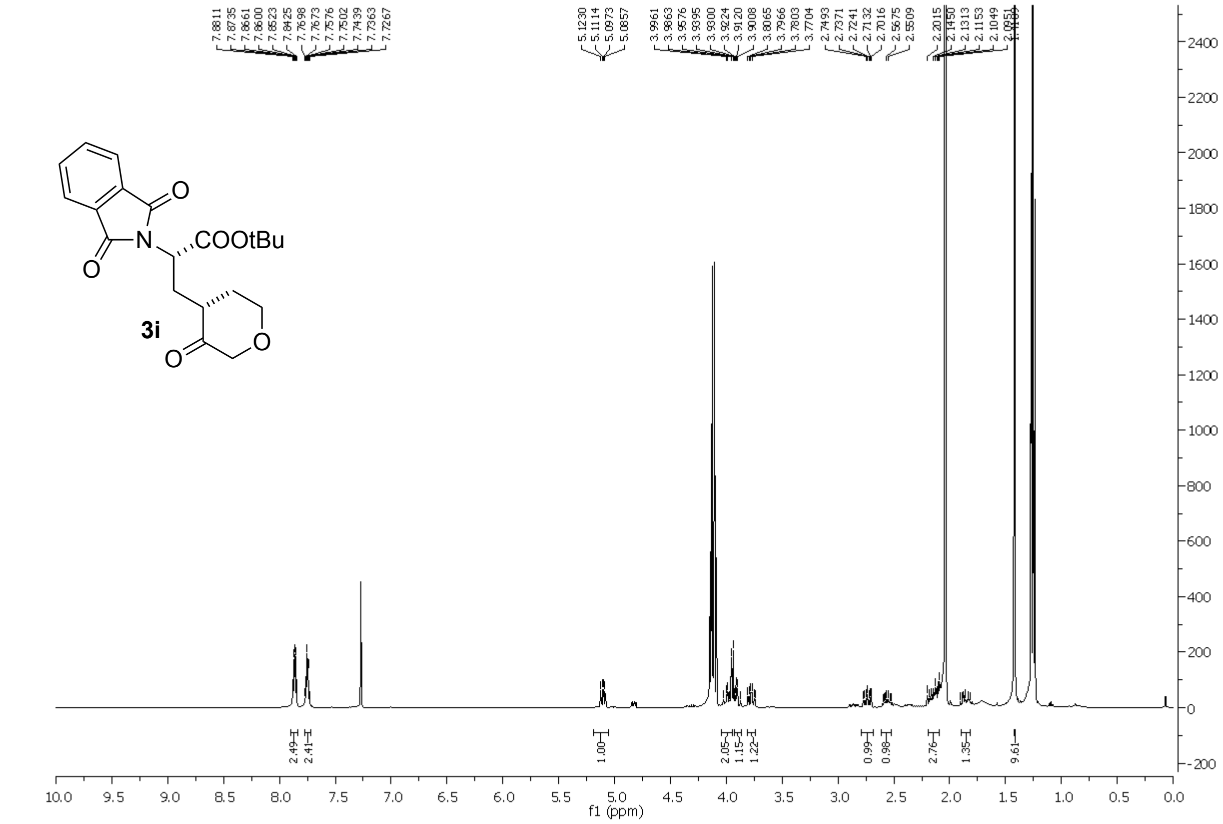

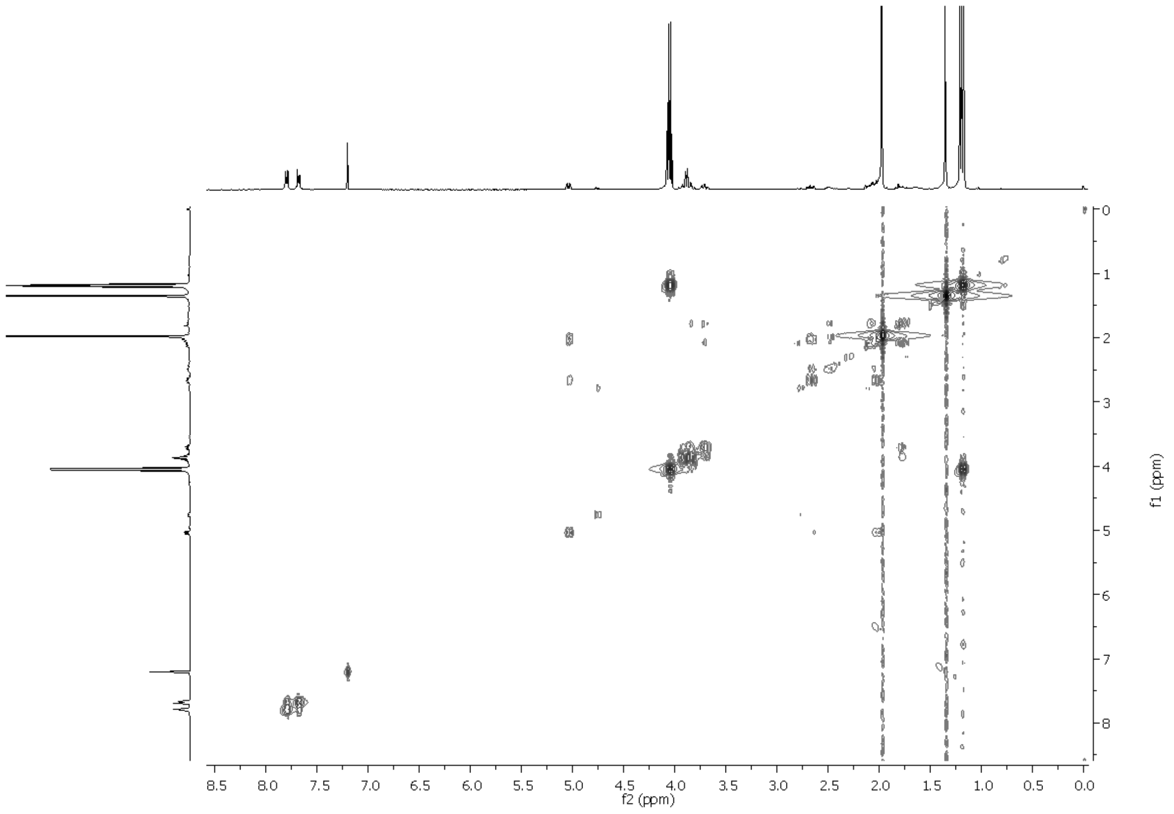

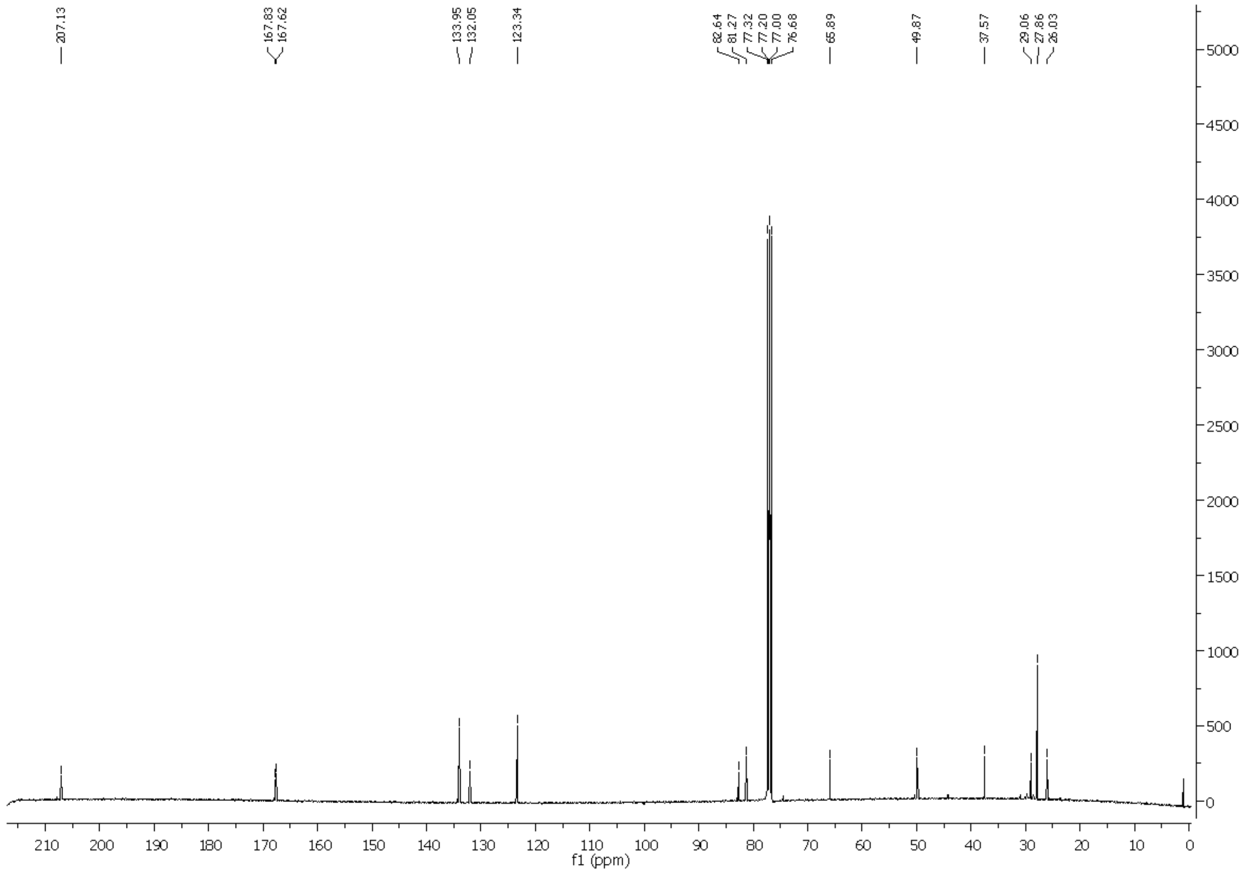
207.13
167.83
167.62
133.95
132.05
123.34
82.64
81.27
77.32
77.20
77.00
76.68
65.89
49.87
37.57
29.06
27.86
26.03
f1 (ppm)

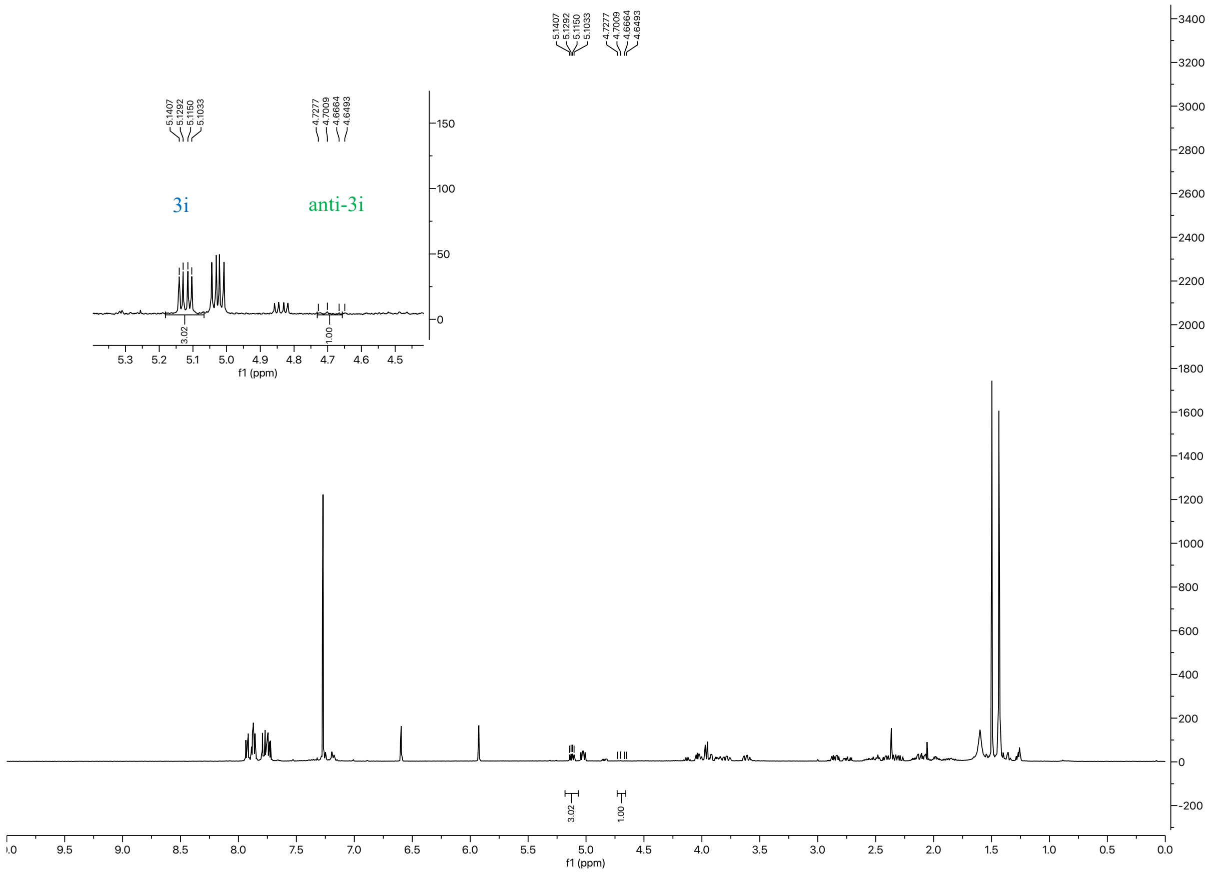
5.1407
5.1292
5.1150
5.1033
4.7277
4.7009
4.6664
4.6493
3i
anti-3i
3.02
1.00
f1 (ppm)

^{1}H NMR, COSY and ^{13}C NMR (*S*)-*tert*-butyl 2-(1,3-dioxoisoindolin-2-yl)-3-((*S*)-3-oxotetrahydro-2*H*-pyran-2-yl)propanoate (3i')

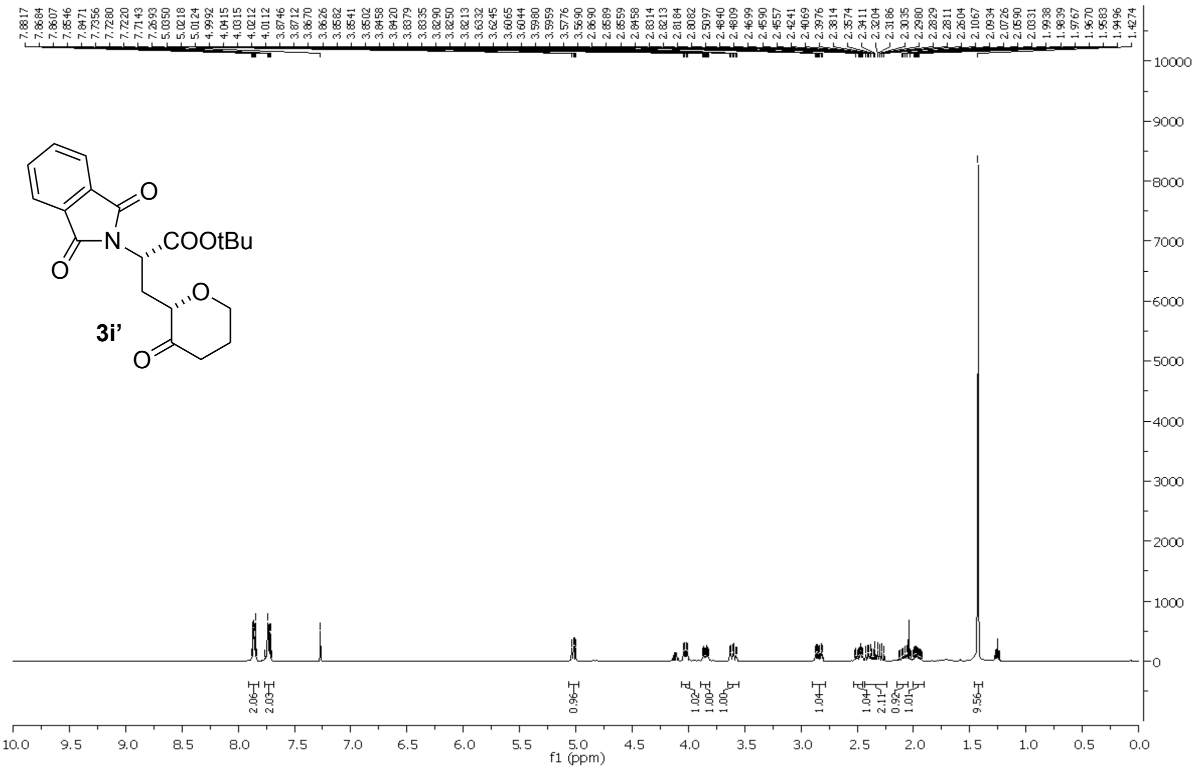

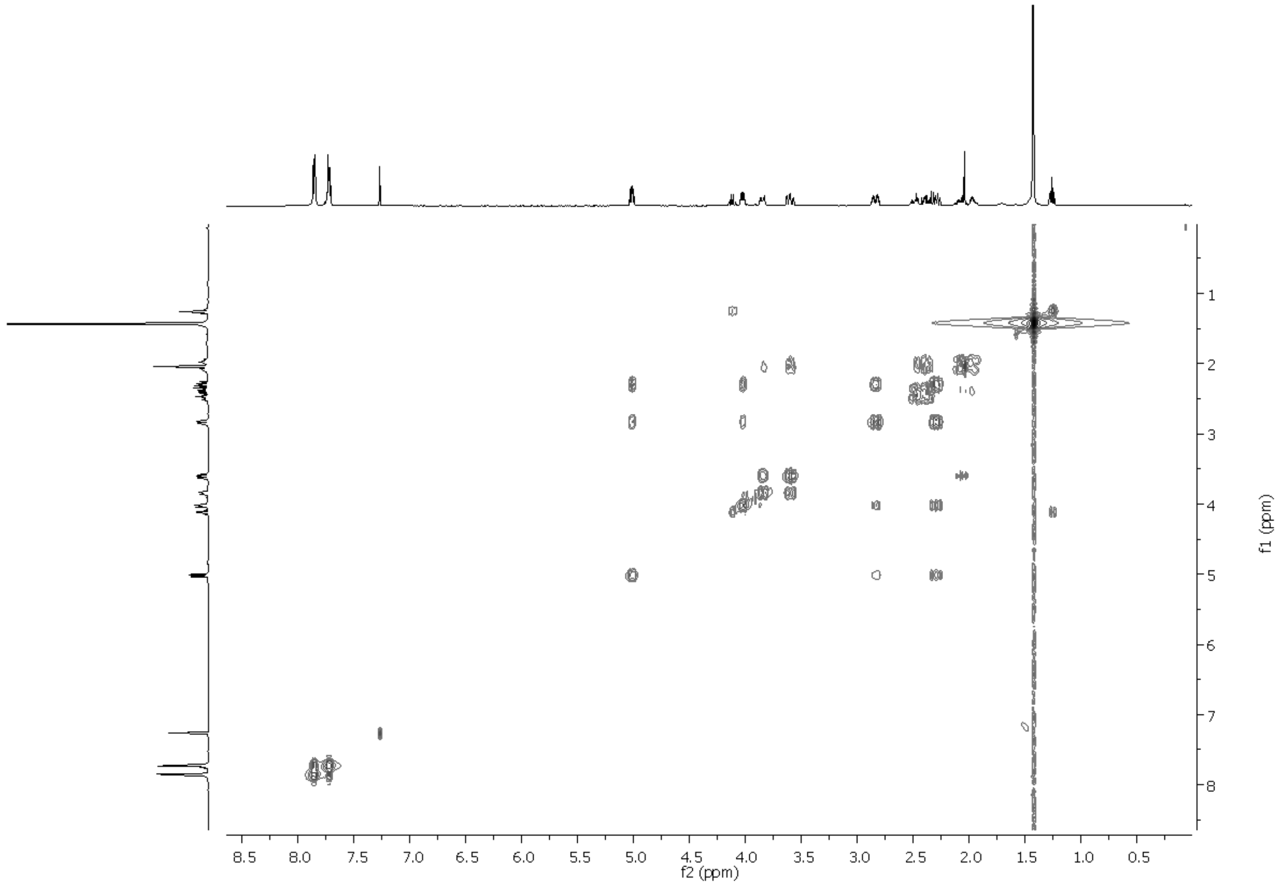

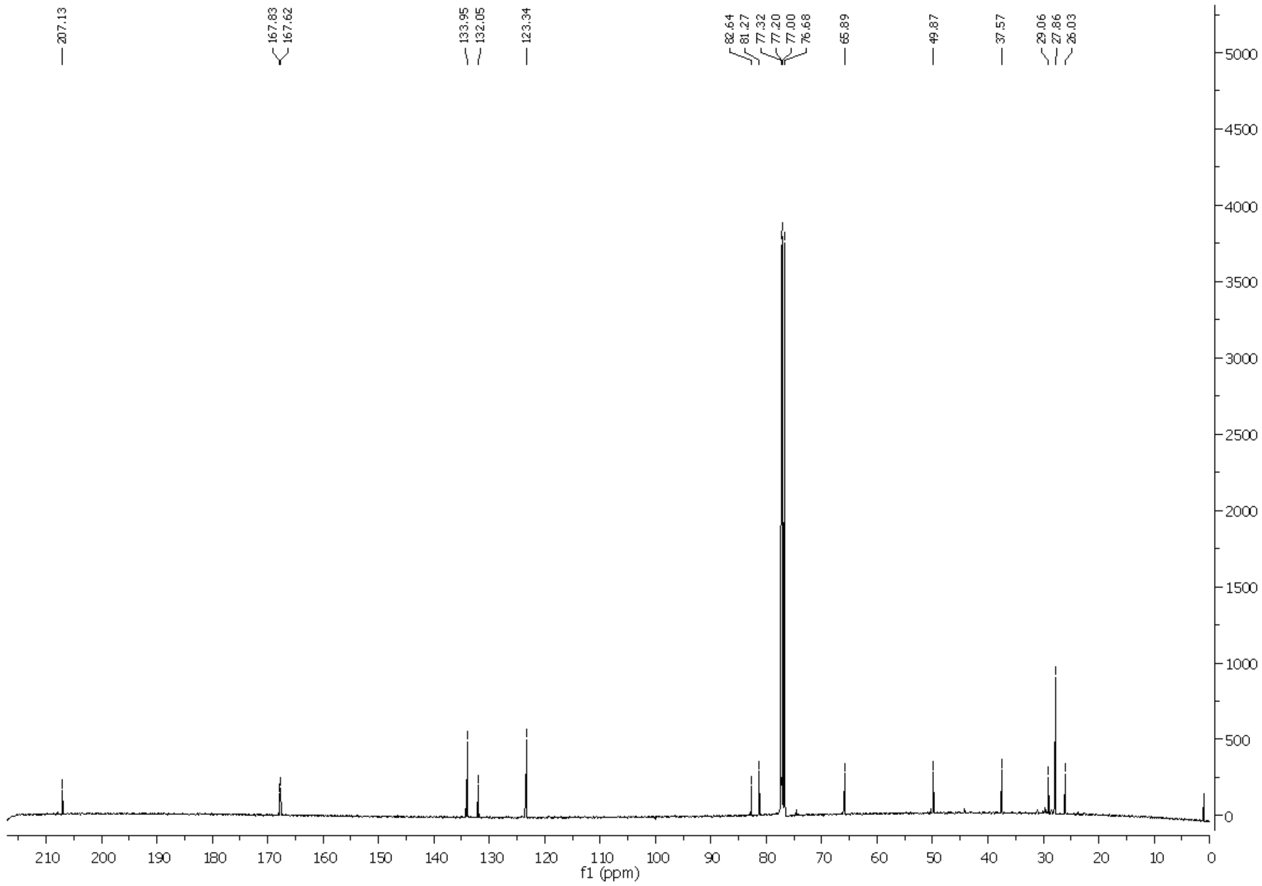
207.13
167.83
167.62
133.95
132.05
123.34
82.64
81.27
77.32
77.20
77.00
76.68
65.89
49.87
37.57
29.06
27.86
26.03
f1 (ppm)

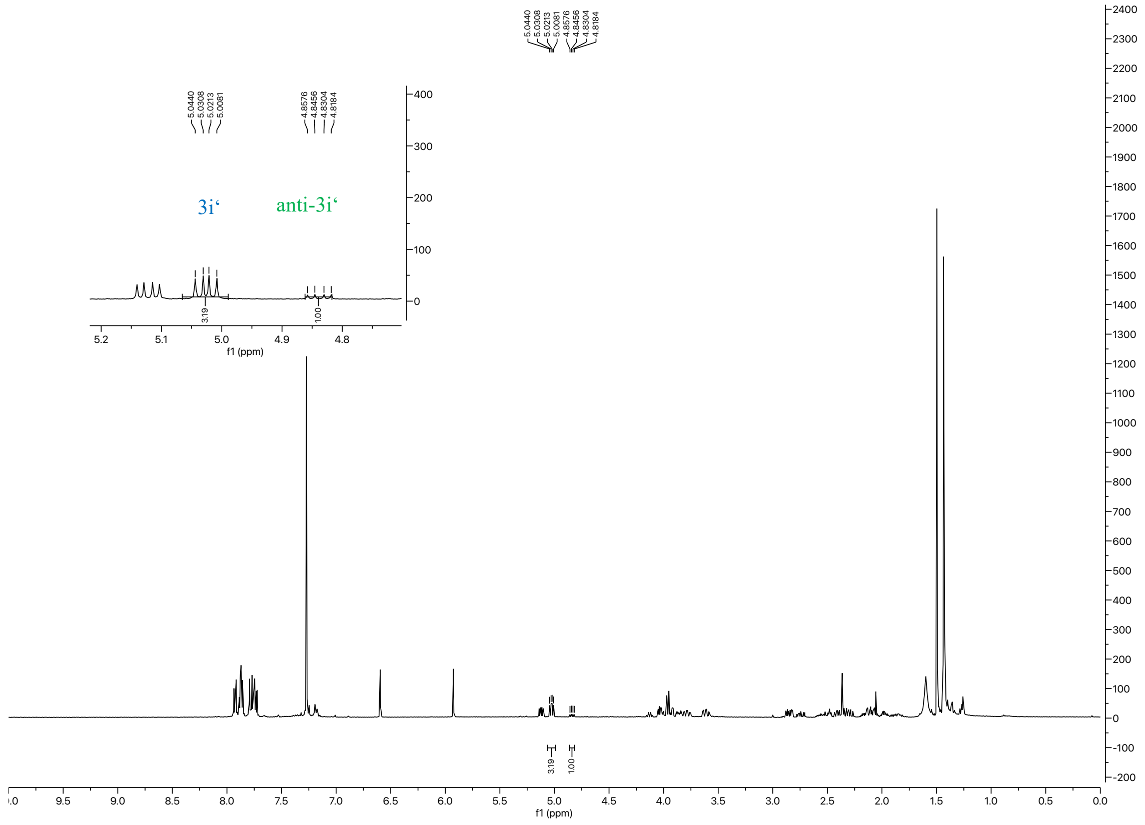
5.0440
5.0308
5.0213
5.0081
4.8576
4.8456
4.8304
4.8184
3i‘
anti-3i‘
3.19
1.00
f1 (ppm)

^{1}H NMR, COSY and ^{13}C NMR of (*S*)-methyl 2-(1,3-dioxoisoindolin-2-yl)-3-((*S*)-2-oxocyclohexyl)propanoate (3j)

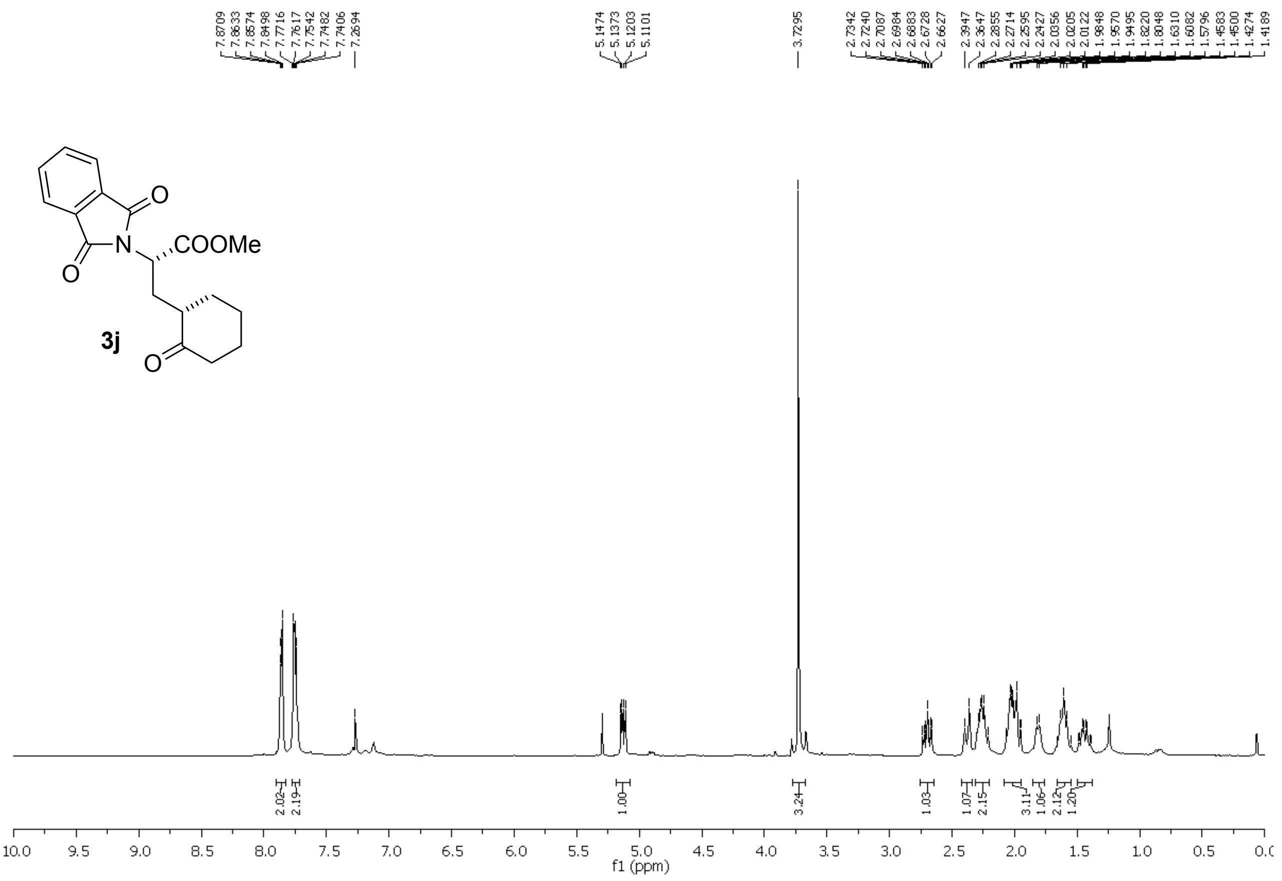

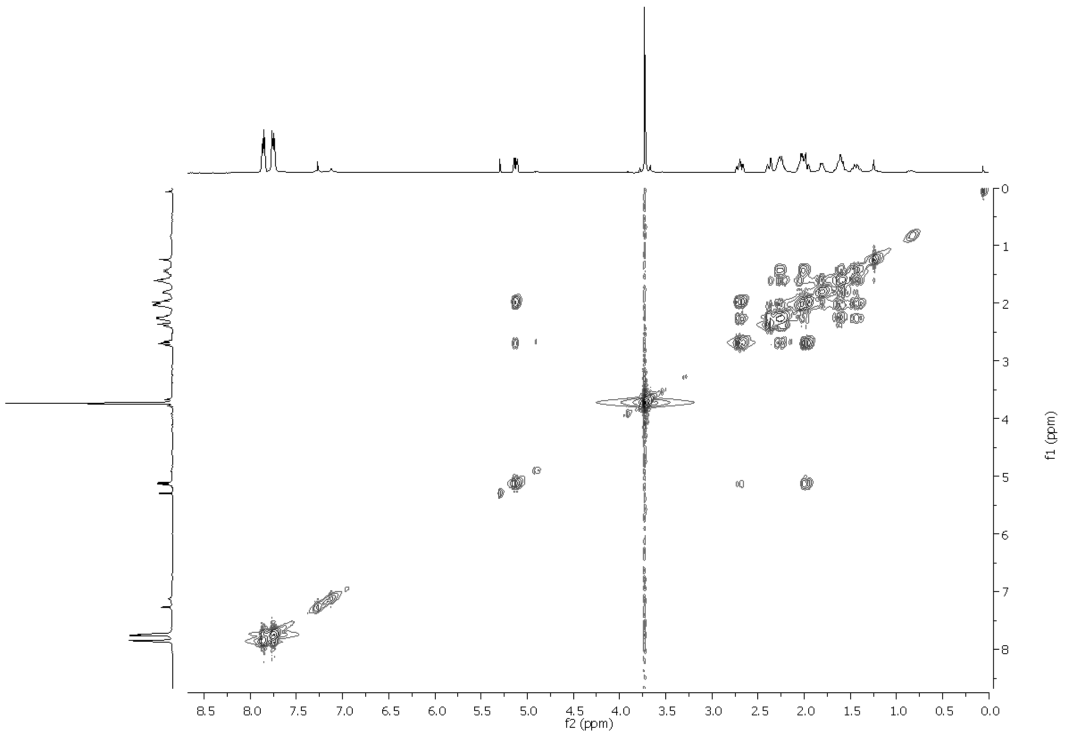

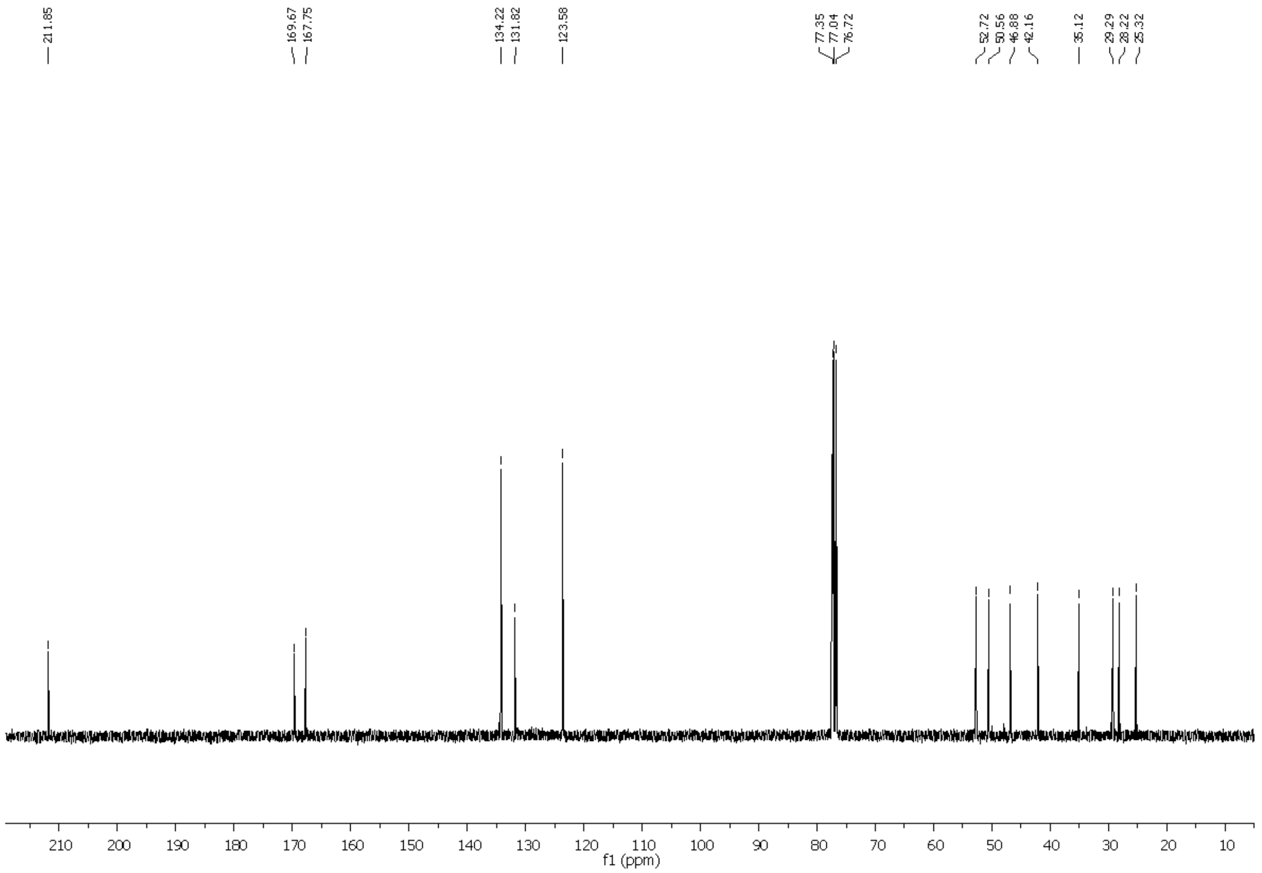
211.85
169.67
167.75
134.22
131.82
123.58
77.35
77.04
76.72
52.72
50.56
46.88
42.16
35.12
29.29
28.22
25.32
f1 (ppm)

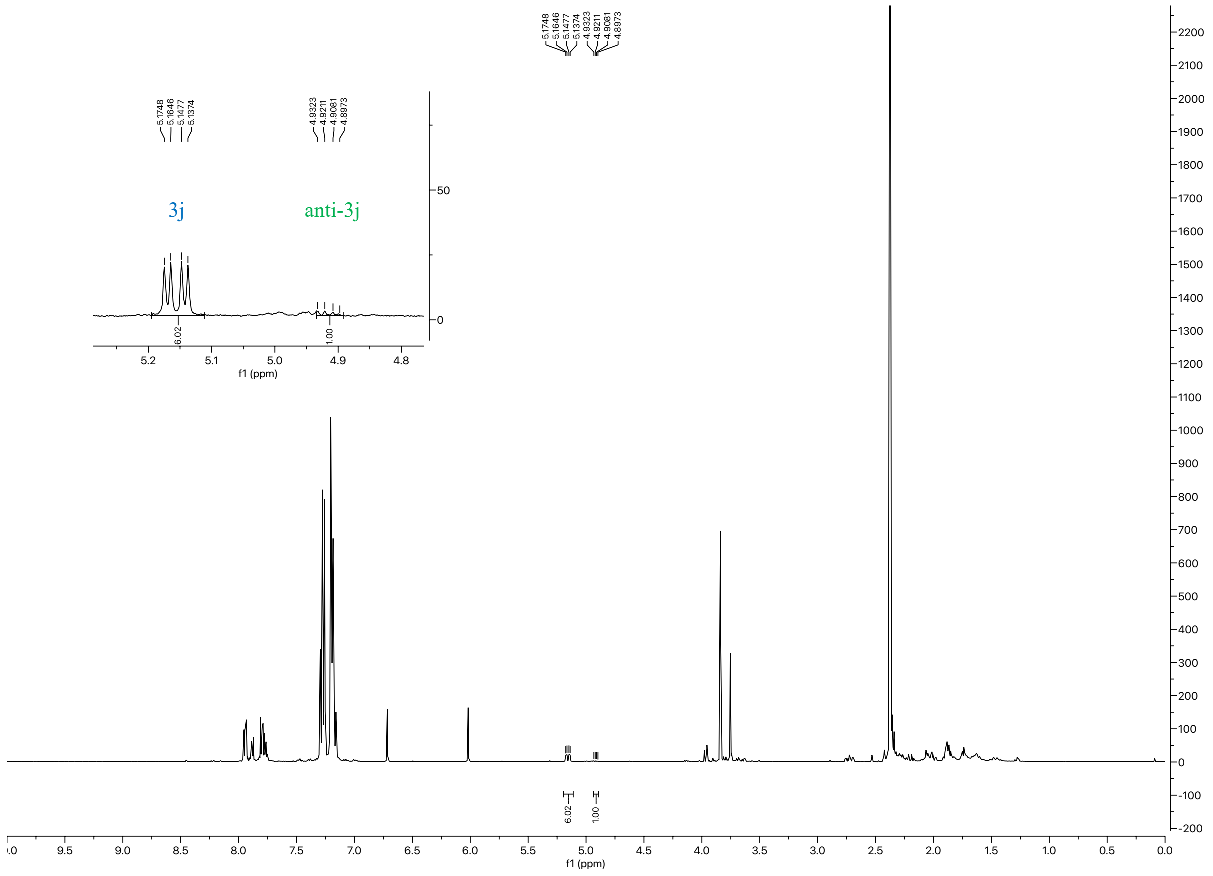
5.1748
5.1646
5.1477
5.1374
4.9323
4.9211
4.9081
4.8973
3j
anti-3j
6.02
1.00
f1 (ppm)

^{1}H-NMR, COSY, NOESY and ^{13}C-NMR of (2*S*,3a*S*)-*tert*-butyl 3,3a,4,5,6,7-hexahydro-2*H*-indole-2-carboxylate (4):

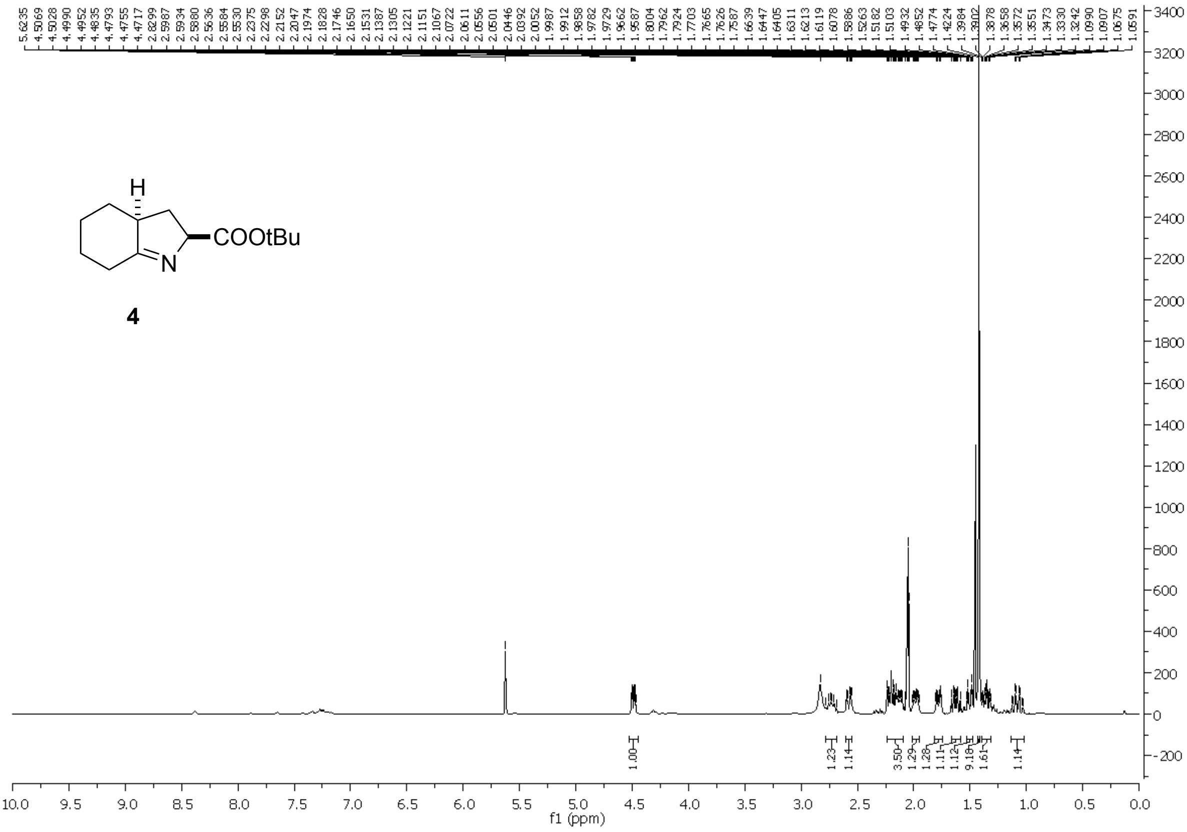

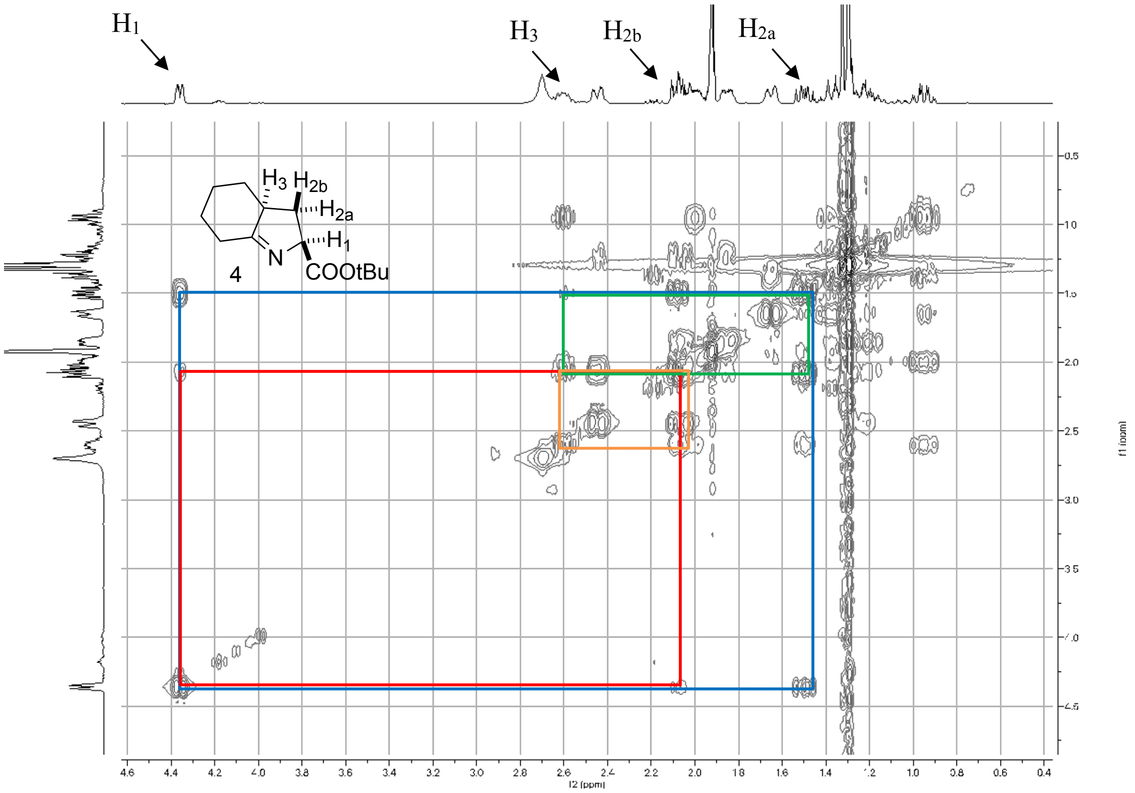

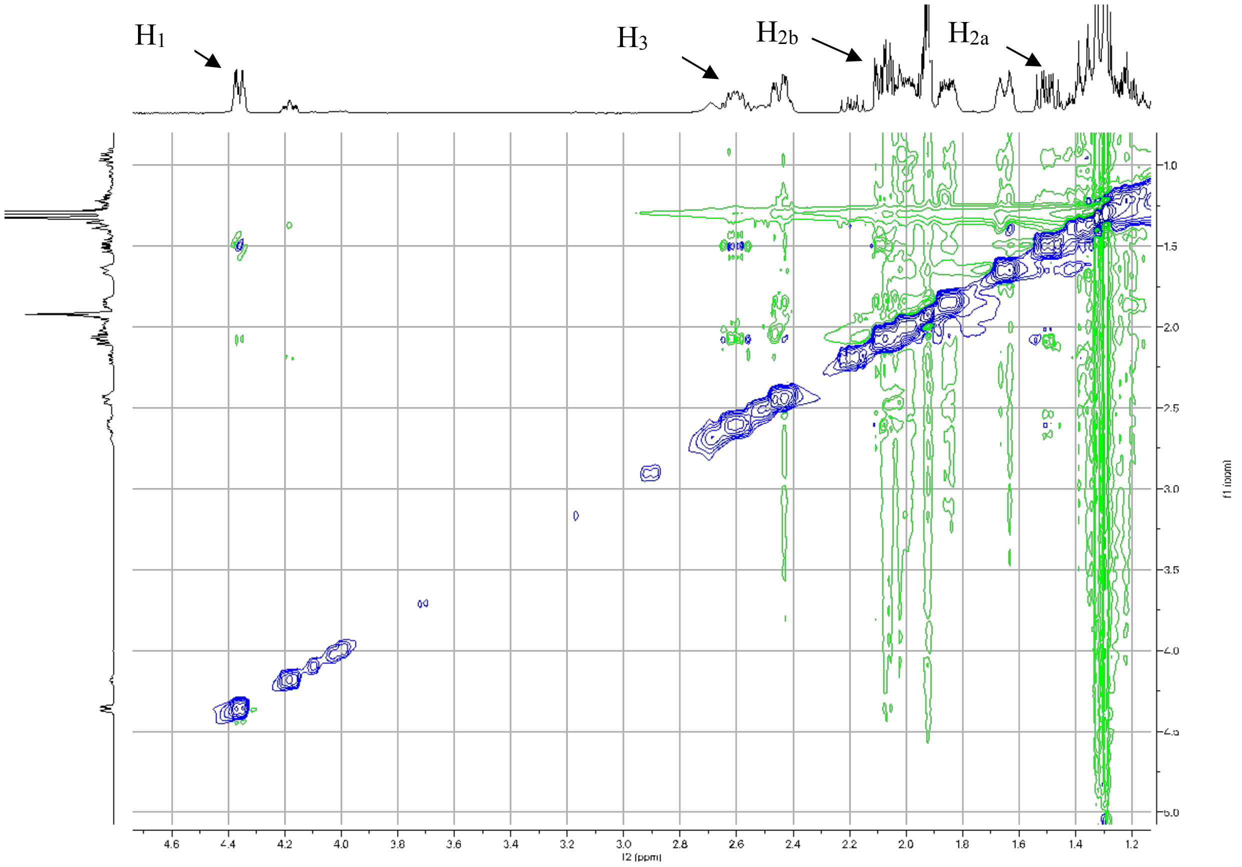

Selected nOe correlations:

- H_1 (4.36 ppm) and H_{2a} (1.51 ppm)
- H_3 (2.60) and H_{2a} (1.51 ppm)

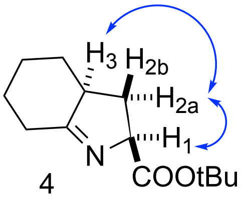

4

The relative stereochemical relationship of the two stereocenters of **4** was assigned by Overhauser enhancement experiments (NOESY) due to the presence of a nOe correlation between H_1 and H_{2a} and a nOe correlation between H_{2a} and H_3, suggesting that the two hydrogen of the stereocenters are located on the same side of the ring.

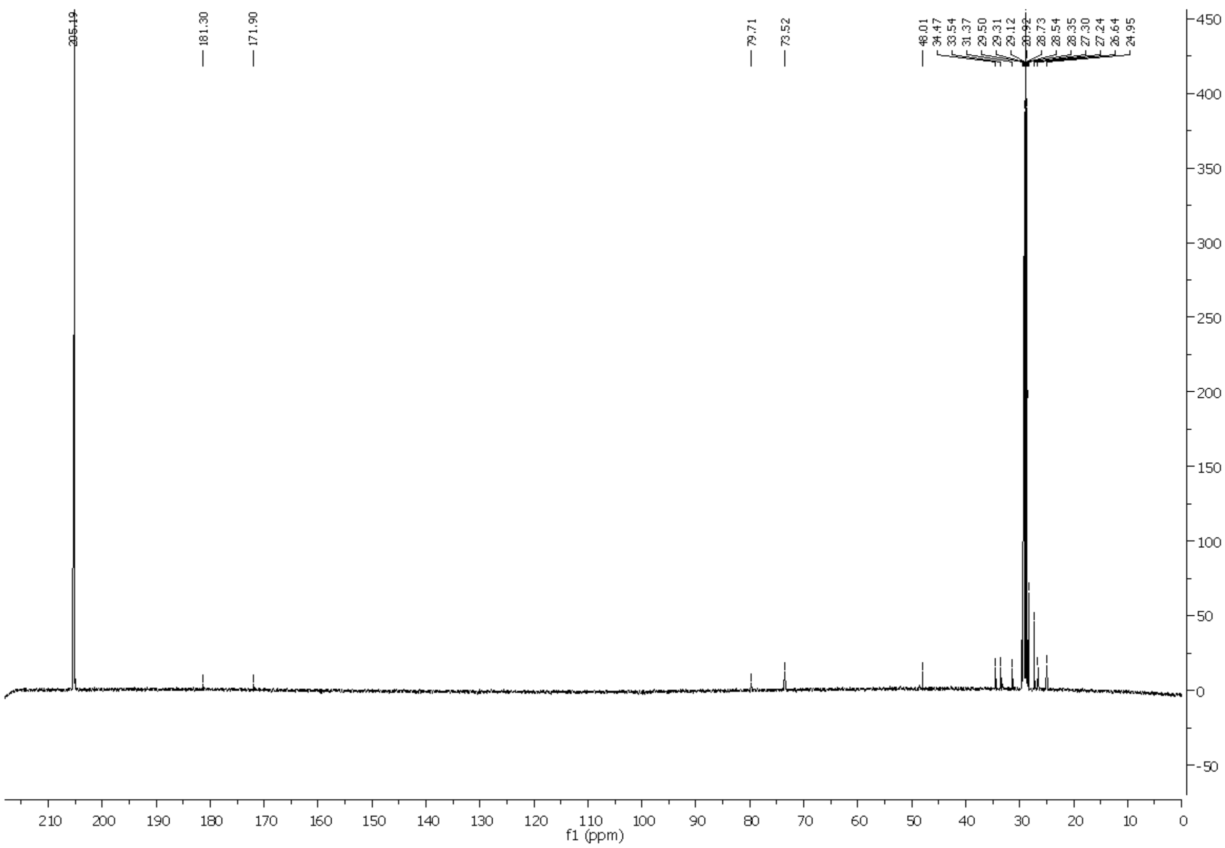

^{1}H-NMR, COSY and ^{13}C-NMR of (2*S*,3*aS*,7*aS*)-*tert*-butyl octahydro-1*H*-indole-2-carboxylate (5):

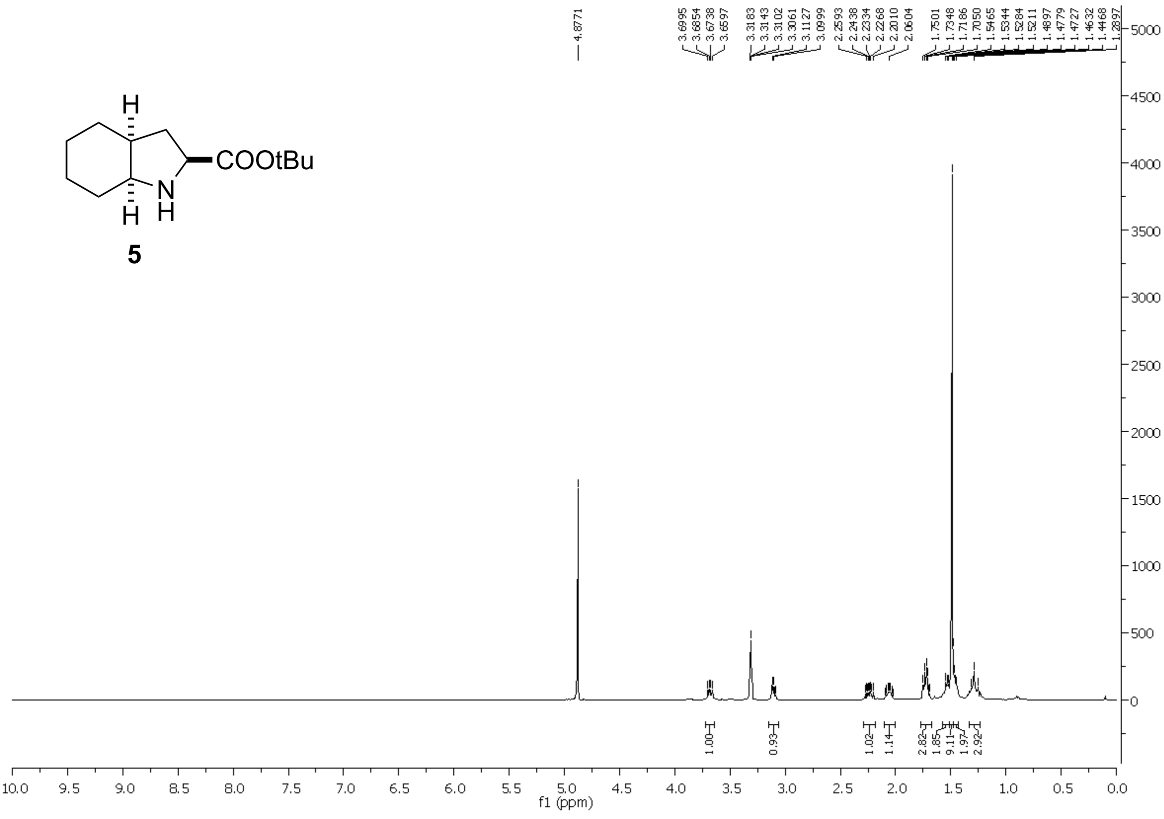

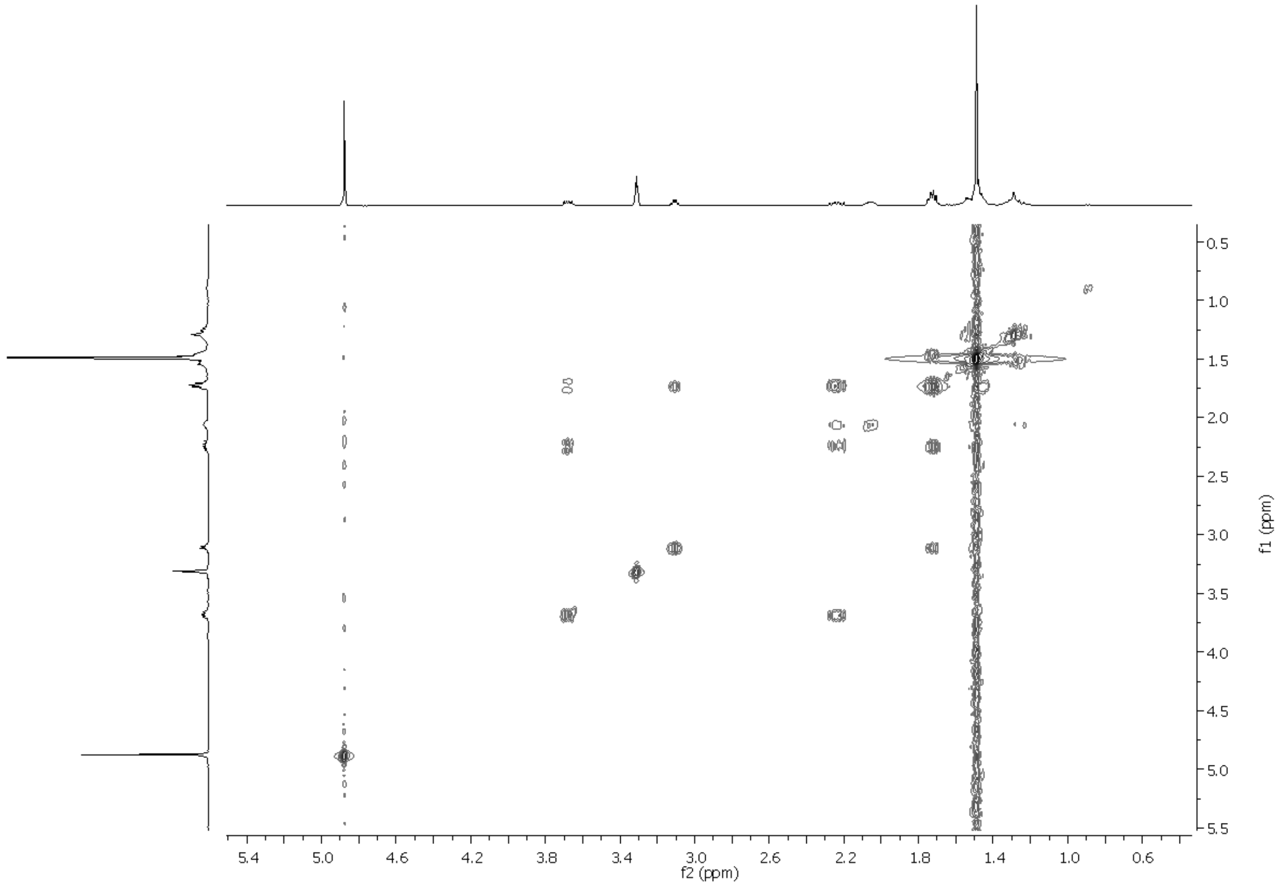
5.4
5.0
4.6
4.2
3.8
3.4
3.0
2.6
2.2
1.8
1.4
1.0
0.6
f2 (ppm)
f1 (ppm)

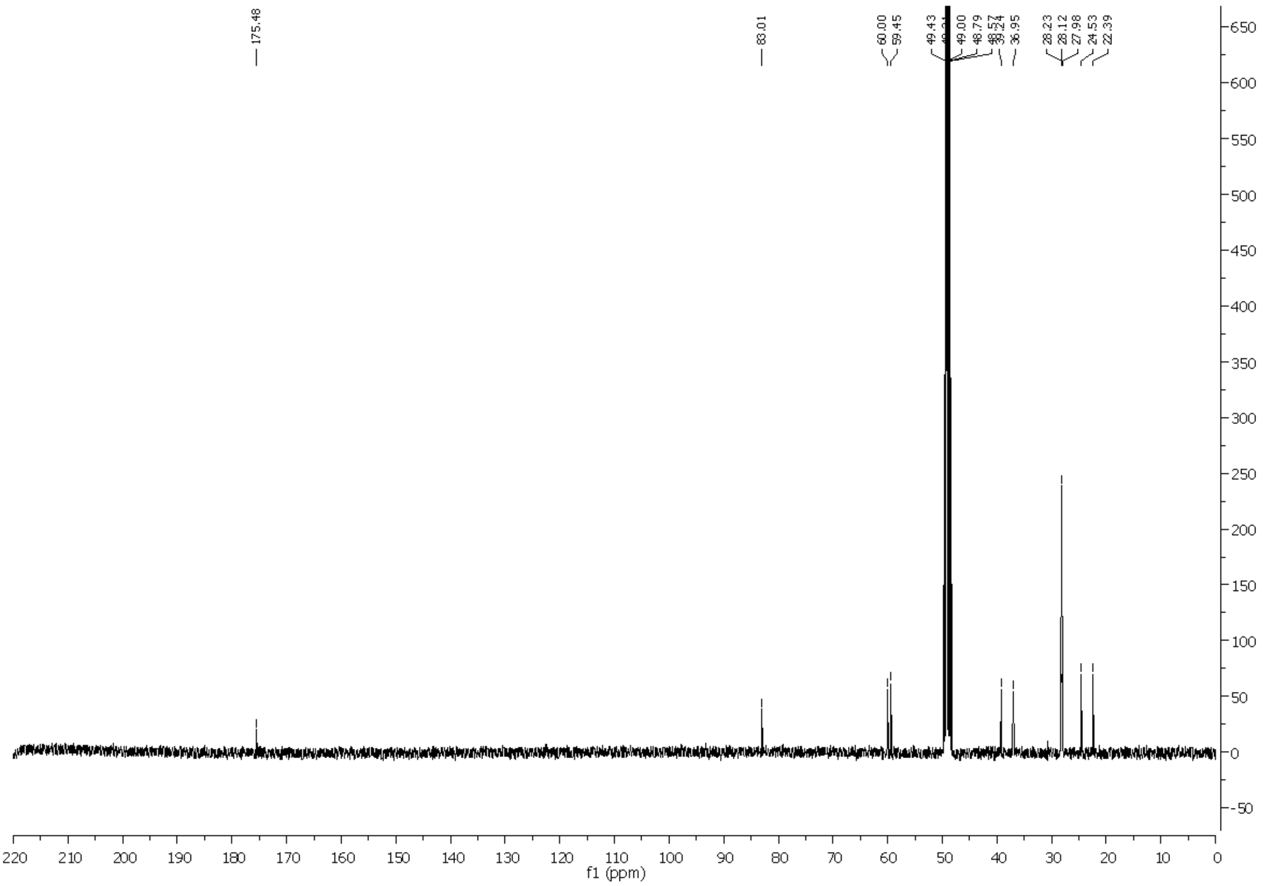
175.48
83.01
60.00
59.45
49.43
49.00
48.79
48.57
39.24
36.95
28.23
28.12
27.98
24.53
22.39
f1 (ppm)

^{1}H NMR, COSY and ^{13}C NMR of (*S*)-*tert*-butyl 2-(1,3-dioxoisoindolin-2-yl)-3-((*S*)-7-oxooxepan-2-yl)propanoate (6)

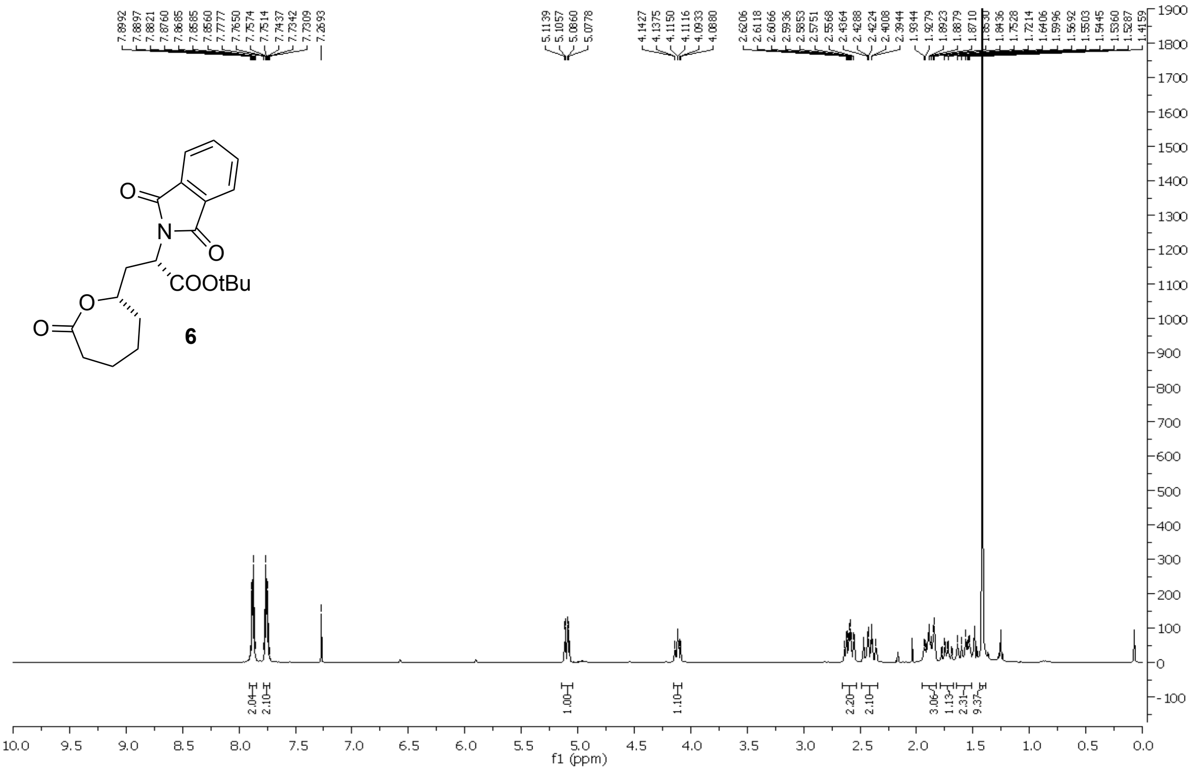

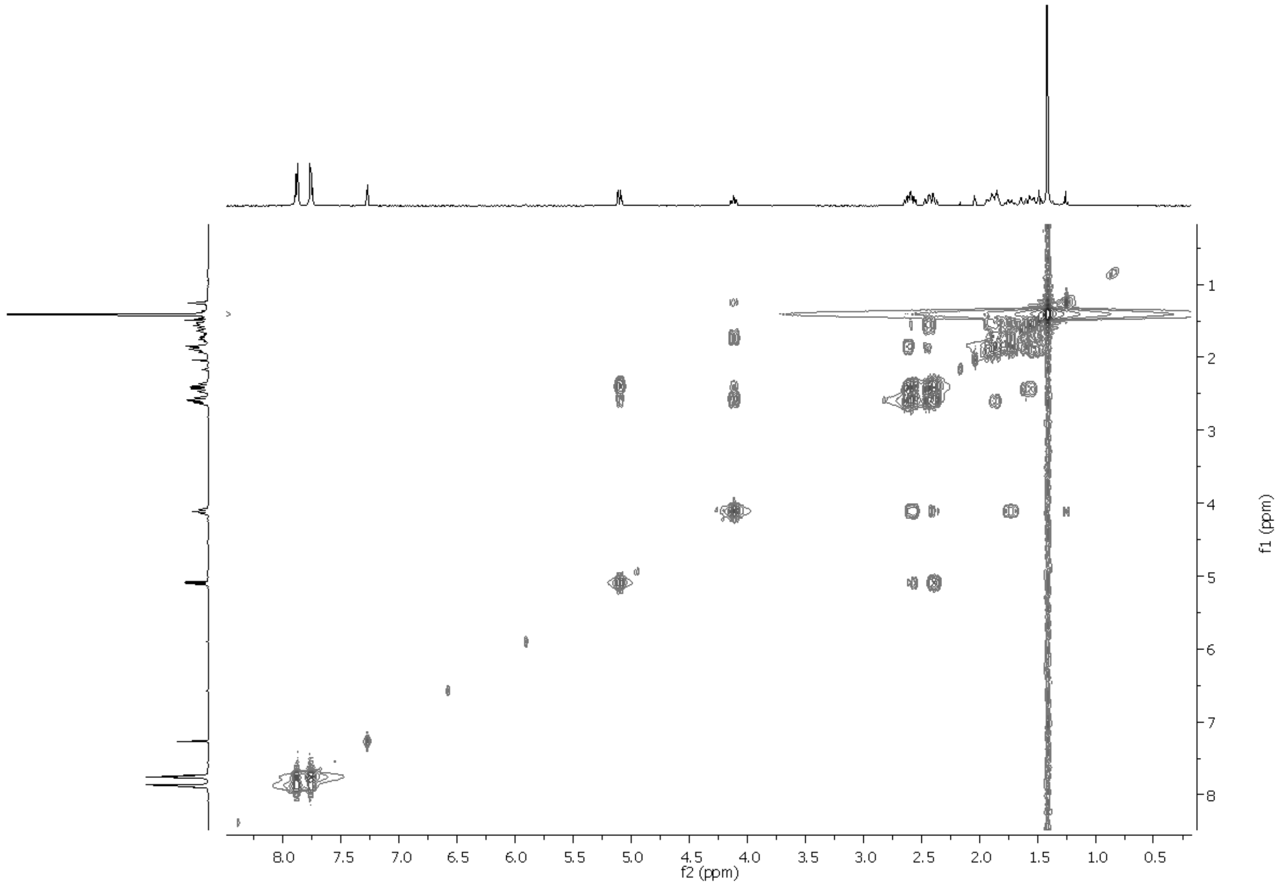

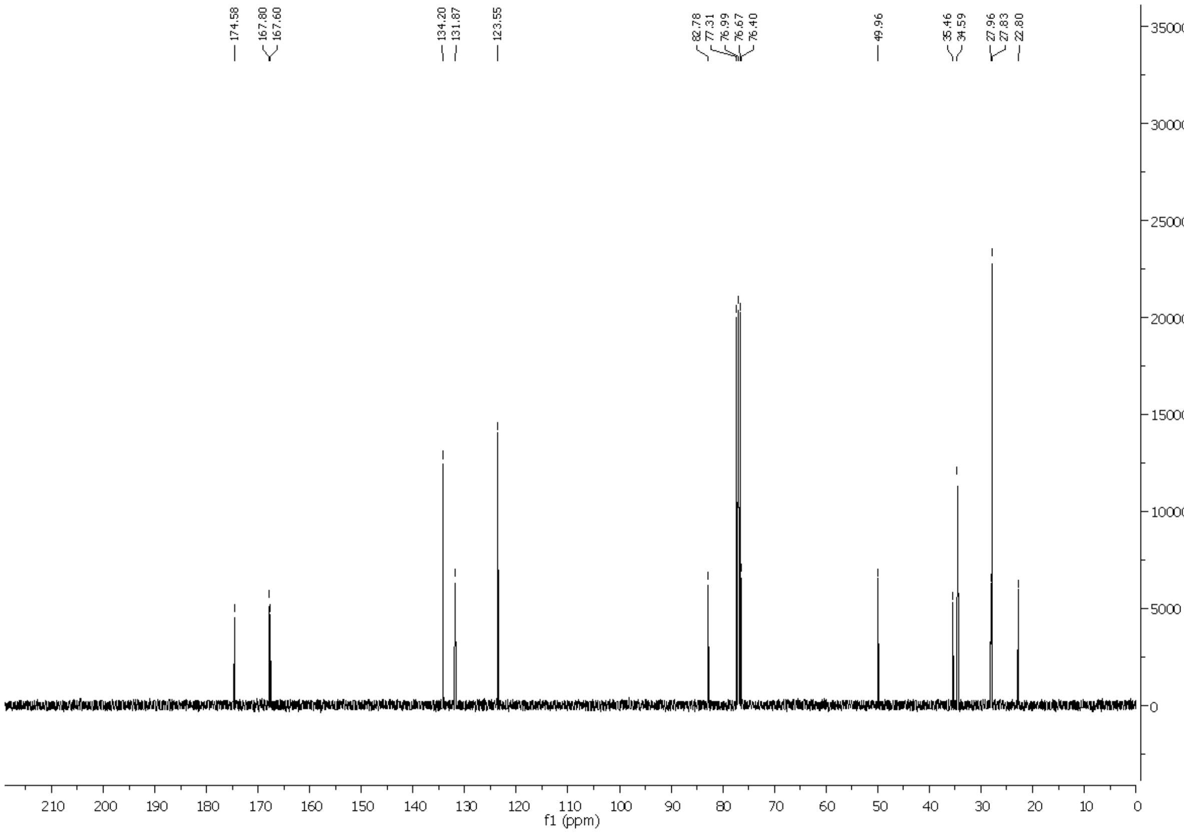

^{1}H-NMR, COSY and ^{13}C-NMR of ((*S*)-*tert*-butyl 2-(1,3-dioxoisoindolin-2-yl)-3-((*S*,*E*)-2-(2-tosylhydrazono)cyclohexyl)propanoate (7):

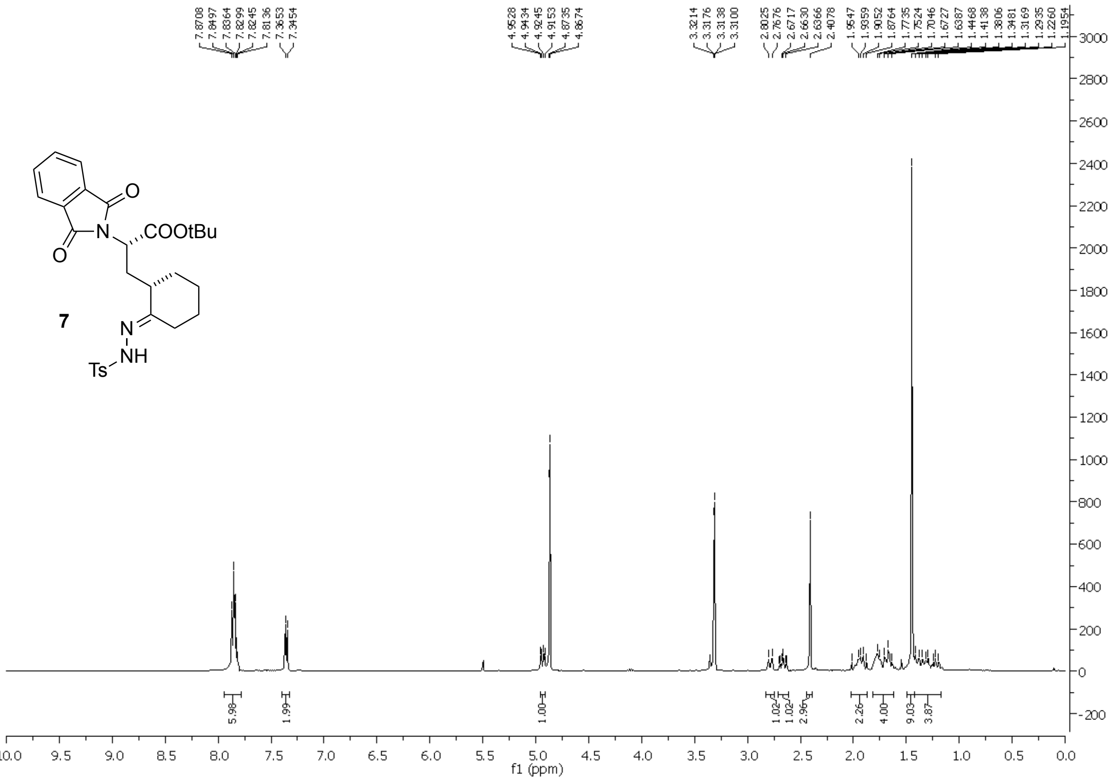

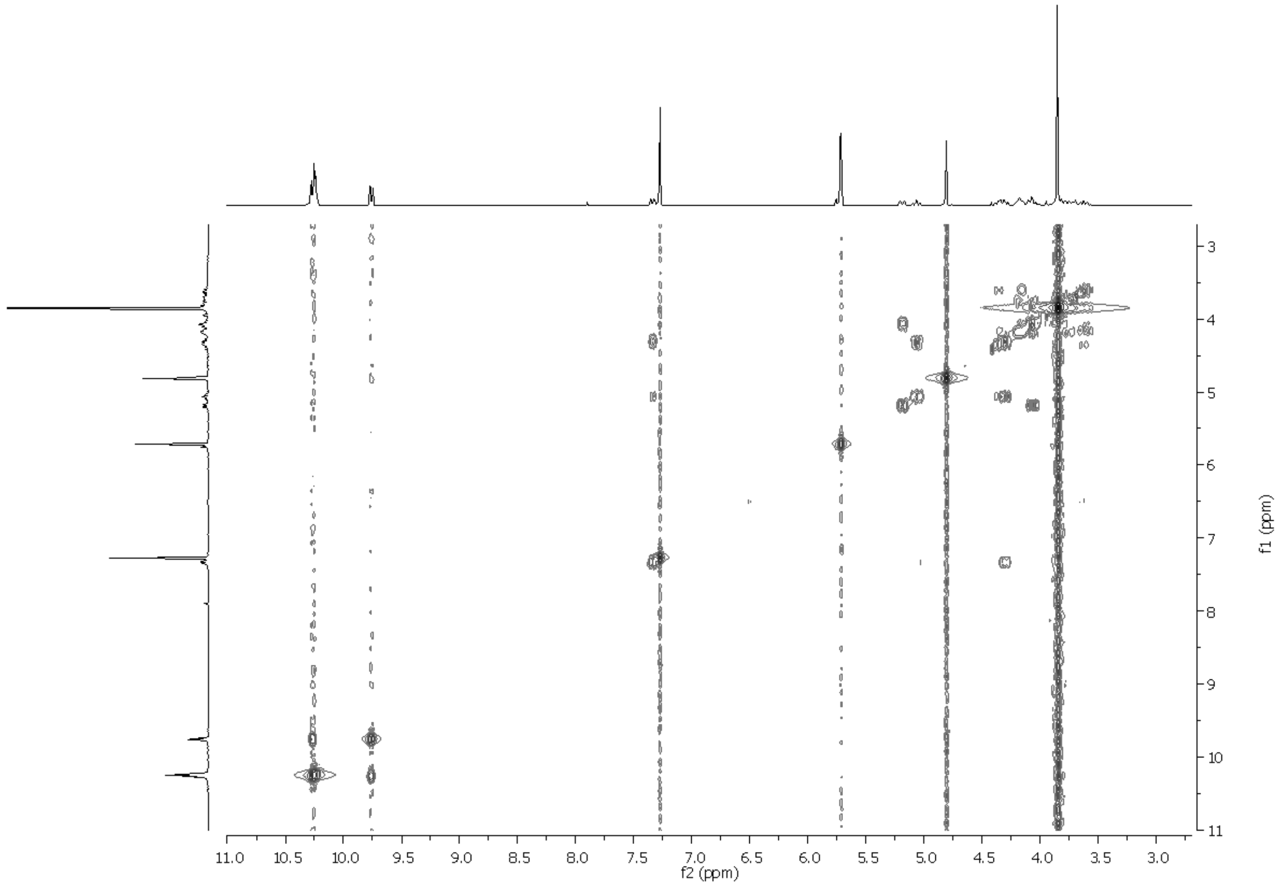
f2 (ppm)
f1 (ppm)

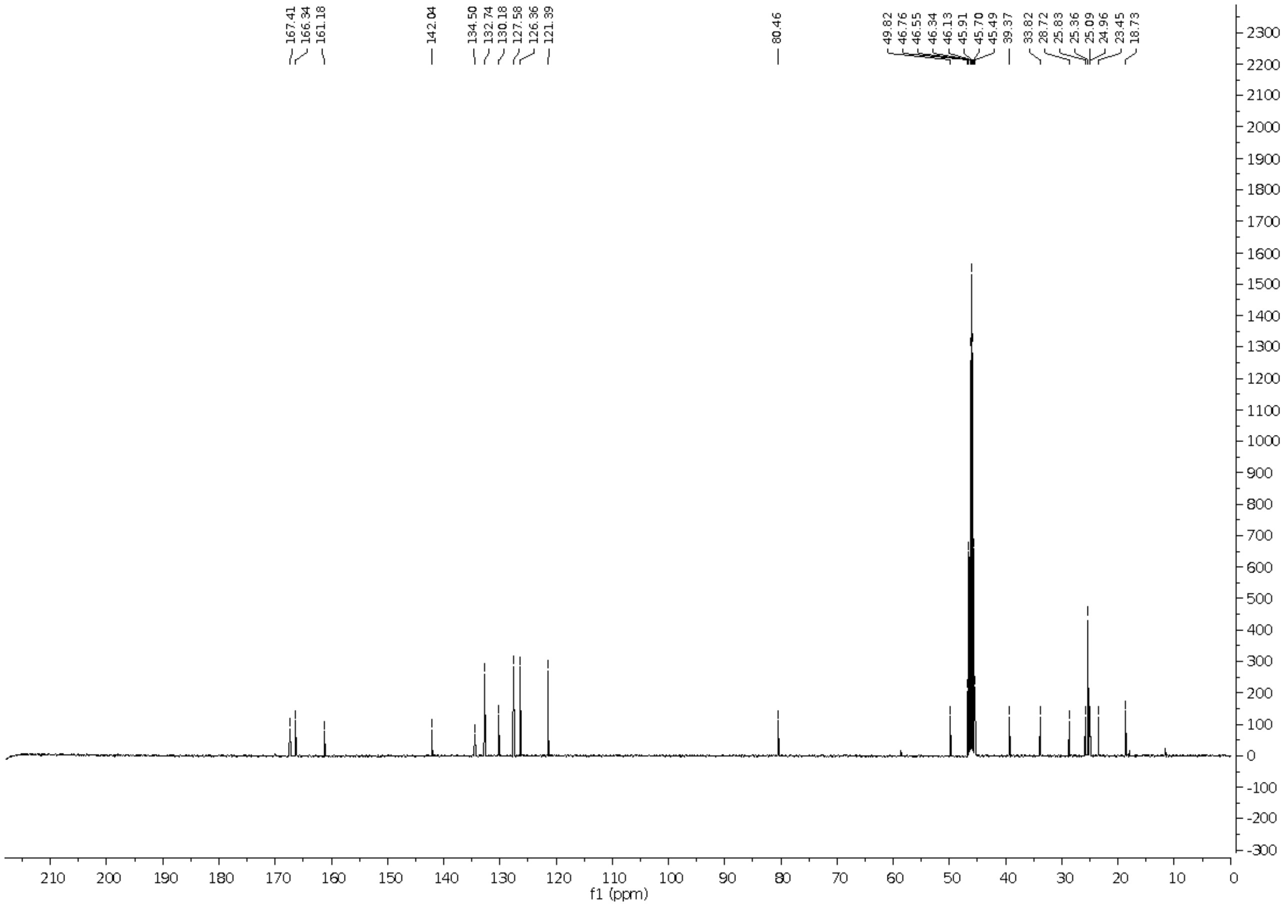
167.41
166.34
161.18
142.04
134.50
132.74
130.18
127.58
126.36
121.39
80.46
49.82
46.76
46.55
46.34
46.13
45.91
45.70
45.49
39.37
33.82
28.72
25.83
25.36
25.09
24.96
23.45
18.73
f1 (ppm)

^{1}H-NMR, COSY and ^{13}C-NMR of (*S*)-*tert*-butyl 2-(1,3-dioxoisoindolin-2-yl)-3-((*S*)-2,3,4,9-tetrahydro-1*H*-carbazol-1-yl)propanoate (8)

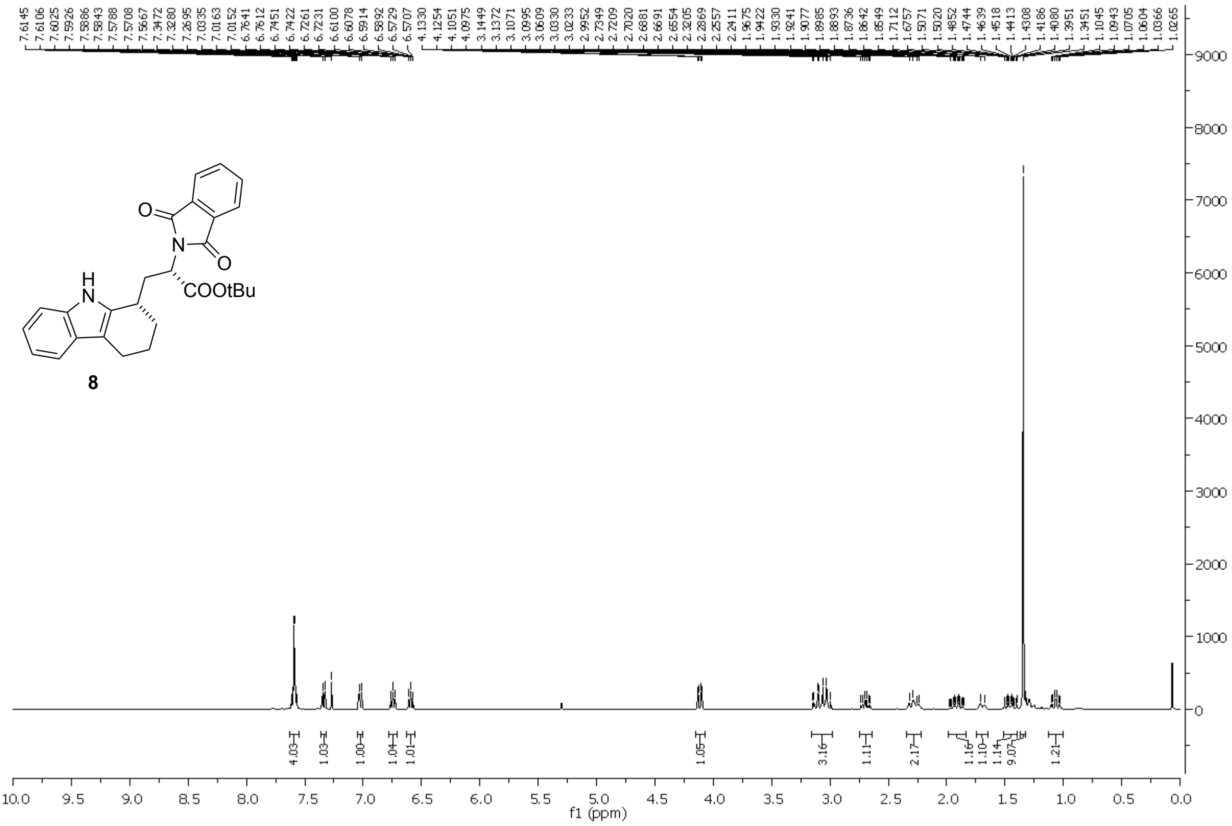

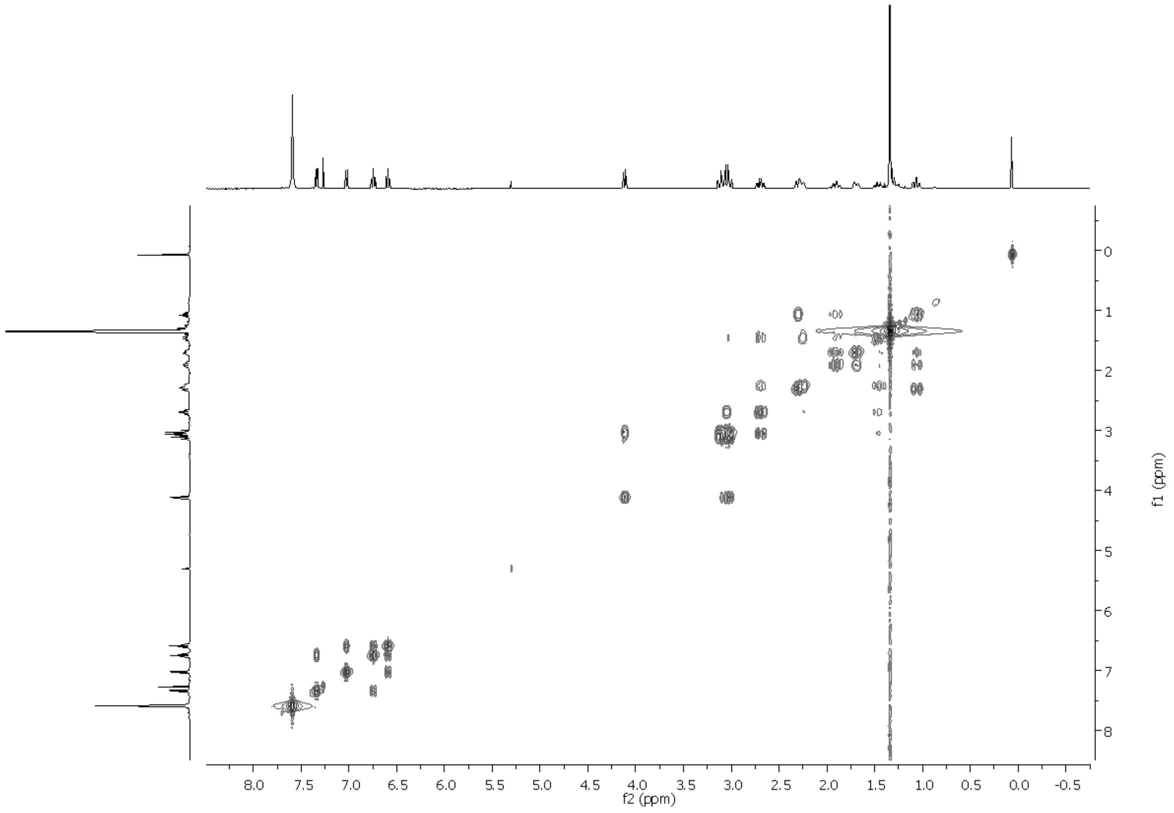

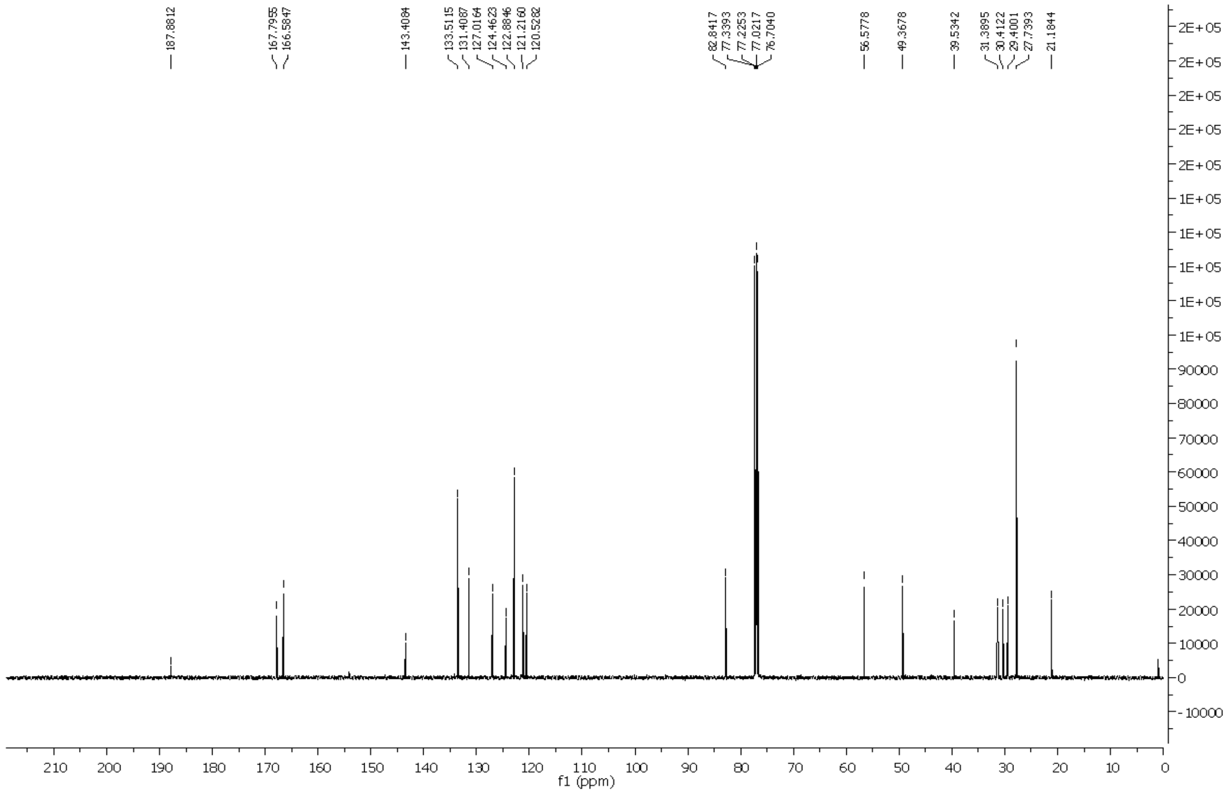
187.8812
167.7955
166.5847
143.4084
133.5115
131.4087
127.0164
124.4623
122.8846
121.2160
120.5282
82.8417
77.3393
77.2253
77.0217
76.7040
56.5778
49.3678
39.5342
31.3895
30.4122
29.4001
27.7393
21.1844
f1 (ppm)

10. Chiral HPLC chromatogram:

(*S*)-*tert*-butyl 2-(1,3-dioxoisoindolin-2-yl)-3-((*S*)-2-oxocyclohexyl)propanoate (3a)

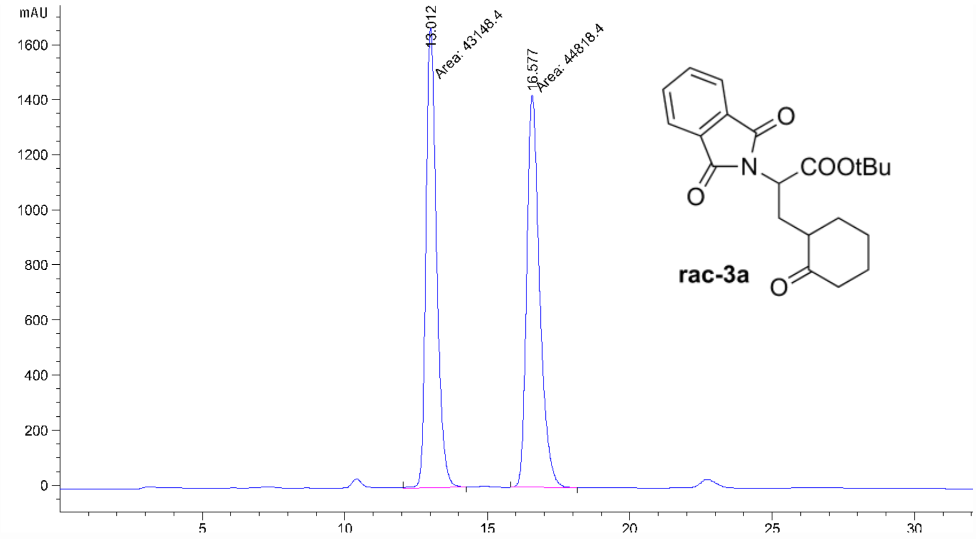

Peak #	RetTime [min]	Type	Width [min]	Area [mAU*s]	Height [mAU]	Area %
1	13.012	MM	0.4313	4.31484e4	1667.18945	49.0508
2	16.577	MM	0.5248	4.48184e4	1423.46680	50.9492

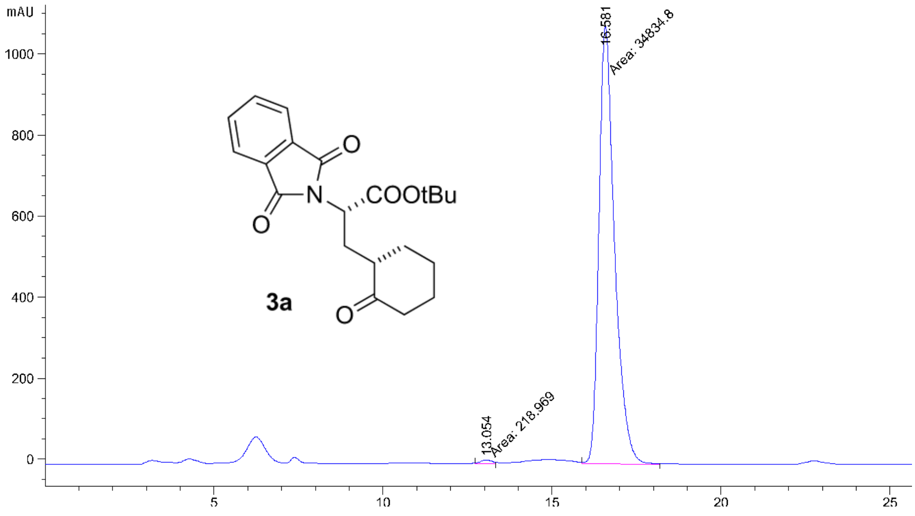

Peak #	RetTime [min]	Type	Width [min]	Area [mAU*s]	Height [mAU]	Area %
1	13.054	MM	0.3897	218.96866	9.36521	0.6247
2	16.581	MM	0.5381	3.48348e4	1078.95825	99.3753

(*S*)-*tert*-butyl 2-(1,3-dioxoisoindolin-2-yl)-3-((*S*)-2-oxocyclopentyl)propanoate (3b)

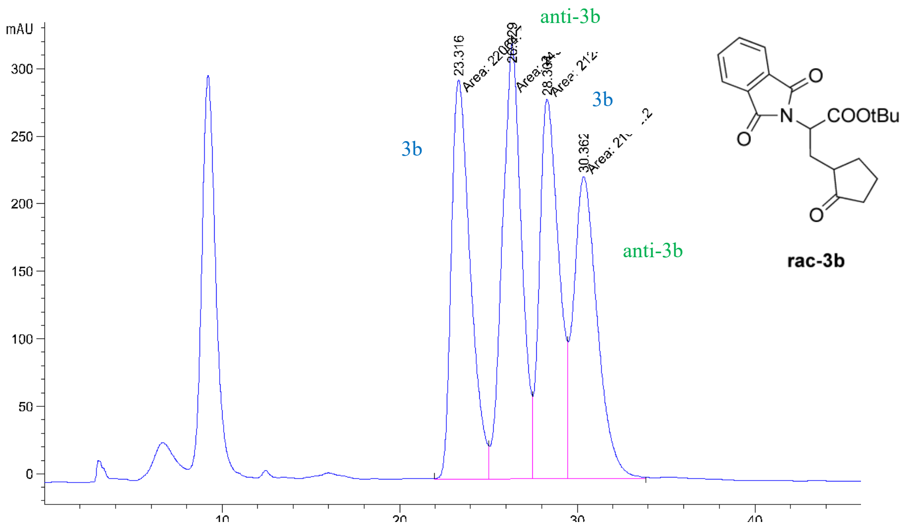

Peak #	RetTime [min]	Type	Width [min]	Area [mAU*s]	Height [mAU]	Area %
1	23.316	MF	1.2432	2.20647e4	295.80136	24.8680
2	26.329	MF	1.2590	2.43309e4	322.08182	27.4222
3	28.304	MF	1.2609	2.12494e4	280.86932	23.9492
4	30.362	FM	1.5708	2.10822e4	223.68779	23.7607

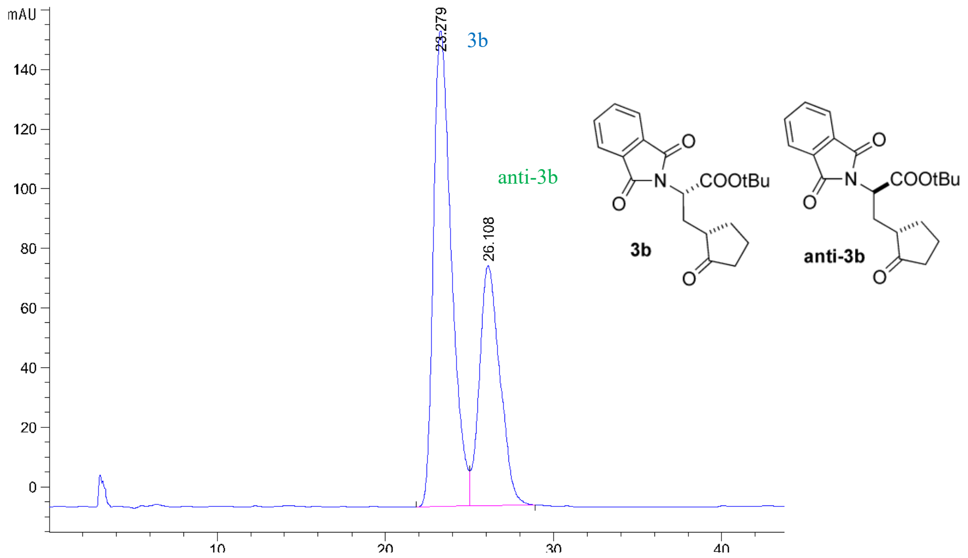

Peak #	RetTime [min]	Type	Width [min]	Area [mAU*s]	Height [mAU]	Area %
1	23.279	BV	1.1374	1.18837e4	159.64972	63.5123
2	26.108	VB	1.2665	6827.17871	80.57303	36.4877

(*S*)-*tert*-butyl 2-(1,3-dioxoisoindolin-2-yl)-3-((*S*)-4-oxotetrahydro-2*H*-pyran-3-yl)propanoate (3d)

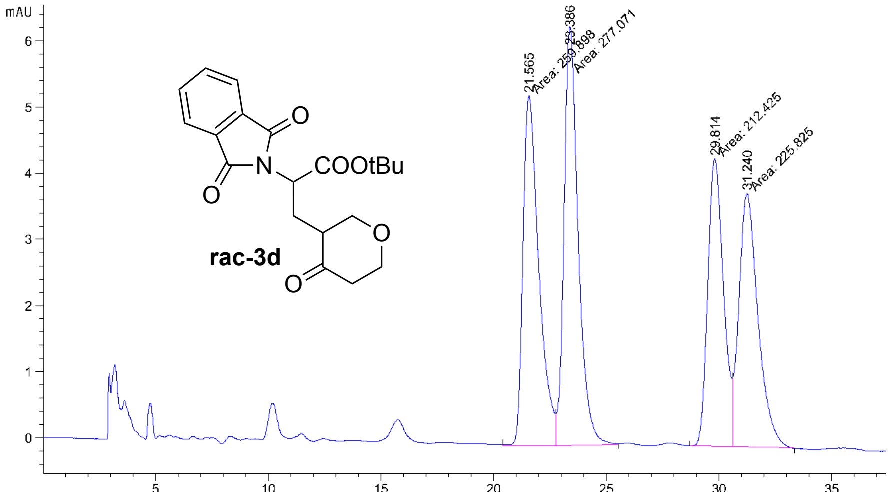

Peak #	RetTime [min]	Type	Width [min]	Area [mAU*s]	Height [mAU]	Area %
1	21.565	MF	0.8196	259.89847	5.28494	26.6502
2	23.386	FM	0.7288	277.07135	6.33651	28.4112
3	29.814	MF	0.8148	212.42493	4.34505	21.7823
4	31.240	FM	0.9828	225.82542	3.82963	23.1564

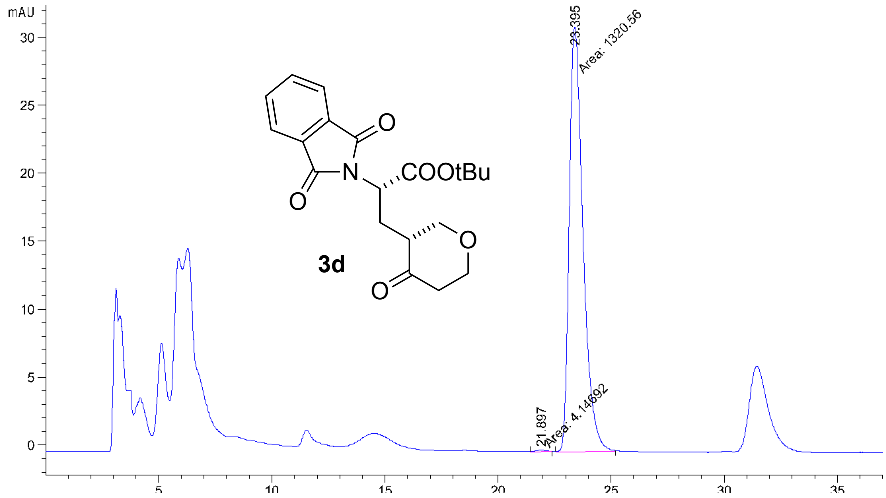

Peak #	RetTime [min]	Type	Width [min]	Area [mAU*s]	Height [mAU]	Area %
1	21.897	MM	0.5088	4.14692	9.94857e-2	0.3130
2	23.395	MM	0.7031	1320.55835	31.30515	99.6870

(*S*)-*tert*-butyl 3-((*S*)-3-*tert*-butoxy-2-(1,3-dioxoisoindolin-2-yl)-3-oxopropyl)-4-oxopiperidine-1-carboxylate (3e)

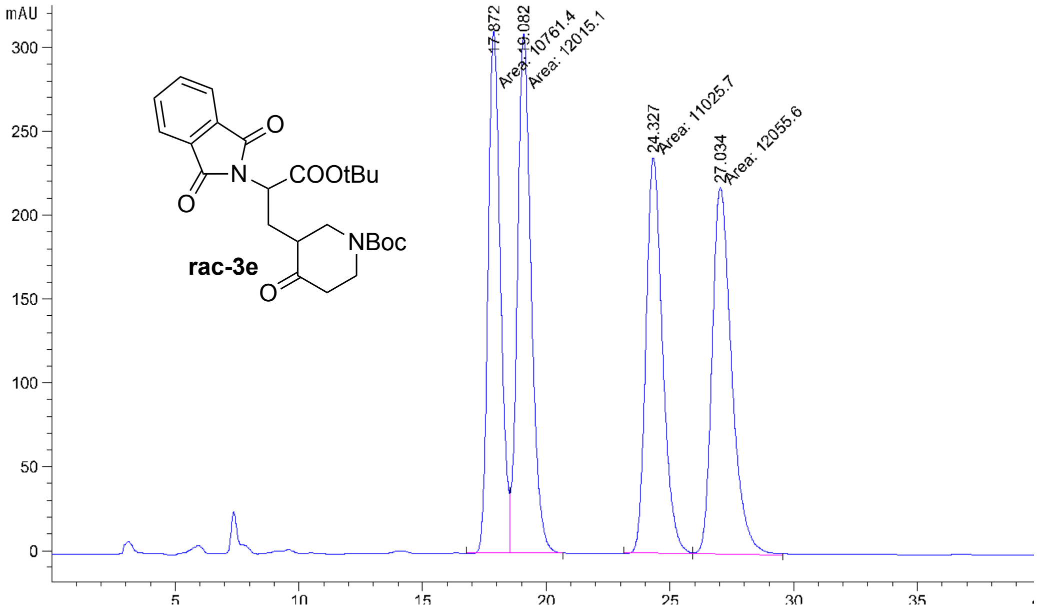

Peak #	RetTime [min]	Type	Width [min]	Area [mAU*s]	Height [mAU]	Area %
1	17.872	MF	0.5767	1.07614e4	310.99454	23.4670
2	19.082	FM	0.6473	1.20151e4	309.35449	26.2007
3	24.327	MF	0.7785	1.10257e4	236.03372	24.0432
4	27.034	FM	0.9192	1.20556e4	218.58711	26.2891

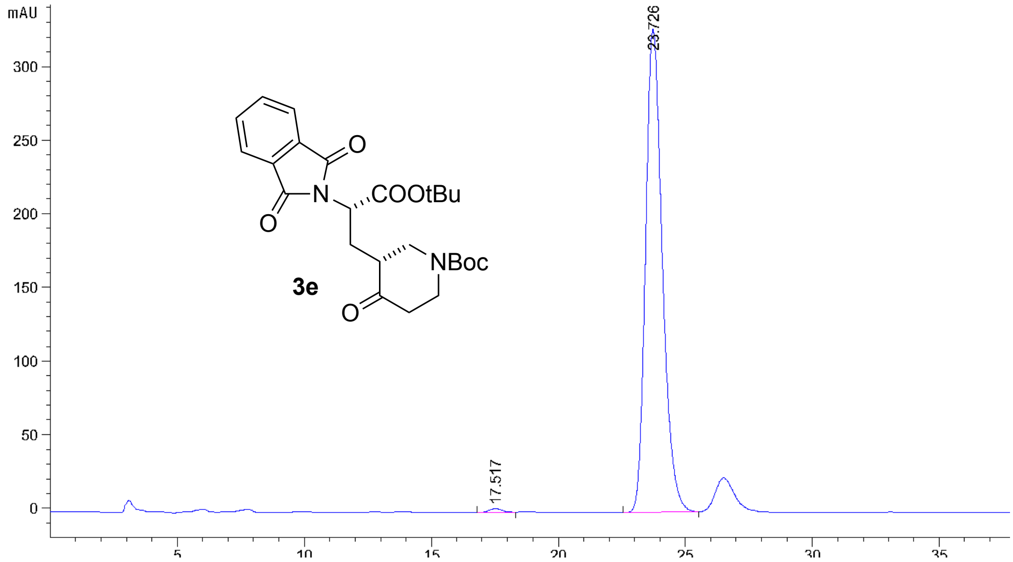

(*S*)-*tert*-butyl 2-(1,3-dioxoisoindolin-2-yl)-3-((*S*)-4-oxotetrahydro-2*H*-thiopyran-3-yl)propanoate (3f)

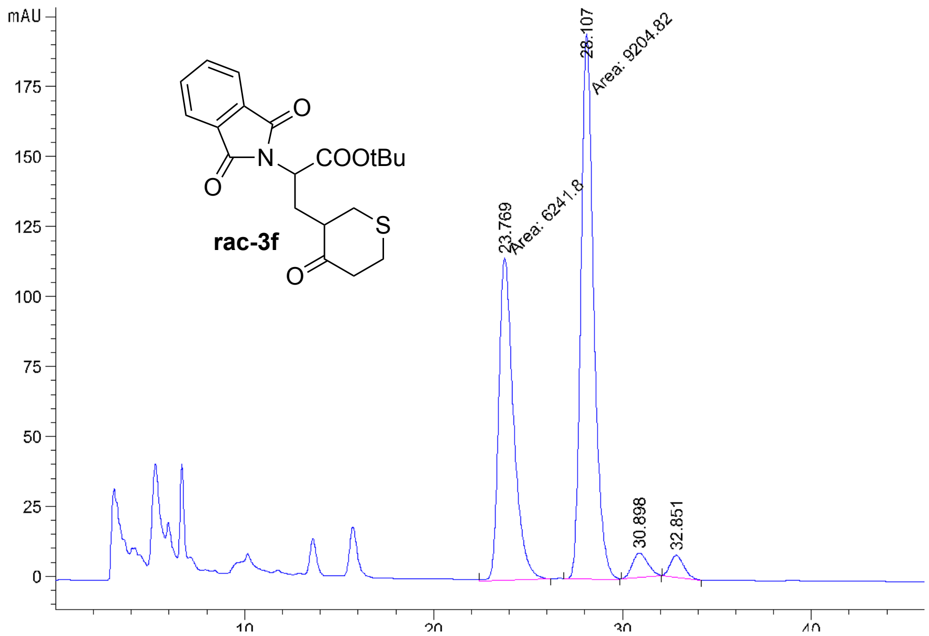

Peak #	RetTime [min]	Type	Width [min]	Area [mAU*s]	Height [mAU]	Area %
1	23.769	MM	0.9047	6241.80029	114.99380	38.2816
2	28.107	MM	0.7884	9204.82031	194.58969	56.4540
3	30.898	BB	0.8222	476.70239	8.60593	2.9237
4	32.851	BB	0.7545	381.65714	7.75058	2.3407

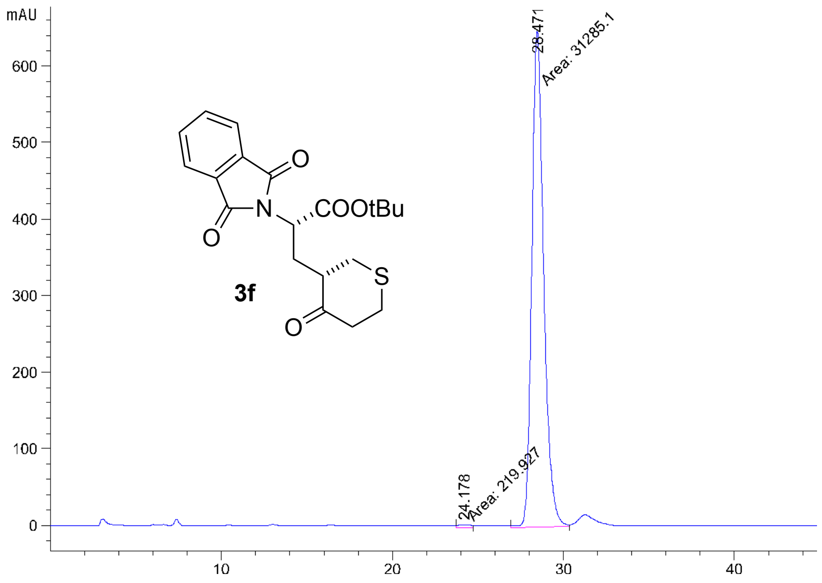

Peak #	RetTime [min]	Type	Width [min]	Area [mAU*s]	Height [mAU]	Area %
1	24.178	MM	0.9185	219.92691	3.99090	0.6981
2	28.471	MM	0.8054	3.12851e4	647.39166	99.3019

(*S*)-*tert*-butyl 2-(1,3-dioxoisoindolin-2-yl)-3-((*S*)-8-oxo-1,4-dioxaspiro[4.5]decan-7-yl)propanoate (3g)

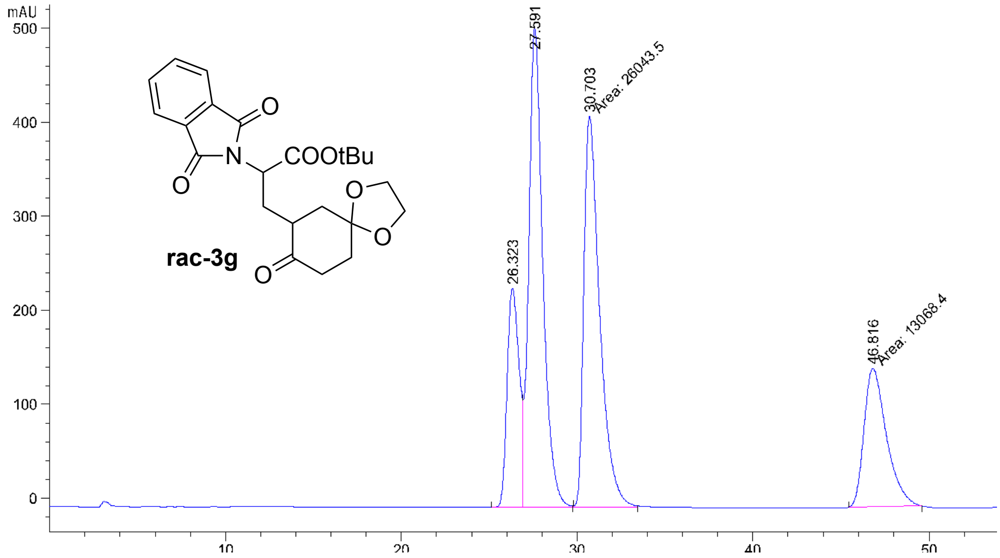

Peak #	RetTime [min]	Type	Width [min]	Area [mAU*s]	Height [mAU]	Area %
1	26.323	BV	0.7407	1.11881e4	233.19043	14.2378
2	27.591	VV	0.8275	2.82806e4	509.55701	35.9893
3	30.703	MM	1.0443	2.60435e4	415.63174	33.1424
4	46.816	MM	1.4794	1.30684e4	147.22270	16.6306

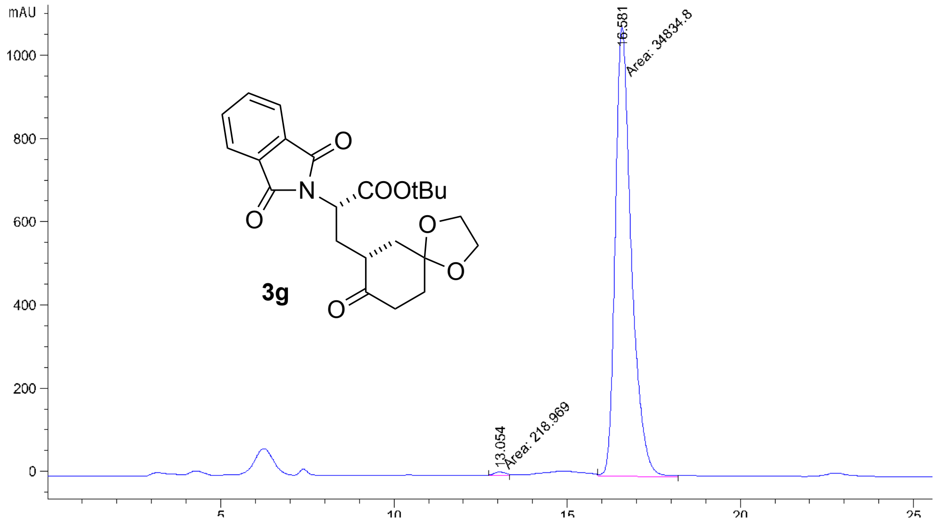

Peak #	RetTime [min]	Type	Width [min]	Area [mAU*s]	Height [mAU]	Area %
1	13.054	MM	0.3897	218.96866	9.36521	0.6247
2	16.581	MM	0.5381	3.48348e4	1078.95825	99.3753

(*S*)-*tert*-butyl 2-(1,3-dioxoisoindolin-2-yl)-3-((*S*)-3-oxotetrahydro-2*H*-pyran-4-yl)propanoate (3i)

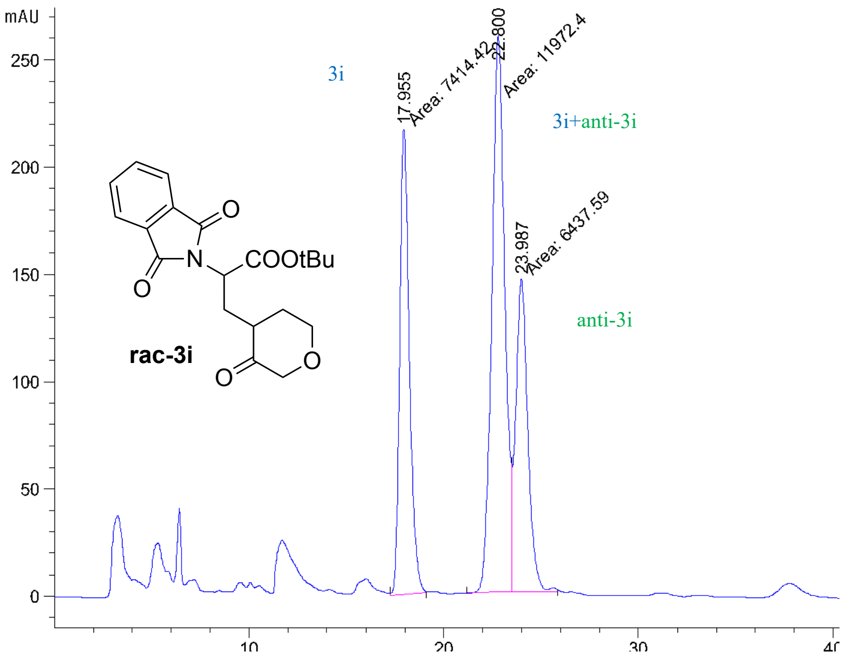

Peak #	RetTime [min]	Type	Width [min]	Area [mAU*s]	Height [mAU]	Area %
1	17.955	MM	0.5701	7414.41943	216.74530	28.7108
2	22.800	MF	0.7691	1.19724e4	259.44003	46.3609
3	23.987	FM	0.7354	6437.58887	145.90570	24.9283

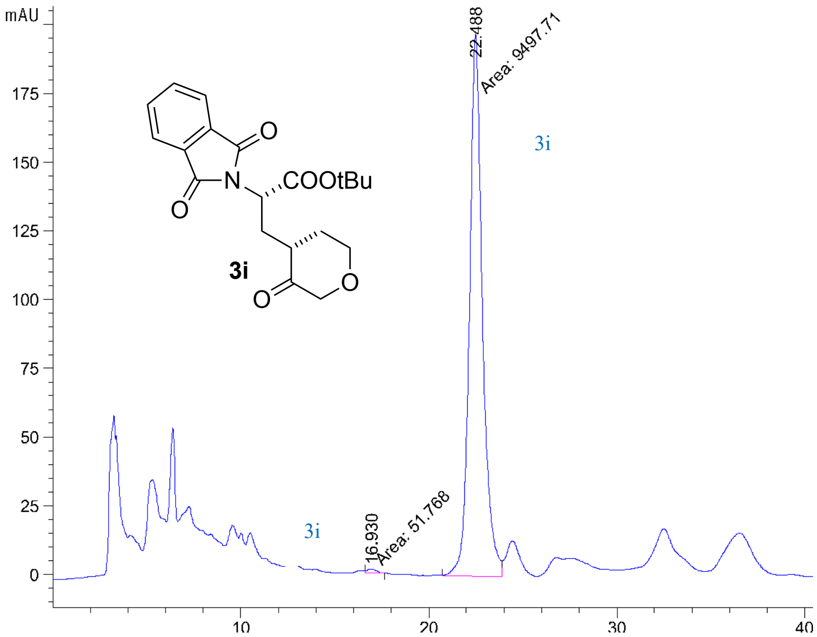

Peak #	RetTime [min]	Type	Width [min]	Area [mAU*s]	Height [mAU]	Area %
1	16.930	FM	0.5854	51.76796	1.47387	0.5421
2	22.488	MF	0.8026	9497.70898	197.23885	99.4579

(*S*)-*tert*-butyl 2-(1,3-dioxoisoindolin-2-yl)-3-((*S*)-3-oxotetrahydro-2*H*-pyran-2-yl)propanoate (3i')

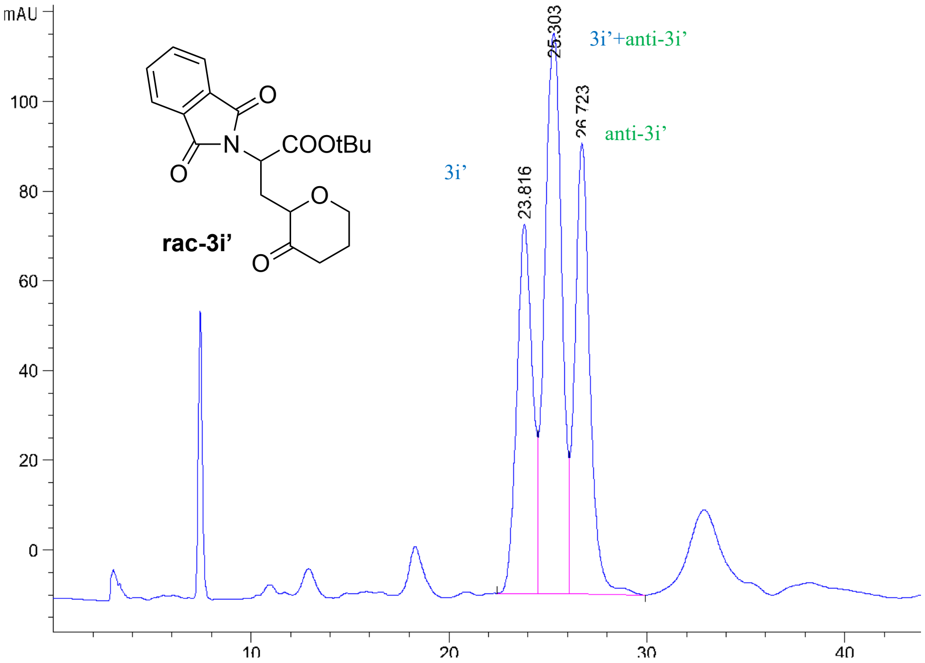

Peak #	RetTime [min]	Type	Width [min]	Area [mAU*s]	Height [mAU]	Area %
1	23.816	BV	0.8063	4512.81348	82.35951	25.9735
2	25.303	VV	0.9424	7512.42139	124.96262	43.2377
3	26.723	VB	0.7939	5349.46533	100.52127	30.7888

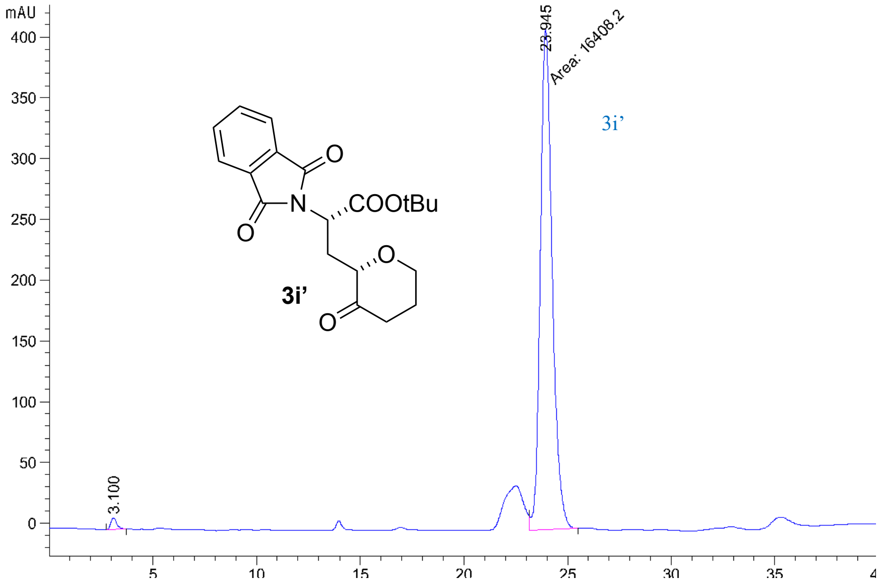

Peak #	RetTime [min]	Type	Width [min]	Area [mAU*s]	Height [mAU]	Area %
1	3.100	BB	0.3007	175.81335	8.81450	1.0601
2	23.945	FM	0.6644	1.64082e4	411.61121	98.9399

(*S*)-methyl 2-(1,3-dioxoisoindolin-2-yl)-3-((*S*)-2-oxocyclohexyl)propanoate (3j).

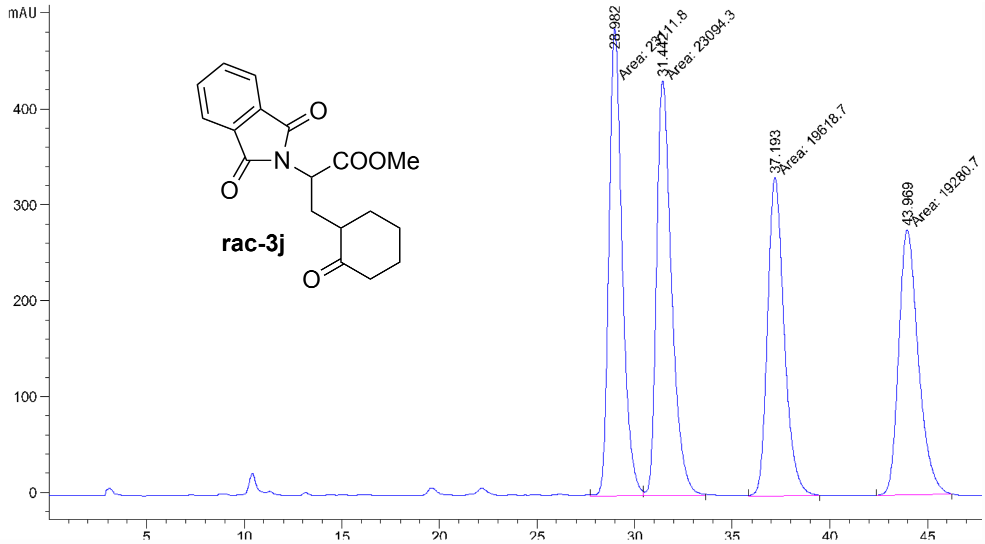

Peak #	RetTime [min]	Type	Width [min]	Area [mAU*s]	Height [mAU]	Area %
1	28.982	MF	0.7903	2.31118e4	487.39569	27.1566
2	31.442	FM	0.8893	2.30943e4	432.81360	27.1361
3	37.193	MM	0.9854	1.96187e4	331.81888	23.0522
4	43.969	MM	1.1641	1.92807e4	276.03818	22.6551

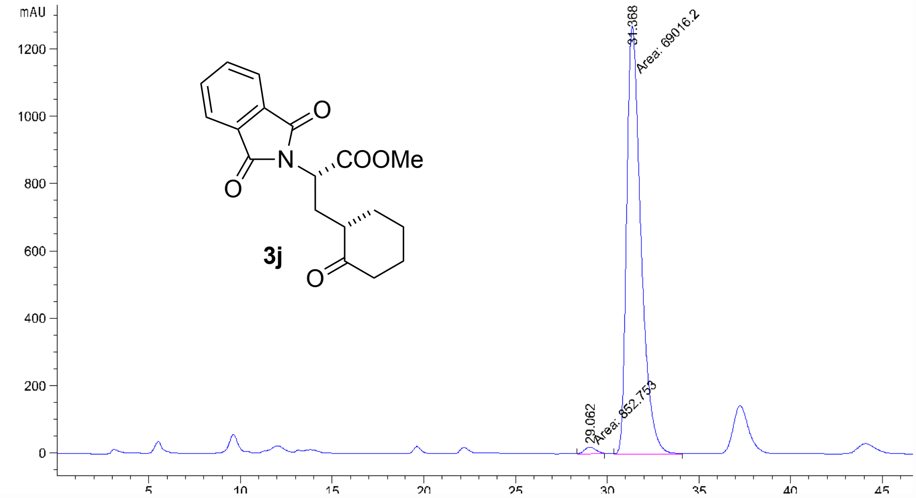

Peak #	RetTime [min]	Type	Width [min]	Area [mAU*s]	Height [mAU]	Area %
1	29.062	MM	0.7268	852.75348	19.55498	1.2205
2	31.368	MM	0.9051	6.90162e4	1270.81213	98.7795
